# Sensors Innovations for Smart Lithium-Based Batteries: Advancements, Opportunities, and Potential Challenges

**DOI:** 10.1007/s40820-025-01786-1

**Published:** 2025-05-27

**Authors:** Jamile Mohammadi Moradian, Amjad Ali, Xuehua Yan, Gang Pei, Shu Zhang, Ahmad Naveed, Khurram Shehzad, Zohreh Shahnavaz, Farooq Ahmad, Balal Yousaf

**Affiliations:** 1 Institute for Advanced Materials, School of Materials Science and Engineering, Jiangsu University, Zhenjiang, 212013 People’s Republic of China; 2 Department of Thermal Science and Energy Engineering, University of Science and Technology of China, Hefei, 230027 People’s Republic of China; 3 College of Materials Science and Engineering, Nanjing Forestry University, Nanjing, 210037 People’s Republic of China; 4 Co-Innovation Center of Efficient Processing and Utilization of Forest Resources, College of Materials Science and Engineering, Nanjing Forestry University, Nanjing, 210037 People’s Republic of China; 5 School of Materials Science and Engineering, Jiangsu University, Zhenjiang, 212013 People’s Republic of China; 6 Institute of Physics, Silesian University of Technology, Konarskiego 22B, 44-100 Gliwice, Poland; 7 Micro and Nano-Technology Program, School of Natural and Applied Sciences, Middle East Technical University, 06800 Ankara, Turkey; 8 Department of Technologies and Installations for Waste Management, Faculty of Energy and Environmental Engineering, Silesian University of Technology, 44-100 Gliwice, Poland

**Keywords:** Lithium-based batteries, Sensors, Battery management systems, Thermal runaway, State of charge, State of health

## Abstract

Lithium-based batteries (LiBs) are integral components in operating electric vehicles to renewable energy systems and portable electronic devices, thanks to their unparalleled energy density, minimal self-discharge rates, and favorable cycle life. However, the inherent safety risks and performance degradation of LiB over time impose continuous monitoring facilitated by sophisticated battery management systems (BMS). This review comprehensively analyzes the current state of sensor technologies for smart LiBs, focusing on their advancements, opportunities, and potential challenges. Sensors are classified into two primary groups based on their application: safety monitoring and performance optimization. Safety monitoring sensors, including temperature, pressure, strain, gas, acoustic, and magnetic sensors, focus on detecting conditions that could lead to hazardous situations. Performance optimization sensors, such as optical-based and electrochemical-based, monitor factors such as state of charge and state of health, emphasizing operational efficiency and lifespan. The review also highlights the importance of integrating these sensors with advanced algorithms and control approaches to optimize charging and discharge cycles. Potential advancements driven by nanotechnology, wireless sensor networks, miniaturization, and machine learning algorithms are also discussed. However, challenges related to sensor miniaturization, power consumption, cost efficiency, and compatibility with existing BMS need to be addressed to fully realize the potential of LiB sensor technologies. This comprehensive review provides valuable insights into the current landscape and future directions of sensor innovations in smart LiBs, guiding further research and development efforts to enhance battery performance, reliability, and safety.

## Highlights

- Sensors for smart Lithium-based batteries (LiBs) are classified based on their application into safety monitoring (i.e., temperature, pressure, and strain) to detect hazardous conditions and performance optimization (i.e., optical and electrochemical sensors) for monitoring factors such as state of charge and state of health.
- The potential for innovation in LiB sensor technology is driven by advancements in nanotechnology, miniaturization, machine learning algorithms, and wireless sensor networks, all of which contribute to enhanced sensor performance.
- Key challenges faced in developing LiB sensors include miniaturization, power consumption, cost efficiency and scalability, and compatibility with existing battery management systems.

## Graphical abstract

**Figure:**
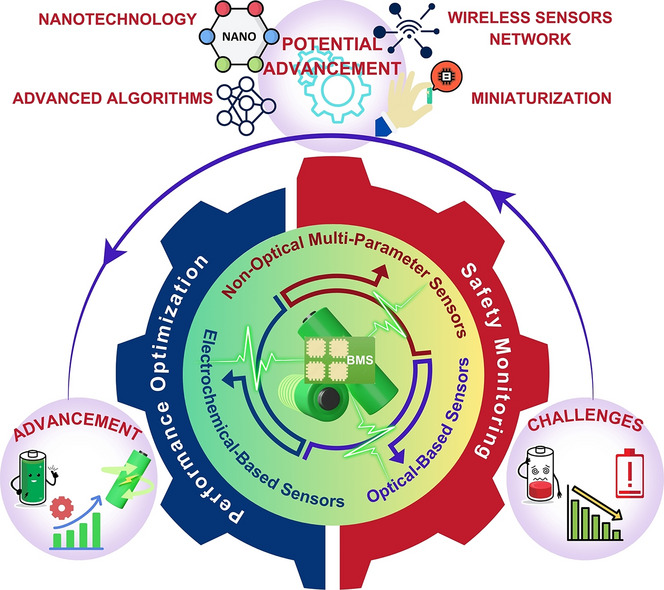
Integration of advanced sensor technologies for smart LiBs: integrating non-optical multi-parameter, optical-based, and electrochemical sensors within the BMS to achieve higher safety, improved efficiency, early warning mechanisms, and TR prevention. Potential advancements are driven by nanotechnology, wireless sensor networks, miniaturization, and advanced algorithms, addressing key challenges to enhance battery performance and reliability.

## Introduction

The rapid expansion of energy storage demands, driven by electric vehicles (EVs), renewable energy integration, and portable electronics, has positioned lithium-based batteries (LiBs) as the cornerstone of modern energy solutions [1–3]. Renowned for their superior energy density, extended cycle life, and minimal self-discharge compared to alternatives such as lead-acid or nickel-metal hydride batteries [4–7], LiBs nonetheless face critical challenges. Safety risks, performance degradation at elevated temperatures (> 50 °C), and capacity fade over time underscore the urgent need for advanced battery management systems (BMS) to optimize efficiency and mitigate hazards [8–11]. In a related study, Yang et al. [12] investigated the thermal characteristics of a hybrid solid–liquid battery (solid-state battery) and its significance for the development of future BMS. The study found that the solid-state battery exhibited a higher polarization resistance than traditional NMC (Lithium nickel manganese cobalt oxide, LiNiMnCoO_2_) and LFP (Lithium iron phosphate, LiFePO_4_) batteries with similar capacity. The higher resistance resulted in more heat generation and a higher temperature rise in the solid-state battery, necessitating a BMS with stronger cooling capabilities. Their study also found that reversible heat is the primary cause of the temperature rise plateau in solid-state batteries, while polarization heat is the predominant factor in total heat generation [12].

Central to this evolution is sensor innovations, which redefine the capabilities of BMS by enabling real-time monitoring of temperature, pressure, mechanical strain, etc. These advancements enhance safety through early failure detection, such as internal temperature spikes or electrode bulging, and unlock opportunities for adaptive energy management [1, 13]. For example, sensors such as micro-thin-film have demonstrated potential in detecting early indicators of failure, such as mechanical pressure shocks, battery bulging, and internal temperature changes [14]. These sensors can capture real-time piezoelectric and pyroelectric responses, providing valuable insights into battery health and significantly improving BMS safety by offering early warnings of potential battery damage and preventing catastrophic failures [14]. Similarly, fiber Bragg grating (FBG) sensors have been explored to monitor mechanical strain and distortion in LiBs and all-solid-state batteries. These sensors, embedded within battery electrodes, offer real-time performance data, which can significantly improve battery durability and safety [1].

Despite these strides, a comprehensive analysis of sensor-driven advancements in smart LiBs, systems capable of autonomous adaptation via real-time data, remains absent in the literature. The unique aspect of this review lies in its focus on the potential benefits of sensor innovations for improving the overall performance of smart LiB. Advanced algorithms and control approaches can be employed to optimize charging and discharge cycles by obtaining detailed information on battery usage patterns and behavior. This approach not only improves the overall performance and reliability of LiB but also ensures the efficient utilization of energy stored within batteries. This optimization of energy usage is crucial for ensuring the durability and efficiency of LiB, contributing to the sustainability and cost-effectiveness of energy consumption. However, there are potential challenges related to sensor innovations in smart LiB. These challenges include sensor miniaturization, power consumption, cost efficiency, and compatibility with the existing BMS. Addressing these challenges is critical for effectively controlling the full potential of LiB sensor technologies. By comprehensively studying advancements and exploring opportunities and challenges, this review article aims to provide insights into the potential of sensor innovations in smart LiB systems. The insights gained from this study can guide further research and development, facilitate the integration of advanced sensor technologies, and drive future advancements in energy storage systems for more reliable, efficient, and smart LiB systems.

In this review article, the LiB cells typically discussed have a nominal voltage of 3.6–3.7 V per cell. The nominal capacity of individual cells commonly differs from approximately 1500–15,000 mAh, depending on the specific LiB cell type and its intended application, as discussed in the literature [15–18]. The anode electrode is typically made of graphite or LiTi (lithium titanate, Li_4_Ti_5_O_12_), facilitating the release and acceptance of lithium (Li) ions during charging and discharging cycles. Conversely, the cathode electrode is composed of Li metal oxides, such as LCO, LMO (lithium manganese oxide, LiMn_2_O_4_), NCM (lithium nickel manganese cobalt oxide, LiNiCoMnO_2_), LFP, or NCA (lithium nickel cobalt aluminum oxide, LiNiCoAlO_2_). These cathode materials enable the insertion and extraction of Li-ions during the charge and discharge processes. Moreover, the electrolyte material typically consists of a Li salt dissolved in an organic solvent, commonly comprising Li hexafluorophosphate (LiPF_6_) in a mixture of ethylene carbonate (EC) and diethyl carbonate (DEC) [15–19].

## Sensors Used in Smart LiBs Monitoring

**Fig. 1:**
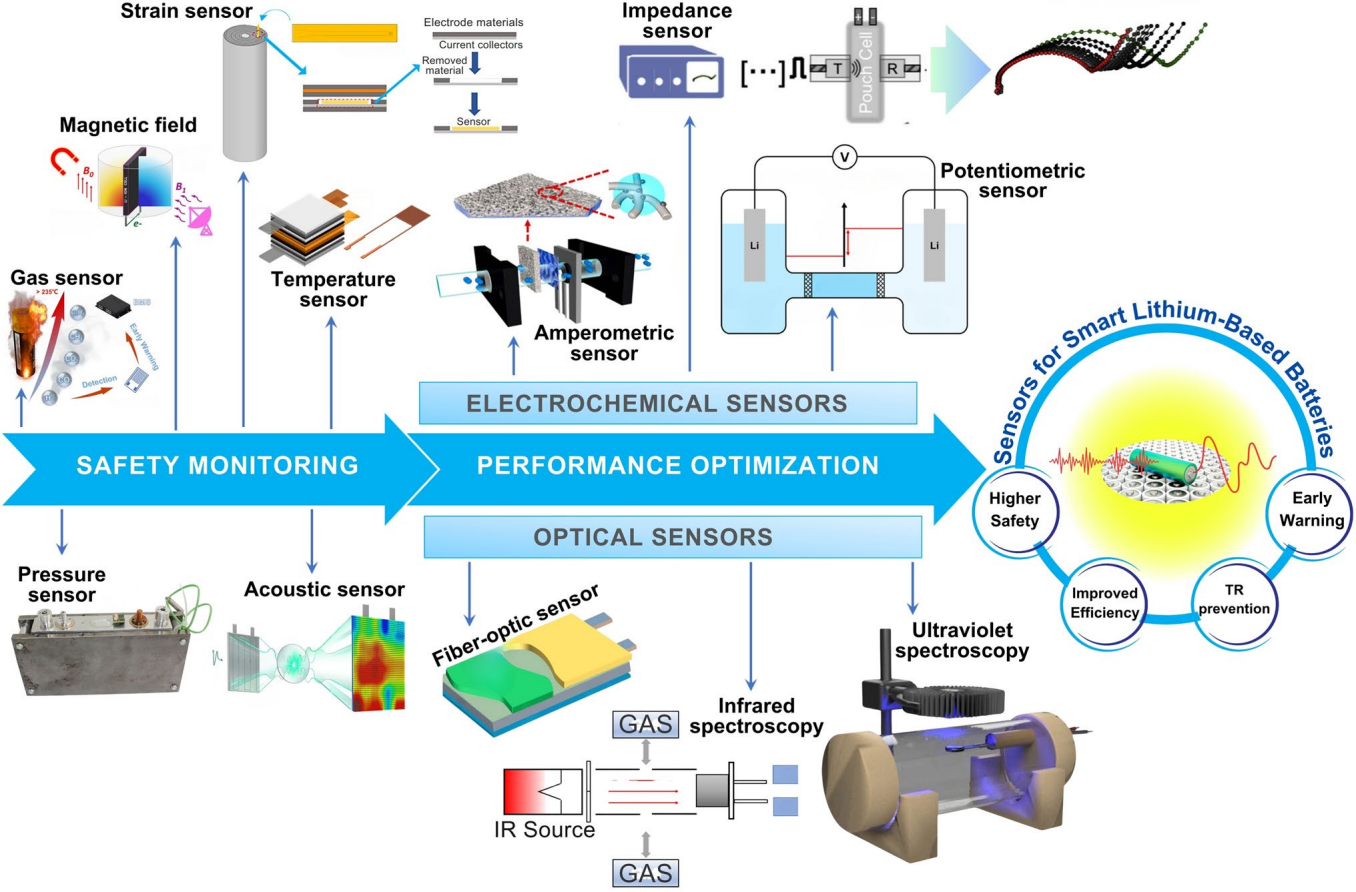
Classification and contribution of sensor technologies based on application principles for smart LiBs monitoring. The insets include panels reproduced with permission for Magnetic field, ref. [20], from ACS; Gas sensor, ref. [21], from ACS; Acoustic sensor, ref. [22], from ACS; Pressure sensor, ref. [15], from Elsevier; Temperature sensor, ref. [23], from Elsevier; Strain sensor, ref. [24], from Elsevier; Fiber-optic sensor, ref. [25], from Springer Open; Ultraviolet spectroscopy, ref. [26], from Elsevier; Infrared spectroscopy, ref. [27], from MDPI; Amperometric sensor, ref. [28], from ACS; Potentiometric sensor, ref. [29], from ACS; Impedance sensor, ref. [30], from Cell Press

The effective monitoring of LiBs relies on various sensor types, broadly classified based on application into two primary groups, including safety monitoring and performance optimization (Fig. 1). Sensors dedicated to safety monitoring (non-optical multi-parameter sensors) primarily focus on detecting conditions that could lead to hazardous situations, such as thermal runaway (TR), gas generation, or structural deformation. These include sensors, such as temperature sensors, gas sensors, strain sensors, that provide early warnings of potential failures. On the other hand, sensors aimed at performance optimization monitor factors such as state of charge (SoC) and state of health (SoH) and emphasize enhancing the battery’s operational efficiency, lifespan, and overall output. This class typically includes electrochemical-based and optical-base sensors, which offer insights into the battery’s internal state and support more accurate control strategies. This section aims to comprehensively explore the application principles inherent in each sensor type, elucidating the advantages and limitations driving their performance.

### Sensors for Safety Monitoring

Safety monitoring sensors encompass a range of physical measurements, including temperature, pressure, mechanical stresses, etc., preventing catastrophic events such as TR and ensuring the battery's structural integrity. These sensors are well-established technologies commonly used in various LiB industries and research domains. They are typically considered standard or conventional solutions in a given LiB monitoring. However, integrating physical measurements, such as strain, acoustic, and magnetic data, with electrochemical performance metrics significantly enhances diagnostic precision and supports adaptive battery management. This integration facilitates real-time monitoring and performance optimization of battery systems. For example, correlating strain, acoustic, and magnetic measurements with electrochemical impedance spectroscopy (EIS) offers valuable insights into internal battery dynamics, including charge transfer resistance, double-layer capacitance, and solid electrolyte interphase (SEI) characteristics during charging and discharging cycles. These correlations aid in assessing parameters such as the SoH, SoC, and the detection of potential internal short circuits within batteries.

#### Temperature Sensors

**Fig. 2:**
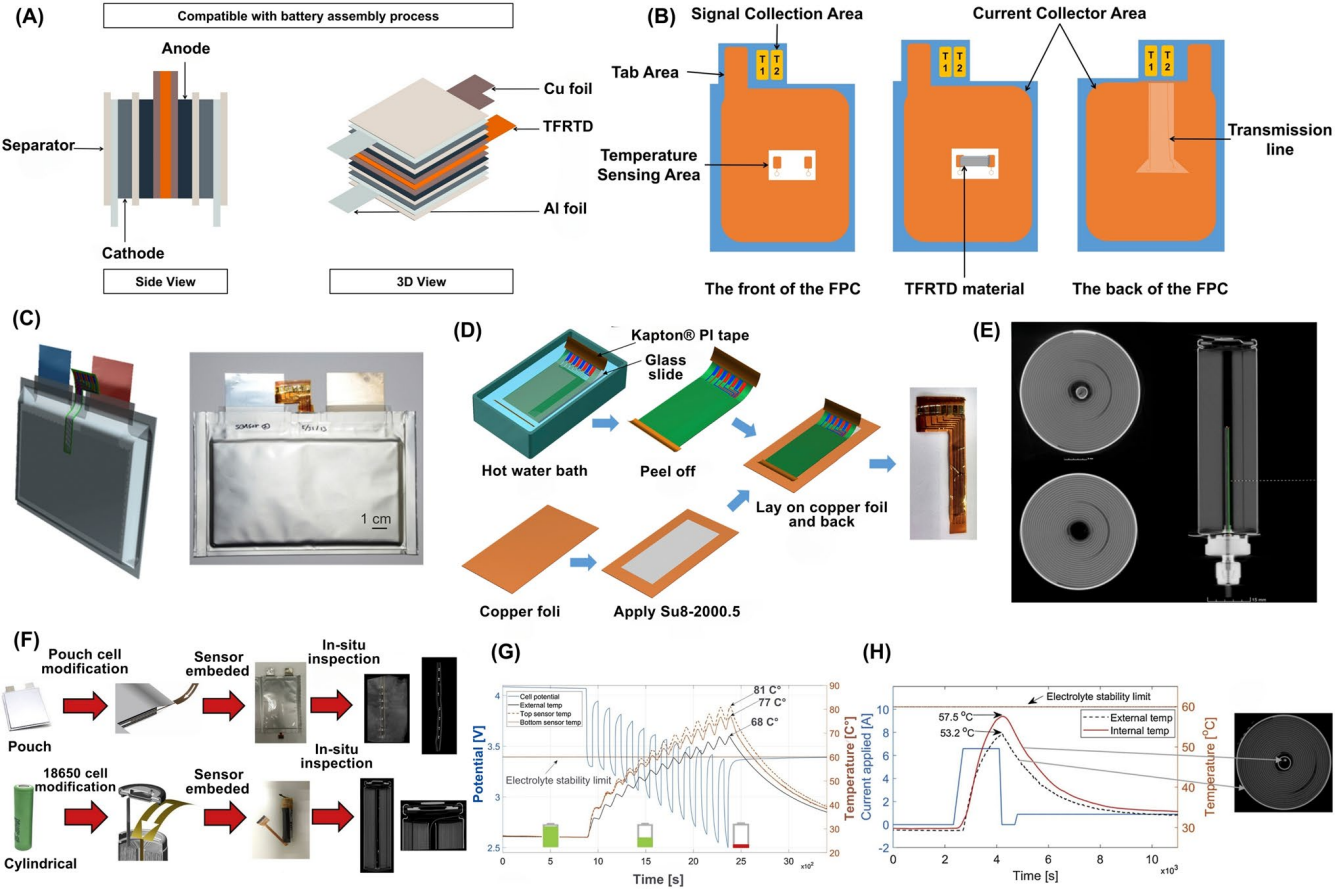
Schematic illustrating the integration of LiB with temperature sensors. **A** The assembly process for the pouch LiB with integrated TFRTD. **B** The structure and manufacturing sequence of the flexible printed circuit (FPC) are from both its front and rear sides. The diagram also outlines the process of depositing TFRTD materials. The front structure of the FPC is presented as a composite of multiple layers. **C** Pouch cell integrated with a TFTC, **D** Steps involved in transferring TFTC onto Cu foil coated with SU8 2000.5. The TFTC embedded in polyimide is secured with Kapton® PI tape along its edges. The setup is immersed in a warm water bath to facilitate the removal of the PI-embedded TFTC, followed by the transfer of the detached TFTC onto the SU8 2000.5-coated Cu foil. **E** The Computed tomography (CT) images of an instrumented cell and its structure, including top-view images of the negative terminal post-instrumentation, the positive terminal from the top perspective, and a side view of the instrumented cell. **F** The process of fabricating smart LiB cells encompasses both pouch and cylindrical cell variants. The depiction progresses from the initial unmodified cell to the ultimate instrumented smart cell stage, with a focus on the insertion of the sensor. (Panel **F** also presents real-time X-ray images of the fully instrumented cells). **G** In situ examination of a LiB cell under high current load, highlighting fluctuations in skin temperature. The pronounced pulse discharge simulates the irregular acceleration patterns of an EV until the batteries are fully discharged. **H** High charge current, which holds significance in developing rapid charging profiles. The top-view X-ray image of a cell equipped with instrumentation visually depicts the increasing temperature differential between the cell’s internal and external environments. Panels reproduced with permission from** A**, **B**, ref. [23], Elsevier; **C, D**, ref. [42], Elsevier; **E**, ref. [40], Elsevier; **F–H**, ref. [57], Elsevier publishing

It has been reported that when a single LiB experiences overcharging, short-circuiting, or other abusive conditions, it can generate a substantial amount of heat in a short period. Once the battery temperature reaches between 100 and 130 °C, the separator starts to melt, which can lead to an internal short circuit. This increase in internal temperature accelerates the chemical reactions rather than the desired galvanic reactions, leading to additional heat production, which can degrade the battery components and increase the risk of TR, posing a significant hazard of fire or even explosion [31, 32]. Once TR occurs in a single cell within a battery module, the heat can spread to neighboring cells through thermal conduction, potentially causing TR of the entire battery module. Compared to a single cell, the TR of an entire battery module generates significantly more heat and presents a greater risk, which can lead to catastrophic fire or explosion [32]. However, recent reports indicate that Li metal batteries have successfully operated within a high-temperature range of 90–170 °C. This range exceeds the decomposition point of organic electrolytes (90 °C) while remaining below the melting point of Li metal, which is approximately 180 °C [33]. On the contrary, at low temperatures, LiBs exhibit diminished discharge capacity and may even fail to discharge, which significantly impedes the development of batteries [34]. The poor performance of LiBs at low temperatures is associated with factors such as reduced electrolyte conductivity, slow charge transfer kinetics, increased resistance due to high energy barriers for Li-ion desolvation and Li-ion migration within the SEI, and sluggish Li diffusion through the surface layers and within the bulk of active material particles (i.e., graphite anodes) [35–37]. It has been reported that when the temperature drops below 0 °C, Li-ion diffusion is significantly reduced, leading to Li-ion depletion and severe dendrite formation [38]. The in situ temperature monitoring in BMS for EVs and renewable energy systems can prevent rapid degradation and ensure optimal battery operation and safety [39, 40]. These sensors can be placed on the surface of a LiB or embedded within the battery cells [14, 40–42]. Thermocouples (TC), thermally sensitive resistors (i.e., thermistors), and resistance temperature detectors (RTD) are among the typically used temperature sensors for LiB monitoring (Table 1) [2, 13, 43, 44]. In a study, Ling et al. fabricated a thin-film sensor using a copper/nickel (Cu/Ni) alloy to develop a high-throughput thin-film resistance temperature detector (TFRTD) (Fig. 2A, B). The devised TFRTD exhibited significant potential for real-time monitoring of internal LiB heating within the range of 30–80 °C at different current rates. The comparative analysis demonstrated that the TFRTD delivered 82% faster and achieved a 33% accuracy in temperature measurement compared to external RTDs, enabling more accurate tracking of dynamic thermal changes [23]. These enhancements in speed and accuracy suggest the TFRTD is a promising alternative for internal LiB temperature monitoring, with implications for optimizing battery performance and safety. Although this study indicated potential enhancements, the long-term durability and reliability of the Cu/Ni alloy used in the TFRTD sensor may be affected by several factors. For example, the repeated lattice expansion/contraction, often termed breathing, of electrodes during cycling induces mechanical stress, which may compromise the structural integrity of the TFRTD elements and their electrical connections. Other factors, such as mechanical vibration, temperature gradients, and electrolyte exposure, could degrade solder joints, wiring, or the alloy itself. While platinum can be prone to corrosion in certain RTDs [45], the compatibility of RTD materials, such as Cu/Ni alloys, with battery electrolytes under operational conditions requires thorough validation to prevent potential cell contamination. Moreover, RTDs necessitate precise placement to accurately map thermal gradients within the battery, which can complicate system integration and increase costs [46]. Periodic recalibration is also required to mitigate measurement drift, adding to maintenance demands.

**Table 1:** Key characteristics of temperature sensors for safety monitoring in smart LiBs

Sensor type	Sensitivity	Accuracy	Durability (thermal/Mech.)	Cost	Response time	LiB cell type/Cathode chem	Integration complexity	Power consumption	Typical applications	References
Custom miniature TC (K-type)	High (~ 0.03 ℃ resolution)	± 0.143 ℃ (calibrated)	High (thermal)	Low	Fast (~ 0.7 s for surface sensors)	21,700 cylindrical cells, NMC811	Moderate (requires modification)	Low	Internal temperature monitoring in cells, particularly for TR detection	[40]
TC (K-type)	High (precise, linear)	± 0.1 ℃	High (mechanical and thermal resistance)	Low	Fast	Pouch, prismatic, and cylindrical cells, NCM	Moderate (requires modification)	Low	Internal temperature monitoring during TR tests	[47]
Flexible Thin Film Thermocouples (TFTC)	41.2 μV ℃^−1^	± 0.1 ℃	High (survives battery assembly process, stable in electrolyte environment)	Moderate (based on material cost)	N/A (effective in real-time monitoring)	Pouch, NMC, and cylindrical cells	High (requires integration into the battery assembly process)	Low	In situ temperature measurement during high-rate charge/discharge cycles	[42]
TC, K-type (internal core)	N/A	± 0.1 ℃	High (mechanical robustness, can withstand extreme conditions)	Low	Fast	Cylindrical (26,650) LFP	High (requires core insertion and modification)	N/A	Measuring core temperature for validation of impedance-based internal temperature estimation	[48]
TC, K-type, (embedded)	N/A	± 0.1 ℃ (core)	High (mechanically robust)	Low	Fast	Cylindrical (26,650), LFP	High (requires insertion into the core)	N/A	Core temperature validation and comparison with non-invasive IR method	[49]
TC, K-type	N/A	± 0.17 ℃	High (self-powered, robust, small size)	Low	Moderate (~ 1.2 times slower rise time than FBGs)	Cylindrical, LFP	Moderate (requires attachment to surface)	Low	Temperature monitoring for normal and abusive conditions, widely used in commercial applications	[50]
TC, T-type	Moderate (sensitive to surface heat flux)	± 0.1 ℃	High (mechanically robust)	Low	Fast	Cylindrical 18,650, LCO	Moderate (requires a placement on the cell surface)	Low	External temperature monitoring during TR	[51]
TC, K-type	High (sensitive to temperature changes)	± 1 ℃ (measurement error)	High (robust for high C-rates)	Low	Moderate	Pouch and cylindrical cells, NMC	Moderate (requires embedding inside the cell)	Low	Internal temperature monitoring, used for validating thermographic data	[52]
TC, T-type (Custom Miniature)	High	N/A	Excellent for thermal environments, robust	Low	Fast	18,650, 21,700 Cylindrical	Medium to High (Requires insertion)	N/A	Measuring core temperature, TR detection	[13]
TC, K-type	High (detects sharp ΔT to 1045 ℃)	N/A	High (tested up to ~ 1045 ℃)	Low	Fast	Large-format pouch, NCM811	Moderate (needs multiple placements)	Low	Surface temperature monitoring during overcharge and explosion	[53]
TC, K-Type (× 2)	Moderate-High (± 0.4%)	Moderate (~ 0.4% error)	High (up to > 150 ℃ measured)	Low	Fast (real-time logging)	Cylindrical, LFP (18,650, 26,650, 26,700)	Low (surface-mounted)	Low	Battery surface temperature monitoring	[54]
Thermistor	Moderate (smoothed by airflow)	N/A	Moderate (standard automotive grade)	Low	N/A	Prismatic cells, LFP	Low (easily integrated into packs)	Low	External temperature measurement in battery modules	[55]
Thermistor	High (0.1 ℃)	~ 0.1 ℃	Moderate (thermal)	Low (~ $0.01 each)	Fast	21,700 cylindrical cells	Moderate (requires flexible PCB)	Low	In situ cell temperature sensing, pack monitoring	[56]
NTC Thermistor	High (precise, linear)	± 0.1 ℃ (stable)	High (mechanical/thermal)	Low	Fast	Pouch (LCO) and Cylindrical (NCA)	Moderate (flexible, solderable)	N/A	In situ thermal monitoring, safety mapping	[57]
NTC Thermistor	Moderate (linear sensitivity)	± 1% (0–100 ℃ range)	Moderate (automotive-grade)	Low	Slow	NMC (20 Ah), LFP (14 Ah), LTO (5 Ah)	Low (simple contact setup)	Low	Surface temperature monitoring during charge/discharge cycles	[44]
NTC Thermistor (Embedded)	High (near linear beta curve)	± 1% to ± 2%	High (mechanical, chemical, and thermal stability)	Moderate	Moderate	Pouch cells (LCO), Cylindrical (NCA)	High (requires embedding and protection)	Low	Long-term in situ thermal monitoring of LiB cells	[57]
Thermistor (Internal)	Moderate (Linear, adjustable)	N/A	High (stable under various conditions)	Low	Fast	Cylindrical 18,650, LCO	Moderate (requires insertion into cell)	Low	In situ temperature monitoring during high-rate discharge and charge cycles	[58]
NTC Thermistor	Moderate (sensitive to surface temperature)	± 1 ℃	Moderate (suitable for surface measurements)	Low	Fast	18,650 cells (NMC/Graphite, LFP)	Low (surface mountable)	Low	Surface temperature monitoring in 18,650 cylindrical cells	[52]
Thermistor Array	Medium	N/A	Good for monitoring gradients	Low	Moderate	18,650 Cylindrical, Pouch cells	Medium (Requires distributed array)	N/A	Thermal behavior monitoring, in situ temperature sensing	[13]
Pt-100 RTD	High (precise temperature measurement)	N/A	High (stable and reliable in cycling)	Low	Fast	Cylindrical 18,650, NCM	Low (simple contact setup)	Low	In situ temperature measurement during cyclic charge/discharge of LiB cells	[59]
Cu/Ni Thin-Film RTD (TFRTD)	High (α_20_ = 0.00415 at 20 ℃)	Max error ± 0.83 ℃; avg ± 0.35 ℃	High (thin, stable in electrolyte)	Low (Cu/Ni, low-cost materials)	Fast (avg 6.5 s vs. 26.2 s for Pt1000 at 60–80 ℃)	Pouch cell, LCO	Moderate (compatible with cell assembly)	N/A	In situ internal temperature monitoring to prevent TR	[23]
External Pt-1000 RTD (comparison)	Medium (α_20_ = 0.00297)	Max error ± 1.24 ℃; avg ± 0.65 ℃	Moderate (decreased by substrate)	Moderate	Slower (avg 14–26 s)	Pouch cell, LCO	Low (external placement)	N/A	External reference for benchmarking internal sensor	[23]
PT1000 RTD (Embedded)	High (0.003851 ± 0.000004 Ω/Ω/℃)	High	High (− 50 ℃ to 400°C, stable in electrolyte with PI coating)	Moderate	Fast	Coin cell (CR2032), LMO	Moderate (embedded in coin cell with insulation)	N/A	Real-time internal temperature monitoring for safety and thermal analysis	[60]

The implementation of flexible polymer-embedded thin-film thermocouples (TFTC), developed from K-type TC, in a LiB pouch cell could provide a promising and scalable solution for monitoring the in situ temperature during high-rate charge and discharge cycles (Fig. 2C, D) [42]. A study by Gulsoy et al. examined the performance and stability of in-cell instrumentation with embedded TC for temperature measurements (Fig. 2E) [40]. The cells were modified to incorporate a threaded hole for sensor insertion, and custom fittings were designed to secure the sensors within the cells. The results indicated that the instrumentation process did not adversely affect cell performance, and the embedded TC provided stable and accurate internal temperature measurements [40]. Their findings revealed that the internal temperature consistently surpassed the surface temperature during cell characterization, even under electrical loading, with a dynamic real-world profile derived from EVs [40]. While TCs have a wide temperature range and fast response times, they are susceptible to calibration drift over time, particularly due to the mechanical and chemical stresses within the battery environment. Mechanical vibration, temperature cycling, and electrolyte exposure can lead to oxidation, corrosion, or chemical reactions in TC metals (e.g., nickel, platinum, and chromium) and their connections. These factors can result in reduced sensor lifespan, compromised measurement accuracy, and pose a risk of heavy metal contamination during battery recycling, as integrated sensors are difficult to separate [2]. Embedding TCs requires invasive and direct contact procedures that may affect battery integrity [61]. Furthermore, their reliance on a reference temperature and susceptibility to electrical noise can complicate accurate temperature readings, posing challenges for seamless integration with BMS [2]. Raijmakers et al. reported that the accuracy of TC may not be as high as that of other sensors, and achieving accuracy better than 1 ℃ poses a significant challenge [62]. Furthermore, although embedded TC may be effective for individual battery cells, extending their application to larger battery systems poses significant challenges, necessitating additional engineering efforts and cost considerations [2, 63].

Fleming et al. developed a method for embedding flexible, distributed thermistor sensors within commercial battery cells, both pouch and cylindrical cells, which facilitates in-situ and operando temperature data collection (Fig. 2F) [57]. Their study utilized raw negative temperature coefficient (NTC) thermistor elements encapsulated in a protective Parylene C coating. The embedded thermistors provided critical insights, revealing significant internal temperature gradients and identifying instances where core temperatures exceeded surface measurements, posing potential safety risks. Furthermore, their study allowed charging currents substantially higher than manufacturer recommendations while significantly reduced charging times without compromising safety limits, enabling the collection of long-term in-situ and operando thermodynamic data (Fig. 2G, H) [57]. Despite the high sensitivity and rapid response of thermistors to temperature changes, these sensors are prone to sensitivity drift over time, particularly when exposed to high temperatures for extended periods. Furthermore, the harsh chemical environment within LiBs can degrade thermistor materials, potentially releasing harmful substances that compromise battery performance. The nonlinear resistance–temperature relationship of thermistors also necessitates complex calibration and compensation algorithms within the BMS, adding to integration challenges.

#### Pressure Sensors

**Fig. 3:**
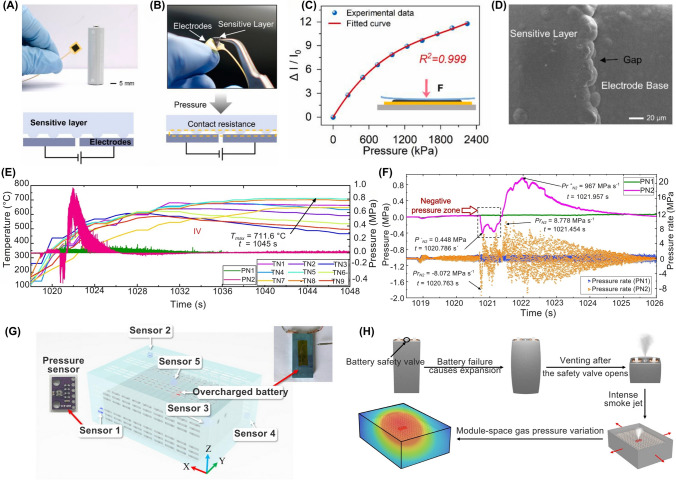
Schematic illustrating the integration of LiB with pressure sensors. **A** A thin-film piezoresistive pressure sensor with an operating mechanism designed for in situ measurements in LiB, **B** The structural details and components of the sensor, **C** Pressure-sensor current output correlation with exerted pressures, highlighting pressure sensitivity, **D** SEM image of sensor surface and gap. **E** Explosion pressure and cell temperature during Stage IV of the 1C overcharge test, demonstrating how the explosion pressure and cell temperature change in the cycle during this stage of the test, and **F** Pressure and pressure rate across the 1C overcharge test, showing pressure buildup rate in the cell during overcharge. **G** Locations of air-pressure sensors for LiB pressure monitoring, with respective coordinates, **H** Mechanism of air-pressure change within LiB module during battery TR. Panels reproduced with permission from **A-D**, ref. [65], Elsevier; **E, F**, ref. [53], Elsevier; **G, H**, ref. [17], Elsevier publishing

Pressure sensors are employed to measure alterations in the internal and mechanical pressure levels of the LiBs (Table 2). This parameter is particularly important in sealed battery systems, where excessive pressure buildup can lead to leakage or even explosions [17, 53, 64]. These sensors are commonly used in BMS for EVs and renewable energy systems to ensure safe and optimal battery operations [14, 64, 65]. The process of in situ mechanical pressure measurement within large-format LiBs poses significant difficulties owing to the harsh internal environment of the battery and interference created by the battery shell. To address these issues, Chen et al. designed a potential approach for in-situ measuring the mechanical pressure of electrode stacks, also called jelly rolls [65]. This innovative method used embedded thin-film flexible pressure sensors, encompassing a sandwich structure consisting of a sensitive layer, an electrode, and an encapsulation layer, as shown in Fig. 3A-D [65]. The pressure sensors were placed inside a prismatic power LiB, and their evolution characteristics were correlated to the spatial location, charging and discharging rates, external pressure, and electrode stack assembly. Their study revealed a direct correlation between the mechanical pressure generated by the volume expansion of the jelly roll and the lithiation and delithiation processes of graphite on the negative electrode. The reversible and irreversible pressures measured using this technology could provide insights into the changes in pressure during different charging and discharging rates, external pressure, and electrode stack assembly. This information on the mechanical state of the battery could optimize charge and discharge processes during LiB operation [65].

**Table 2:** Key characteristics of pressure sensors for safety monitoring in smart LiBs

Sensor type	Sensitivity	Accuracy	Durability (thermal/Mech.)	Cost	Response time	LiB cell type/Cathode chem	Integration complexity	Power consumption	Typical applications	Refs
SP40 Pressure	Detects SoC- and temperature-induced pressure shifts (~ 3 − 10 kPa range)	N/A	High (stable over 1100 + cycles)	Low to moderate (commercial component)	Moderate (real-time capable)	Prismatic (PHEV2), NMC111	Moderate (glued onto custom-built cell lid)	N/A	Internal gas pressure monitoring for SoC/SoH estimation, thermal diagnostics	[15]
Air-Pressure Sensor	Detects pressure rise as low as 0.1 hPa in 427 s	± 0.06 hPa	High (robust, long-term reliable)	Low	Fast (detects within 2–5 s post-venting)	Prismatic, LFP	Low (non-intrusive module-space setup)	N/A	Early warning of TR in battery modules	[17]
Piezoelectric Pressure Sensors	High (shock pressure up to 0.997 MPa)	N/A	High (survives explosive force)	Moderate	Fast	Large-format ouch, NCM811	High (explosion-proof setup)	N/A	Measurement of explosion pressure waves during TR	[19]
Flexible Thin-Film Piezoresistive Pressure Sensor	High (ΔI/I₀ increases with pressure; detects changes from ~ 400 − 1600 + kPa)	~ 1.7% resistance deviation after drying at 105 ℃; stable up to 150 days in electrolyte	High (chemically stable, robust under high C-rate cycling, ~ 120 μm thick)	Low	Fast (real-time, sub-second)	Prismatic cells, NCM	Moderate (sensor embedded between jelly roll and shell)	Low	Monitoring in situ internal mechanical pressure, safety diagnostics, and manufacturing quality checks	[65]
Operando Load Cell Pressure Sensor	High (detects irreversible volume changes on the order of ~ 10 s µm thickness change, ~ 100 PSI pressure shifts)	Not numerically specified, validated with dV/dQ and XPS data	High (mechanically robust, used over > 100 cycles)	Low–Moderate	Fast (real-time, cycle-resolved)	Pouch cells: NMC, LCO, and NCA	Moderate (requires enclosure and custom mechanical setup)	Low	Detecting SEI growth via irreversible expansion; performance ranking of Si-based cells	[66]

Recent studies have indicated that large-format LiB pouch cells, particularly those with NCM811 cathodes, which are commonly used in EVs, are highly prone to TR and can result in explosions under abusive conditions such as overcharging [53]. To address this issue, Shan et al. used pressure sensors to monitor the explosion behavior of these cells under varying charge and discharge current rates (C-rate: 0.5C, 1C, and 2C) [53]. Their approach utilized a TNT (trinitrotoluene) equivalent conversion approach based on shockwave pressure to measure the released energy and associated hazards. They observed that the overcharge C-rate significantly affected the thermal characteristics of the cells, with higher C-rates exacerbating the severity of the explosion. Moreover, they identified an evident negative pressure zone in the pressure curves, which suggested that the cells lacked a self-supplying oxygen system during the explosion. This absence of internal oxygen led to an explosion caused by an exothermic reaction between the electrolyte and the cathode material (Fig. 3E) [53]. Furthermore, they determined that the physical explosion originated from the rapid expansion of gases due to the temperature rise, whereas the chemical explosion was initiated by an exothermic reaction between the electrolyte and the cathode material (Fig. 3F) [53]. Their findings suggested the importance of safety management strategies for mitigating the risks of overcharging in large-format LiB pouch cells. This contributes to enhancing the charge and discharge cycles of battery packs and highlighting the significance of addressing the risks associated with utilizing NCM811 materials in LiB for EV applications.

Schmitt et al. developed a method for monitoring the internal pressure of large-format custom-built prismatic LiB cells by embedding miniaturized pressure sensors [15]. These sensors were embedded into the battery cells, mounted on circuit boards, and equipped with analog-to-digital conversion for signal processing. By placing a pressure sensor over a distinct opening on the top cover surface of each battery case, the internal pressure could be measured without causing premature battery failure or accelerating aging [15]. The study found that gas pressure increased irreversibly during long-term cycling, which correlated with cell capacity loss. This suggests that internal gas pressure could serve as an indicator of the battery's SoH. Analog-to-digital conversion in these sensors facilitated precise and reliable data collection, enabling continuous monitoring of battery conditions over extended cycling periods [15]. In a study by Song et al., air pressure sensors were employed to monitor variations in the interior air pressure within a prismatic LFP battery module (Fig. 3G) under TR conditions [17]. The battery module was partially sealed using steel plates and tape, and the internal pressure was measured during venting processes under overcharge and overheating scenarios. The sensors detected significant air pressure variations linked to the venting phases occurring during TR. The findings provided valuable insights into the battery's safety, suggesting that monitoring pressure changes could serve as an early warning signal for TR, potentially triggered by faults such as overcharging and overheating (Fig. 3H) [17]. Their findings also showed that the average time interval between the warning signal and the onset of battery TR was approximately 473 s [17]. This duration was estimated to be sufficient to implement corrective measures, thus enhancing the safety of LiB operations.

Pressure sensors in LiBs enable early warnings of TR, reducing the risk of catastrophic failure or explosions. However, the harsh internal environment of LiBs, characterized by exposure to corrosive electrolytes, repeated lattice expansion/contraction of electrodes, and significant temperature gradients, can degrade sensor materials (e.g., delamination of encapsulation layers). Such degradation may induce increased noise, calibration drift, or sensor failure, compromising the accuracy of the battery monitoring system over time. Integration with BMS is further complicated by the need for signal conditioning, such as analog-to-digital conversion [15], and spatial resolution limitations that may necessitate redundant sensor installations [17]. Moreover, studies have shown that pressure sensors alone may not effectively distinguish between physical failure modes (i.e., gas expansion) and chemical failure modes (i.e., electrolyte-cathode reactions) due to overlapping pressure signals. This overlap poses challenges in accurately identifying the specific causes of LiB failures based solely on pressure measurements [53].

#### Strain Sensors

During battery operation, inherent electrochemical processes driven by Li-ion migration between electrodes and electrolytes produce two critical mechanical effects: (i) reversible strain caused by cyclical lattice expansion/contraction, which generates progressive structural fatigue and component decoupling over repeated charge–discharge cycles [16, 67], and (ii) irreversible swelling from parasitic side reactions such as Li plating, gas evolution, and SEI layer growth. These degradation mechanisms collectively reduce capacity retention by deforming battery components and irreversibly trapping active Li-ions, accelerating energy density loss through mechanical aging and Li inventory depletion [16]. Strain sensors are integral in measuring the mechanical deformation, stress, and swelling of LiB packs following Hooke’s law for the momentum equation [68, 69]. These sensors are capable of detecting structural damage or deformation that may impact the performance and safety of the battery (Table 3) [16, 18, 70]. They are essential for assessing the structural integrity of LiBs, as excessive strain can result in physical damage or even rupture. By monitoring the strain levels, these sensors enable the early warning of any mechanical stress that may compromise the safety and performance of LiBs. However, correlating mechanical strain with electrochemical performance, such technologies enhance diagnostic precision, support adaptive battery management, and pave the way for their application for performance optimization.

**Table 3:** Key characteristics of strain sensors for safety monitoring in smart LiBs

Sensor type	Sensitivity	Accuracy	Durability (thermal/Mech.)	Cost	Response time	LiB cell type/Cathode chem	Integration complexity	Power consumption	Typical applications	References
Free-standing microfiber strain sensor (piezoresistive)	High (Gauge Factor ≈ 9; detection limit = 0.00005 strain; ≈1 µm displacement)	High (SNR = 5 at 0.005% strain)	High (> 10,000 strain cycles; robust at 26 ℃)	Low (low material complexity; scalable wet-spinning method)	Fast (~ 11 ms stretch, ~ 34 ms relax)	Pouch cell NMC111	Moderate (requires sensor placement and alignment)	Low (~ 50 mV test voltage)	Real-time in situ battery thickness/expansion monitoring during cycling	[16]
Resistance strain gauge (BYM530-3BB(11)N4-B3D)	Gauge Factor = 2.04; 5 mV/µε (bridge voltage = 2 V)	High (temperature-compensated, ± 1% error)	High (> 10 pre-cycles; operates at -30 ℃ to 80 ℃; withstands 20,000 µε strain)	Low (commercial, simple materials)	Fast (100 Hz sampling rate)	Cylindrical 18,650 cells, NMC	Moderate (requires surface polishing, adhesive bonding, and curing)	Low (Wheatstone bridge)	Real-time expansion monitoring, SoC estimation, SEI formation detection	[71]
Strain gauge (HBM 1-LY11-6/120A)	High (max strain: 50,000 µm/m, 1000 µm/m per full cycle)	High (signal drift ≤ 30 µm/m at > 100,000 cycles)	High (> 100,000 cycles, stable with temperature variation)	Low (standard strain gauge)	N/A	Samsung 35E LiB cell, NMC	Moderate (requires precise placement and temperature control)	Low (minimal power required for signal processing)	Monitoring geometric changes (diameter/volume change) during charging/discharging cycles and aging	[67]

**Fig. 4:**
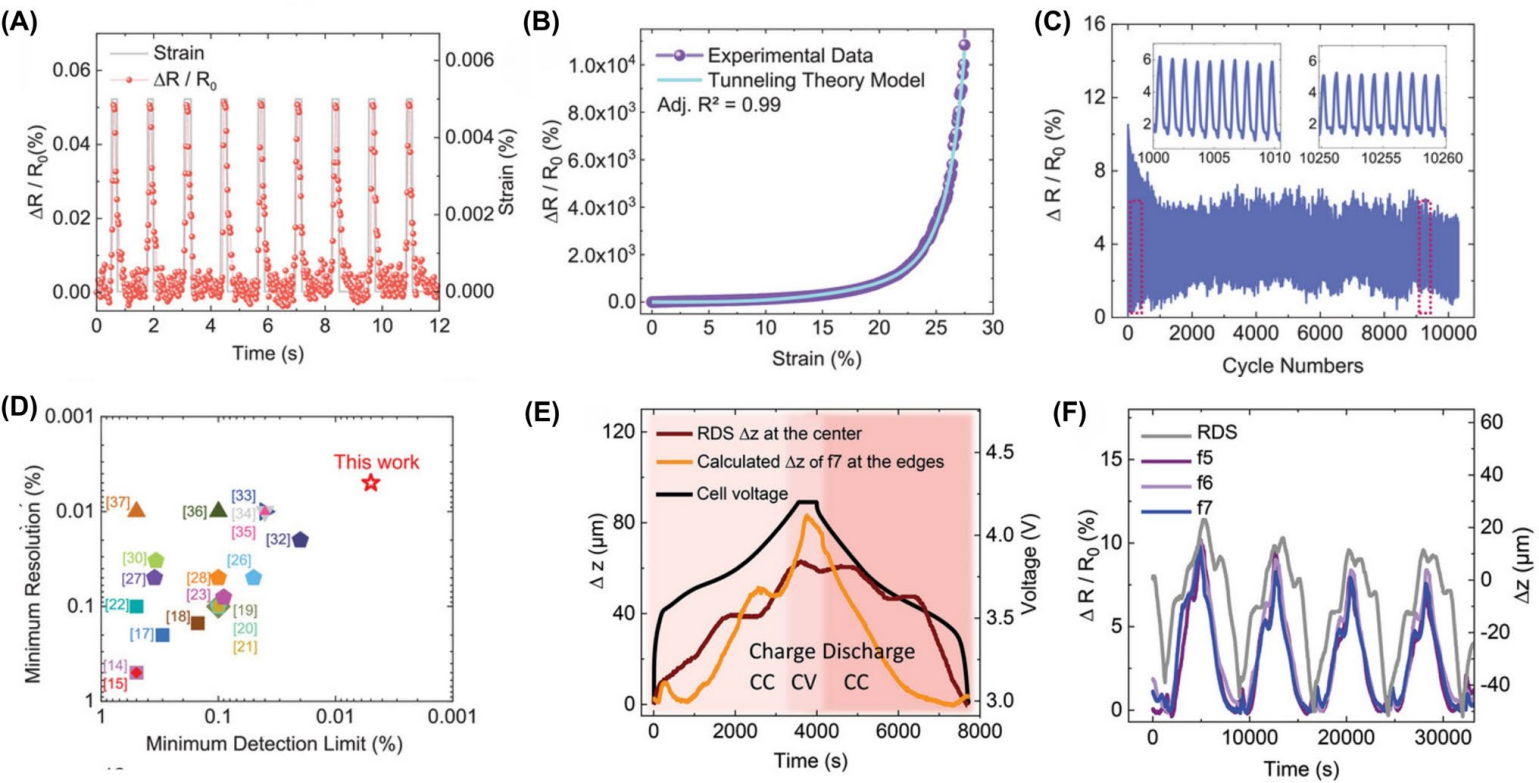
Performance and application of the microfiber strain sensor. **A** Stepwise resolution in low-strain regimes (0.005–0.025%), resolving 1 µm displacement. **B** Relative resistance change (ΔR/R_0_) versus strain, fitted to tunneling theory (adj. R^2^ = 0.99), showing linearity (GF = 9). **C** Durability over > 10,000 cycles at 1% strain. **D** Comparative analysis of detection limit (0.005%) and resolution against prior studies. **E** Real-time thickness change (Δz) of a LiB pouch cell during cycling, correlating sensor (edge) and reference (RDS, center) data. **F** Reproducible responses over four cycles. Panels reproduced with permission from **A-F**, ref. [16], Wiley–VCH

Conventional strain sensors often have limited sensitivity, which can hinder the detection of small variations in the mechanical properties of batteries. In addition, these sensors may struggle with spatial resolution, making it challenging to identify localized changes in the mechanical characteristics of LiBs. Recent advancements showed innovative approaches to detect reversible expansions from lattice changes during Li-ion intercalation and irreversible swelling caused by parasitic reactions, such as Li plating and SEI growth. For example, Graphene-based sensor arrays with ultrahigh sensitivity (gauge factor ≈150 μm) and distributed networks enabled precise mapping of localized strain hotspots, facilitating early detection of mechanical degradation [72]. Similarly, Nazari et al. developed a piezoresistive free-standing microfiber strain sensor for real-time LiB thickness monitoring, utilizing silver-coated glass microspheres in an ethylene–vinyl-acetate (EVA) matrix fabricated via wet-spinning [16]. The sensor achieved a record-low strain detection limit of 0.005% (1 µm displacement over 20 mm, Fig. 4A), linear response up to 14% strain (gauge factor *GF* = 9, Fig. 4B), and durability over > 10,000 cycles (Fig. 4C). Comparative analysis (Fig. 4D) demonstrated superiority in sensitivity over existing sensors, attributed to spherical core–shell fillers minimizing interparticle contacts and the elastomer’s viscoelasticity enabling rapid conductive path restoration [16]. Practical validation tracked real-time thickness changes (*Δz*) in LiB pouch cells during charge/discharge (Fig. 4E), resolving reversible expansion (≈62 µm) and subtle shrinkage (*Δε* =  − 0.05%). Such accuracy enabled early detection of irreversible swelling linked to aging or Li plating, which is critical for battery safety. Furthermore, reproducibility across four cycles (Fig. 4F) confirmed reliability for long-term LiB health diagnostics, addressing critical needs in energy storage systems [16].

The study by Nazari et al. demonstrated excellent sensor sensitivity and durability for accurately monitoring LiB thickness changes during cycling. However, its practical implementation faces several challenges. For example, while the EVA matrix and silver-coated microspheres exhibited resilience over 10,000 cycles, the weak van der Waals bonds between the EVA and silver shells can delaminate under repeated mechanical stress from electrode swelling, leading to signal drift or failure. The exposure to reactive electrolytes or high temperatures during TR may corrode the silver coatings or soften the EVA, altering piezoresistive properties. Moreover, temperature gradients during battery operation induce thermal expansion mismatches between the sensor and battery materials, potentially altering the piezoresistive response (*ΔR/R*_*0*_). While the study highlighted minimal hysteresis in cyclic tests, long-term field use may necessitate frequent recalibration due to the viscoelastic relaxation of the EVA matrix (Mullins effect). Although the sensor fabrication employed "green" solvents (anisole/acetone), the composite structure (EVA + glass-silver particles) complicates recycling. Integrated sensors cannot be easily separated from LiB cells, risking heavy metal contamination (e.g., silver leaching) during landfill disposal. Similarly, the study has not addressed recyclability, leaving a gap in sustainable lifecycle management. The analog resistance output (*ΔR/R*_*0*_) requires amplification, noise filtering, and analog-to-digital conversion to interface with digital BMS architectures. The sensor poses a rise in system cost and power consumption, particularly in large-format packs, requiring dense sensor arrays. Similarly, strain measurements are influenced by the thermal expansion of battery materials (e.g., electrode swelling during fast charging), necessitating advanced compensation algorithms lacking in current BMS designs.

#### Gas Sensors

TR, often triggered by abuse conditions such as overcharging, internal short circuits, or mechanical failures, leads to excessive heat generation, gas venting, and the potential for catastrophic failure [73, 74]. Various gases, such as hydrogen (H_2_), carbon monoxide (CO), carbon dioxide (CO_2_), and volatile organic compounds (VOCs), particularly hydrocarbons (C_x_H_y_), are usually emitted due to the decomposition of the battery’s organic electrolyte solvents and the cathode-electrolyte interactions under thermal stress (Table 4) [74–77]. It has been reported that hydrocarbons detected during TR, such as unsaturated olefins (e.g., ethylene (C_2_H_4_), propene (C_3_H_6_), and acetylene (C₂H₂)) and saturated hydrocarbons (e.g., methane (CH_4_) and ethane (C_2_H_6_), and propane (C_3_H_8_)) are significant contributors to the composition of off-gases in LiBs. This composition is influenced by factors such as the SoC, chemistry, and cell configuration, which are more prominent in higher SoCs [78, 79]. Vivek and Garcia-Araez found that during the formation of the SEI on graphite electrodes, ethylene was the predominant gas that evolved [80]. They observed that the ethylene was quickly consumed at Li metal electrodes when these electrodes were not pretreated. The consumption of ethylene at the Li metal electrode was linked to its reaction, forming polyolefins like polyethylene through radical polymerization [80]. This reaction pathway, which does not generate gas, was previously overlooked and offers insights into the alternative mechanisms of SEI formation [80]. Furthermore, their study demonstrated that the reactivity of Li metal to ethylene is significantly higher than that of graphite, which could have important implications for designing degassing protocols and safety strategies in LiB systems [80].

**Table 4:** Key characteristics of gas sensors for safety monitoring in smart LiBs

Sensor type	Sensitivity	Accuracy	Durability (thermal/Mech.)	Cost	Response time	LiB cell type/Cathode chem	Integration complexity	Power consumption	Typical applications	References
MO_X_ gas Sensors	High (H_2_ detection < 300 ppm, VOCs and CO responsive)	High in detecting early failure gases (e.g., H_2_, VOCs, CO)	High (long-term tested, robust under cycling)	Low (< €10–20)	Fast (within 10 s of first venting)	Automotive pouch/prismatic NMC/LMO/Graphite/LTO	Low to Moderate (PCB-based integration)	Low	Early detection of electrolyte vapor, H₂ from electrolysis, vent gas before TR	[81]
3D-DIW Al_2_O_3_/CuO Gas Sensor	High (11 − 16% response to 100 ppm vapors)	Moderate-High (precise selectivity)	High (stable up to 600 °C, annealed)	Low–Moderate	~ 14–19 s response, > 15–25 s decay	Pouch NMC	Moderate (requires placement near battery cells)	Low	Early detection of electrolyte vapor leaks in LiBs	[82]
Gas Sensors (H₂, CO, CH₄)	High (detects gas before TR: H_2_ ~ 579s early)	High (≥ 50 ppm threshold, early detection vs. smoke and TR)	High (stable during overcharge and TR)	Low (commercial Winsen sensors)	Fast (≤ 30 s, H_2_ fastest at 631 s post-start)	Cylindrical, LFP (18,650, 26,650, 26,700)	Low–Moderate (near battery cell in sealed chamber)	Low	Early warning of overcharge-induced TR using gas-based detection (H₂ preferred)	[54]
MXene-SnO_2_ (composite H_2_ sensor)	High (response up to 79% at 2000 ppm H_2_)	High (R^2^ > 0.98 for fitted response curves)	High thermal stability, tested up to 400 ℃, stable over 30 days	Moderate	Fast (~ 11 s at 600 ppm H_2_)	LiB N/A	Moderate (requires 400 ℃, thin film deposition)	Low	H_2_ detection during thermal runaway in LiBs	[83]

**Fig. 5:**
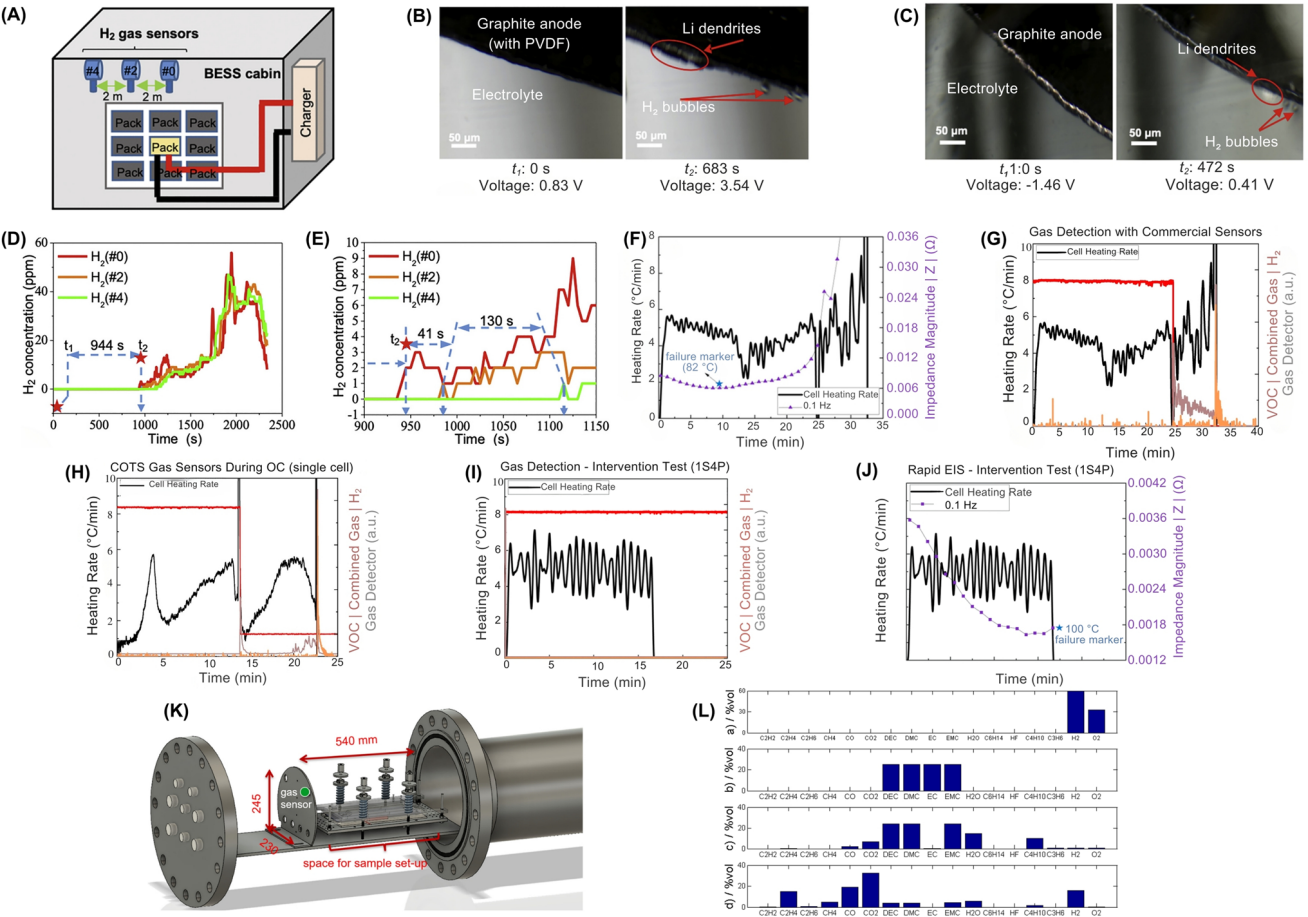
Performance of gas sensors in LiBs safety monitoring. **A** Schematic of battery energy storage system (BESS) cabin with three H_2_ gas sensors at varying distances, **B** The SEM images of graphite anode surface during LiB charging (with PVDF binder), **C** Similar to panel **B** but with a Li-metal electrode and graphite electrode (with PVDF binder), **D** H_2_ gas concentration variation curves of three sensors over 0–2500 s, **E** The detailed view of H_2_ gas concentration curves within 900–1150 s. **F** Rapid EIS of a single cell during OT test. Impedance at 0.1 Hz identified a failure marker (blue star) at 82 °C, **G** Gas sensor response during OT performed by VOC/Combined Gas/H_2_ sensors, **H** VOC/Combined Gas sensor response during OC test, **I** Rapid EIS test in a 1*s*4*p* pack during OC test, **J** Intervention test (1*s*4*p* OT). Deactivating heating at the EIS failure marker (~ 99 °C) prevented TR. **K** Location of the gas sensor (green) in the stainless-steel TR reactor setup, **L** Volumetric percentages of gases in four battery failure setups: electrolysis, electrolyte vapor, initial venting, and TR; The linear electrolyte components—DMC, DEC, and Ethyl methyl carbonate (CH_3_OCO_2_C_2_H_5_, EMC)—are equally present. Panels reproduced with permission from **A-E**, ref. [84], Cell Press; **F-J**, ref. [75] IOPSCIENCE; **K, L**, ref. [81], MDPI

The generation of CO is often accompanied by the release of H_2_, which has been identified as a precursor to TR, appearing even earlier than CO and CO_2_ in many cases [74, 84]. Gas sensors can be classified by their detection methods, including electrochemical gas sensors, cataluminescence gas sensors, infrared absorption-based gas sensors, resistive gas sensors, quartz crystal microbalance-based gas sensors, and optical fiber-based gas sensors [61]. In particular, chemo-resistive gas sensors have been employed to detect CO in these scenarios due to their high sensitivity, rapid response time, and cost-effectiveness [21, 75]. These sensors can detect minute amounts of gas, offering early warnings before other indicators, such as temperature or voltage fluctuations, become noticeable [21, 84]. For example, Jin et al. developed a gas sensing approach for early safety warning in LiBs by detecting H_2_ gas generated during the formation of micron-scale Li dendrites [84]. Their study demonstrated that the reaction between metallic Li and polymer binders (i.e., polyvinylidene difluoride (PVDF), styrene-butadiene rubber (SBR), and carboxymethylcellulose (CMC)) during dendrite formation produces H_2_ gas, which could be detected in real-time without modifying the commercial LiB cell structure. The method showed high sensitivity, capable of detecting dendrites as small as ~ 50 μm and capturing H_2_ gas at concentrations as low as 500 ppm. In overcharge experiments using an 8.8 kWh LFP battery pack, H_2_ was detected 639 s earlier than smoke and 769 s before fire onset (Fig. 5A-E). Further validation in real-world battery energy storage system (BESS) conditions showed that early detection by H_2_ sensors allowed intervention before TR, with neither smoke nor fire observed when charging was halted upon H_2_ detection. This practical approach enabled early safety warnings and provided a scalable, cost-effective enhancement to LiBs monitoring [84]. However, prolonged exposure to harsh battery environments, such as reactive electrolytes (e.g., hydrofluoric acid (HF) from LiPF_6_ decomposition) or high temperatures during TR, can corrode sensor components. Moreover, the integration of gas sensors within battery packs complicates LiB recycling, as mixed-material sensors resist separation, increasing landfill waste and the risk of heavy metal leaching. Similarly, gas dispersion within sealed battery modules may delay detection, as H_2_ released from localized dendrites took 990 s to reach external sensors. Such delays could escalate in tightly packed battery modules, potentially leading to missed early warnings and increased safety risks.

Furthermore, the integration of gas sensors with other diagnostic technologies, such as EIS, can significantly enhance the predictive capabilities of BMS systems [21, 75]. Torres-Castro et al. demonstrated this integration by using rapid EIS with commercial gas sensors (i.e., VOCs, combined VOCs/CO_2_/H_2_, and H_2_) to monitor TR in LiBs single cells and multi-cell configurations (1*s*4*p*, 2*s*4*p*) [75]. They tested both overcharge (OC) and overtemperature (OT) conditions, finding that EIS could identify failure markers much earlier than traditional BMS monitoring methods (e.g., voltage/temperature sensors). For OT tests, rapid EIS at 0.1 Hz provided the much earlier warning times (*Δt*_warning time_) before TR, detecting failure markers at ~ 82 °C (single cell: 22.5 min, 1*s*4*p* pack: 29.2 min; Fig. 5F). VOC/Combined Gas sensors triggered during venting, offering shorter warnings (single cell: 7.1 min, 1*s*4*p*: 17.3 min; Fig. 5G), while H_2_ sensors activated near TR onset (*Δt*_warning time_ ≈ − 0.4 min). In the OC tests, gas sensors exhibited EIS: VOC sensors detected venting ~ 8.5 min before TR (single cell and 1*s*4*p*; Fig. 5H), while rapid EIS warnings were shorter (~ 7.4 min for single cells). Pack complexity reduced EIS sensitivity, with impedance signals diminished sensitivity in larger packs (Fig. 5I). Intervention findings demonstrated that deactivating heating/current upon EIS or gas sensor triggers (e.g., at 99 °C in 1*s*4*p* OT tests; Fig. 5J) successfully prevented TR [75]. Their study suggested that combining rapid EIS (superior for OT) and gas sensors (effective for OC) enhances early detection, though pack design and sensor placement critically influence reliability [75].

Moreover, the choice of sensor technology is influenced by the battery’s composition, SoC, and operating conditions. For example, CO, CO_2_, and ethylene are typical gases produced during TR, and their detection patterns vary depending on the specific materials used in the battery [21, 85]. This variability necessitates the development of customizable sensor setups that can account for these differences. In addition to resistive sensors, other sensor types, such as nondispersive infrared (NDIR) sensors, are also being explored for their robustness and ability to detect gases such as CO_2_ [81, 86]. Essl et al. investigated early failure detection in automotive LiBs before and during TR using gas sensors (Fig. 5K) [81]. The performance of commercially available gas sensors was evaluated across four failure scenarios: (i) unwanted electrolysis of voltage-bearing components, (ii) electrolyte vapor leakage, (iii) initial cell venting, and (iv) TR. Their findings demonstrated that gas sensors could detect key failures such as H_2_ from electrolysis, VOCs from electrolyte vapor, and TR-related gases (e.g., CO, CO_2_). However, detection efficacy varied significantly depending on the failure mode and sensor type (Fig. 5L). Their study also confirmed that gas sensors could detect CO_2_ and CO emissions from TR, which are indicative of catastrophic failure. However, sensor placement, sensitivity, and environmental conditions influenced the response time and detection accuracy. Multi-pixel metal-oxide (MOx) sensors, such as the Sensirion SGP30 and SGX MiCS-6814, emerged as promising candidates due to their high sensitivity, multi-gas detection capability, and ability to distinguish between different failure modes [81].

However, the harsh TR conditions, including pressure spikes (up to 3.2 bar) and corrosive gases such as HF, irreversibly damaged sensors in overcharge experiments, leaving them inoperative post-TR. Moreover, cross-sensitivity to non-battery-related gases (e.g., gasoline) further complicates their use, as false positives from ambient contaminants may trigger unnecessary BMS interventions, compromising system efficiency. The energy demands of continuously operating sensor arrays, particularly heated MO_X_ sensors, conflict with the efficiency goals of EVs. Also, integrating gas sensors into the existing BMS presents technical and logistical hurdles. While the study proposed event-detection algorithms (e.g., signal-to-noise ratio thresholds and multi-pixel signal differentiation), real-world deployment requires advanced machine learning (ML) models. These models are necessary to accurately distinguish between different failure modes, such as electrolysis and overheated cables, and to minimize false alarms. Implementing these solutions would involve considerable computational resources and firmware upgrades. In addition, spatial limitations further complicate its integration into BMS, as gas diffusion delays in large battery packs may hinder timely fault detection. In another study, Cai et al*.* presented a fault-detection method that utilized expansion force measurements. These measurements are instrumental in identifying abnormal increases in force, a common symptom of battery swelling. In addition, they employed a nondispersive infrared (NDIR) CO_2_ sensor to detect venting events, often indicative of LiB failure. Their findings revealed that detecting gases released from the TR of LiB using gas sensors is an effective approach for providing early safety warnings and can further enhance the capability of charge and discharge processes in LiB [76]. However, during TR events, ambient temperatures can exceed 100 °C, potentially destabilizing the thermal stability of NDIR sensors. NDIR sensors operate optimally within a narrow temperature range of − 20 to 60 °C [87]. Exceeding these temperatures can lead to inaccurate readings or even sensor failure. Moreover, effective integration with existing BMS necessitates sophisticated algorithms capable of accurately interpreting CO_2_ concentration data to assess battery health and predict failure modes, often requiring advanced data processing and ML models.

#### Acoustic Sensors

**Fig. 6:**
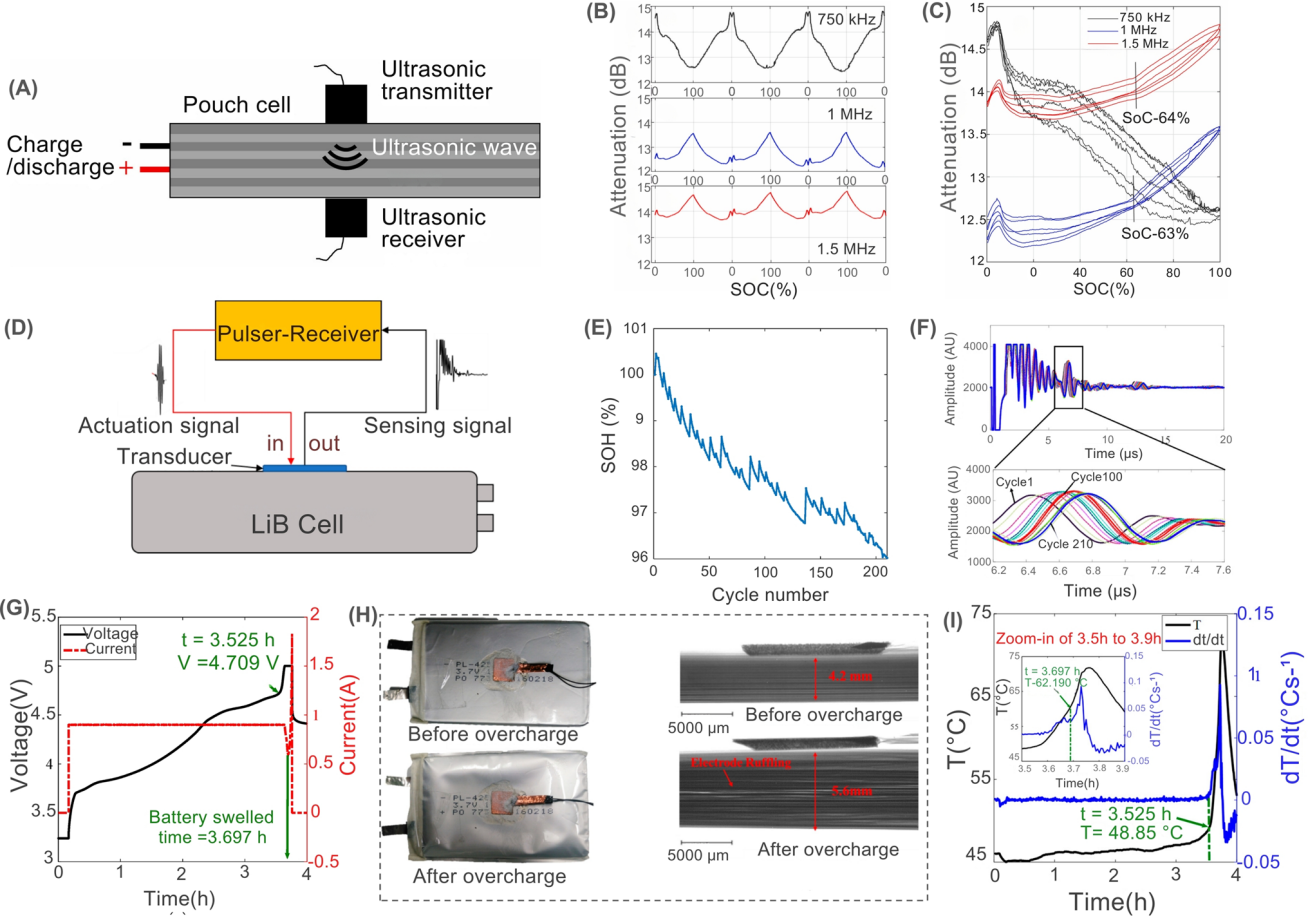
Performance of acoustic sensors for ultrasonic monitoring of SoC and SoH in LiBs. **A** Non-destructive ultrasonic testing principle on a LiB pouch cell for SoC monitoring, **B** Attenuation histories of ultrasonic waves at frequencies 750 kHz, 1 MHz, and 1.5 MHz, **C** Correlation between attenuation history and SoC for the three frequencies. **D** Pulse-echo mode ultrasonic transducers for battery SoH monitoring, **E** Battery SoH performance during battery cycling, **F** Signal amplitude over multiple battery cycles (Signal at cycle 1 was considered as the baseline signal. The deviation observed at cycle 210 is more prominent than at cycle 100.), Overcharge process tests: **G** Voltage and current performance, **H** Battery view and X-ray images of the LiB before and after overcharge tests, **I** Battery surface temperature and temperature change rate during the constant charging process of 0.5C (0.9 A) to 5 V. Panels reproduced with permission from **A-C**, ref. [94], Elsevier; **D-I**, ref. [95], MDPI

Acoustic sensors are used for LiB diagnosis to detect mechanical changes, such as electrode cracking, expansion or contraction of the electrodes, and electrolyte movement, by analyzing acoustic or ultrasonic signals (Table 5). These signals are influenced by SoC, SoH, temperature, and internal faults [88–92]. Acoustic methods, including acoustic emission (AE) and ultrasonic testing (UT), provide non-invasive, cost-effective monitoring with high spatial and temporal resolution [88]. For example, Robinson et al. used spatially resolved AE imaging to study electrode lithiation/delithiation at 36 distinct locations on the cell surface in a commercial LCO battery, revealing spatial irregularities in electrode expansion caused by current-collecting tabs [93]. Sun et al. demonstrated that multifrequency UT waves (750 kHz, 1 MHz, and 1.5 MHz) correlate linearly with the SoC of pouch LiB cells and capture phase transitions during cycling (Fig. 6A–C) [94]. The findings at the attenuation of 1.5 MHz revealed the distinct redox peaks observed at 3.46 and 3.64 V on the charge curve and two peaks at 3.40 and 3.59 V on the discharge curve, which indicated the occurrence of Li intercalation into the graphite anode and a phase transition in the NMC622 cathode, as essential processes for LiB operation and performance [94]. Similarly, Zhang et al. linked AE signal types (continuous vs. pulse) to SoH degradation, where continuous signals declined with cycling and pulse signals indicated aging [89]. Their findings revealed that the amplitude of the constant AE signal decreased with an increase in battery cycle count. Furthermore, the number of pulse-type AE signals gradually reduced during the initial cycles but exhibited a slow rise in the later cycles. Thus, a continuous AE signal could characterize performance degradation, while a pulse-type AE signal could be used for aging monitoring [89]. The study suggested a novel method for detecting the SoH of LiB using AE technology.

Furthermore, the electrochemical-acoustic time-of-flight (EAToF) technique facilitates real-time, non-invasive monitoring of LiBs by correlating acoustic wave behavior with the battery’s SoC and SoH [96]. Hsieh et al. developed a framework relating acoustic wave behavior, such as changes in sound speed, to variations in the density and modulus of battery materials, which change as a function of SoC and SoH. Their study, conducted on commercial LCO/graphite pouch cells and NCA/graphite 18,650 cells, showed that the acoustic measurements were strongly correlated with electrochemical performance. Their study showed that changes in acoustic signals, such as time-of-flight (ToF) shifts and signal attenuation, indicated the battery’s SoC and mechanical degradation over time. Their findings demonstrated that ToF decreases and signal intensity increases during charging, while specific ToF peaks shift more noticeably near 0% and 100% SoC due to phase transitions in the cathode material. In addition, their method could also detect early signs of degradation, such as diminished charge acceptance and mechanical relaxation, through subtle yet consistent changes in individual echo amplitudes across multiple cycles [96]. Moreover, EAToF identified formation effects in newly activated cells and could distinguish material and design differences between battery brands. The reported EAToF offered a low-cost, contactless, and universally applicable tool for in-operando LiBs diagnostics, uncovering physical insights that are typically inaccessible through conventional electrochemical methods [96].

It has been reported that the mechanical, thermal, and electrochemical instabilities of Ni-rich layered oxide cathode materials, such as LNO cathode, during delithiation induce significant shrinkage in unit-cell volume during charging in LiB. The shrinkage leads to uneven stress distribution, fractures in both primary and secondary particles, and side reactions between exposed surfaces and the electrolyte, which detrimentally impact capacity retention in LiB [91]. Schweidler et al. applied AE measurements as a non-destructive method for operando monitoring the mechanical degradation and structural changes of LNO electrodes at high SoC in LiBs [91]. Their study showed that significant acoustic activity was primarily measured during the initial charging phase, diminishing during the next discharge cycles. In the following cycles, acoustic activity was only detected during charging, showing a steady rise up to around 3.8 V. Their study suggested that the initial increase in acoustic activity could be associated with the potential formation of the cathode SEI and the further increase in acoustic activity from 3.9 to 4.3 V might be attributed to the growth or conversion of the initial SEI [91]. Wu et al. studied ultrasonic sensing through pulse-echo mode ultrasonic transducers with basic electronics for non-invasive health monitoring and detecting early signs of degradation and failure in LiBs (Fig. 6D) [95]. They analyzed two key ultrasonic features, ToF and peak amplitude (PA), during battery cycling and abusive overcharge scenarios with a battery initial capacity of 1.88 Ah [95]. The results showed that after 100 and 210 cycles, the remaining capacity was 97.69% and 96.02%, respectively, with TOF deviations more pronounced in cycle 210 (Fig. 6E, F). A strong negative correlation with SoH was observed (Spearman’s r > 0.94), and a linear regression yielded R^2^ = 0.949, confirming TOF as a highly reliable degradation indicator [95]. During overcharge tests (up to 5 V) (Fig. 6G), the study detected significant physical changes that were captured ultrasonically, such as gas-induced (i.e., CO_2_ and CH_4_) swelling and electrode distortion layers, confirmed by X-ray and thermal observations (Fig. 6H, I). Battery surface temperatures ranged from 45 to 50 °C, rising sharply beyond 4.709 V, reaching 62.19 °C at 3.697 h, with visible swelling occurring at this point (Fig. 6J). A key result was the development of a Mahalanobis distance**-**based health indicator that fused ultrasonic and temperature data. This fused indicator provided early failure warnings 0.872 h before physical swelling and 0.817 h earlier than temperature-based methods, offering critical time margins for intervention. Their method was validated using commercial LCO/graphite pouch cells, demonstrating effectiveness in both moderate cycling and high-risk abuse scenarios, making it a practical addition to BMS for enhancing LiB safety and predictive maintenance [95].

Despite the potential advantages, several limitations are associated with the acoustic sensors used for LiB fault monitoring. For example, contact-based ultrasonic sensors require direct coupling to the battery surface, which may loosen over cycles due to electrode swelling, reducing measurement accuracy. AE sensors are sensitive to external noise (e.g., cooling systems, vehicle motion), complicating long-term operando monitoring without frequent recalibration. Energy consumption for continuous operation of high-frequency UT systems (e.g., 1.5 MHz) also conflicts with sustainability goals. Furthermore, scaling sensor arrays for large battery packs increases material use and electronic waste, necessitating recyclable or bio-friendly alternatives. Spatial resolution limitations further complicate integration: dense sensor arrays improve fault localization but increase system complexity and cost. For example, Sun et al. achieved SoC correlation using multiple frequencies but noted that signal attenuation patterns vary with cell geometry, necessitating cell-specific calibration [94]. Compatibility with legacy BMS architectures is another hurdle, as most systems lack dedicated hardware for acoustic data acquisition. Furthermore, overlapping signals initiating from various battery components and processes complicate the detection of specific failure modes and accurate estimation of the overall SoH of the battery. This ambiguity limits fault diagnosis precision; for example, distinguishing Li plating from particle cracking requires frequency-domain analysis with high signal-to-noise ratios, which is challenging in dynamic operating conditions [91, 93].

**Table 5:** Key characteristics of acoustic sensors for safety monitoring in smart LiBs

Sensor type	Sensitivity	Accuracy	Durability (thermal/Mech.)	Cost	Response time	LiB cell type/Cathode chem	Integration complexity	Power consumption	Typical applications	References
Piezoelectric Acoustic Emission (AE) Sensor (VS-45H)	High (detects stress waves at 60–88 kHz and 0–150 kHz)	High (distinct signal patterns correlated with SoH)	High (tested up to 600 cycles with stability)	Moderate	Fast (~ μs to ms)	ICR18650-22P, LCO	Moderate (requires aluminum coupling and mounting)	Low	Real-time SoH estimation via stress wave signal from gas generation, cracking, SEI formation	[89]
Operando Acoustic Emission	High (detection of SEI formation and fracture events)	High (events clustered by frequency domain)	High (stable over multiple cycles, robust signal extraction)	Moderate	Fast	CR2032 Coin cells, LNO	Moderate (requires coupling and filtering system)	Low	Operando detection of SEI formation, phase transitions, and particle cracking in LNO cathodes	[91]
Piezoelectric Ultrasonic Transducer (5 MHz)	High (detects lithiation-induced density and dimensional changes)	High (ns-scale ToF resolution)	High (stable under pressure, non-invasive)	Moderate	Fast (6 μs scale; 12.8 ns resolution)	Samsung J5 battery, LCO	Moderate (requires surface scanning & signal processing)	Low	Spatially resolved electrode SoC/SoH monitoring, expansion/contraction detection	[93]
Phased-Array Ultrasonic Technology (PAUT)	High (multi-angle, high-resolution structural imaging)	High in micron level RMSE of the battery SoC estimation < 4.2%)	N/A	Moderate to High (Hundreds of thousands to millions of RMB)	Fast (every 5 s)	large-format aluminum shell ternary LiBs	Moderate (external probe setup)	N/A	In situ SoC estimation, structural fault detection, imaging during charge/discharge, and abuse conditions	[97]
Electrochemical-Acoustic Time-of-Flight (EAToF)	High (detects subtle density/modulus changes)	High (tracks SoC & SoH trends)	High (non-invasive, robust to mechanical stress)	Low (The sensor does not require modifications to the LiBs or any invasive procedures)	Fast (every 30 s))	Pouch and cylindrical (18,650), LCO, NCA	Low (no cell modification; single-point contact)	Low (The power consumption of EAToF sensors is low)	SoC/SoH monitoring, phase change detection, early failure/degradation warning, brand/manufacturing comparison	[96]
Ultrasonic sensing (TOF, PA)	High (detects physical changes like swelling and gas formation)	High (correlation with SoH > 0.94)	High (resistant to mechanical and thermal changes)	Low (using standard ultrasonic transducers and simple electronics)	Fast (real-time during charging/discharging)	Pouch cells, LCO	Low (simple setup with transducer and temperature sensor)	Low (using ultrasonic transducers)	SoH monitoring, degradation assessment, early failure indication	[95]
Multifrequency Ultrasonic Transducers (1–1.5 MHz longitudinal)	High (velocity change: ~ 1.3%; attenuation: 1–2 dB range)	High (R^2^ = 0.97–0.98 for SoC correlation)	High (repeatable for multiple cycles; stable setup with clamps)	Moderate	Fast	Pouch cell, NMC622	Moderate (requires clamping, alignment, signal processing)	Low	SoC and SoH estimation; phase transition detection; structural health monitoring	[94]

#### Magnetic Sensors

**Fig. 7:**
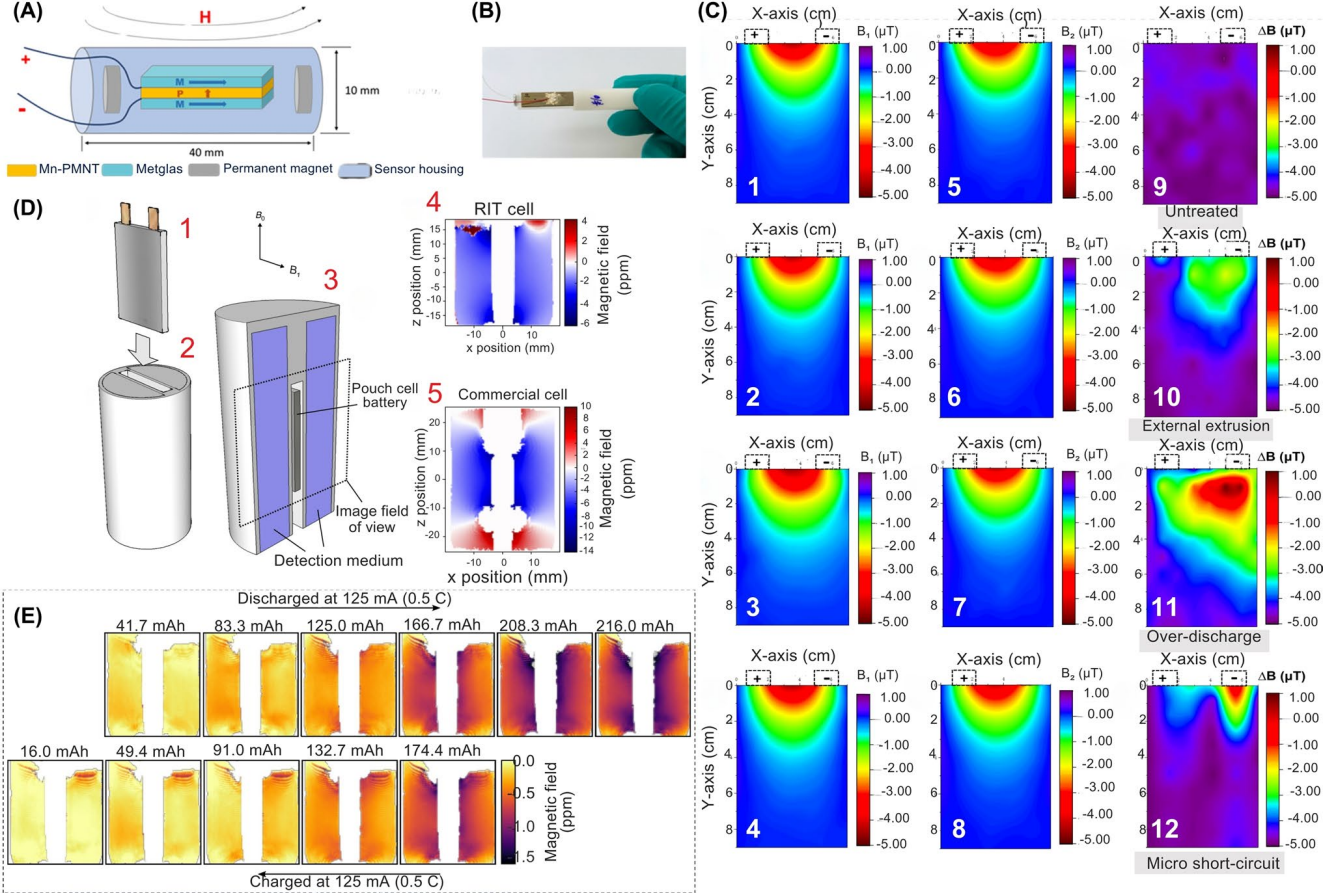
Schematic illustration of the magnetic sensors, structure, and performance in LiB monitoring. **A** Schematic of the ME sensor, depicting its structural design and components, **B** Actual photograph of the ME sensor, **C** Magnetic field distribution in healthy power batteries before (B1, parts 1–4) and after various treatments (B2, parts 5–8). Magnetic field variation (∆B) is illustrated for untreated (9), externally extruded (10), over-discharged (11), and micro short-circuited (12) samples. **D** Magnetic field map measurements for fully charged cells with placement and image orientation (1–3), and field maps measured for the cells (4–5), **E** Series of magnetic field maps at discharge and charge cycles, labeled according to cell discharge capacity. Magnetic field maps are cross-referenced with maps from fully charged cells using RIT cells. Increased cell susceptibility was observed during the discharge process. Panels reproduced with permission from **A-C**, ref. [102], MPDI; **D, E**, ref. [103], Springer Nature Publishing

Magnetic sensors have emerged as valuable tools for detecting magnetic fields induced by temperature variations, current fluctuations, and fault indicators in LiBs, enabling early failure detection and performance optimization ﻿(Table 6). These sensors enhance LiB functionality by manipulating electrolyte properties, electrode kinetics, and deposit morphology [98, 99]. For example, magnetic field imaging allows non-invasive visualization of internal current distributions [98]. Brauchle et al. utilized anisotropic magnetoresistive (AMR) sensors to map magnetic fields with an accuracy of 227 mA cm^−2^ and a local resolution of 4 mm^2^, enabling precise 2D current distribution analysis for SoH evaluations [98]. It has been reported that applying a magnetic field to lithium-ion batteries (LiBs) induces magnetization, forming numerous small magnetic dipoles within the battery. This magnetic alignment of particles significantly impacts ionic conductivity, promoting the accelerated flow and diffusion of ions [100]. Thanks to the article published in the *iScience* journal, Costa et al. conducted a comprehensive review on using magnetic fields in LiB components, including electrolytes, electrodes, and active materials [101]. The authors investigated the diverse mechanisms through which magnetic forces could interact with these components and examined their impact on electrochemical behavior. They suggested that effectively managing these forces and interactions can enhance the performance of LiB structures and facilitate the exploration of innovative approaches [101]. For example, Chen et al. constructed a magnetic sensor array that integrated a 16-channel high-performance magnetoelectric (ME) sensor (Fig. 7A, B) [102]. The noise equivalent magnetic (NEM) induction for each channel was determined to be within the range of 3–5 pT/Hz^1/2^ at a frequency of 10 Hz. This sensor array could non-destructively assess the SoH of LiB by monitoring the variation in the current supply during the charging cycle, distinguishing between healthy and degraded LiB cells (Fig. 7C) [102]. Although ME sensors are highly sensitive, they require stable piezoelectric substrates that may degrade under cyclic mechanical loads, posing a challenge to the long-term reliability of the sensors. Moreover, the ME array demonstrated pT-level sensitivity (3–5 pT/Hz^1/2^@10 Hz) but faced difficulties isolating LiB-specific signals in noisy environments.

In a recent study, it has been suggested that the measurement of tiny induced magnetic field variations within LiB cells could serve as an effective method for evaluating the degree of Li-integration into the electrode materials (Fig. 7D) [103]. The utilization of one-to-one mapping between the charge state and measured field map provides a rapid tool for determining the SoC of an unidentified cell (Fig. 7E). This approach is particularly valuable for cell types that lack available SoC information obtained through voltage measurements, particularly in cases where the cell integrity is compromised. Furthermore, this technique can potentially diagnose specific cell failures that may occur during the assembly process [103]. Zou et al. introduced a novel approach for gauging the temperature of LiB using an advanced magnetic nanoparticle thermometer (MNPT) [104]. They examined the influence of a direct current (DC) magnetic field on the temperature accuracy of the MNPT using MATLAB software and subsequently devised a novel model based on the ratio of the first and second harmonics. Through a series of simulations and experimental tests, they demonstrated that the improved MNPT could effectively determine the internal temperature of the battery. The findings showed that during the charging process, the battery temperature increased rapidly along with the voltage and current, gradually stabilizing upon full charge. While during discharge, the temperature peaked at 58.7 °C, slightly lagging behind the charging and discharging processes. The improved MNPT exhibited a temperature error < 0.5 °C compared to Pt100 sensors. These findings suggested a promising method for LiBs temperature monitoring in emerging EVs [104].

Magnetic sensors have garnered significant attention for various applications due to their numerous advantages, yet they face several challenges. For example, AMR sensors often use nickel–iron alloys (permalloy), while ME sensors incorporate lead-based piezoelectrics (e.g., PZT), raising concerns about hazardous waste disposal. Scaling these sensors for large-scale EV battery packs amplifies material use and end-of-life recycling challenges. Energy consumption is another concern as high-accuracy ME arrays and MNPTs require constant power for operation, conflicting with LiB efficiency goals. Furthermore, integrating magnetic sensors into conventional BMS poses technical hurdles, as most BMS lack dedicated hardware for magnetic data acquisition, necessitating additional signal conditioning circuits and analog-to-digital converters. Similarly, real-time processing of spatially resolved magnetic data (e.g., current distribution maps) demands significant computational resources, which may exceed the capabilities of legacy BMS architectures. Magnetic sensors are susceptible to ambient noise (e.g., EMI from nearby electronics or vehicle motors), which can obscure weak battery-generated signals. In addition, overlapping magnetic signatures from concurrent phenomena (e.g., Li plating vs. particle cracking) complicate fault diagnosis without advanced ML algorithms.

**Table 6:** Key characteristics of magnetic sensors for safety monitoring in smart LiBs

Sensor type	Sensitivity	Accuracy	Durability (thermal/Mech.)	Cost	Response time	LiB cell type/Cathode chem	Integration complexity	Power consumption	Typical applications	References
Anisotropic Magneto resistive (AMR)	High (detects down to 50 mA, SNR > 5, resolution 1.5 mAm^−1^)	High (measurement error ~ 0.0625%–2%)	High (long-term stable, no shielding needed)	Low (commercial components)	Fast (10 Hz sampling, real-time scan)	Pouch cells, NMC622	Moderate (2D scanning setup required	Low	Non-destructive current distribution imaging for SoH	[98]
Inside-out Magnetic Resonance Imaging (io-MRI) with SPRITE	High (detects magnetic field perturbations of ~ 100 ppm)	High (3D field mapping and DCF analysis)	High (solid-state electrolyte, stable signal capture)	High (research-grade MRI setup)	Slow (~ 3.7 min per scan	iPhone-4/8, LCO	High (requires custom MRI setup and image processing)	High	Non-invasive, real-time diagnostics of SoC, internal current, SEI growth, and defects	[20]
Tunneling magnetoresistance (TMR) Magnetic Sensor Array	High (detects magnetic anomalies caused by small ferrous objects at ≥ 50 cm)	High (improved SNR by 1.3 dB using PSD, enabling a 30% detection range increase)	High (compact system with robust passive sensing design)	Low (TMR sensors are low-cost and suitable for scalable deployment)	Fast (real-time detection with signal refresh every 2 s)	Commercial LiB, N/A	Moderate (requires calibration, signal processing, and sensor array integration)	Low (powered by 9V LiB, suitable for long-term use)	Portable magnetic object detection, smart security systems, magnetic fingerprinting	[105]
Magnetoelectric (ME) Sensor Array	High (~ 1.15 mV/nT, LOD: 5 pT/Hz^1/2^@10 Hz)	High (NEB: 3–5 pT/Hz^1/2^@10 Hz across 16 channels)	High (robust composite structure, stable scanning)	Moderate (lab scale)	Moderate (real-time scanning with interpolation)	Pouch cells, LFP	Moderate (array scanning and software processing required)	Low	Non-destructive detection of faults (e.g., over-discharge, extrusion, short-circuit) via magnetic field anomalies	[102]

### Sensors for Performance Optimization

Sensors for performance optimization focus on enhancing battery efficiency, extending lifespan, and improving overall LiBs performance. These sensors, such as EIS, are designed to monitor critical internal parameters, including the SoC, SoH, remaining useful life (RUL), and internal impedance, providing valuable insights into the battery’s condition [106, 107]. These sensors enable precise control of charging and discharging cycles, optimizing energy distribution and reducing the degradation rate. By continuously evaluating the battery’s internal state, performance optimization sensors facilitate more effective BMS, enhancing operational efficiency and reliability. This is especially crucial for applications where long life cycles and high energy density are required, ensuring the system performs optimally over time.

#### Optical-Based Sensors for Performance Optimization

Optical-based sensors exhibit advanced capabilities compared to non-optical-based physical sensors, allowing for rapid and simultaneous measurement of multiple parameters with high sensitivity [108, 109]. These sensors utilize light interactions with the various components of a battery, minimizing interference with battery performance and enabling low-invasive measurement of parameters such as electrolyte characteristics, temperature, pressure, and strain for safety enhancement, as well as SoC and SoH estimation in LiB [109]. Furthermore, optical-based sensors demonstrate durability and effectiveness in challenging environmental conditions, rendering them advantageous for battery monitoring [108, 109]. Optical-based sensors can detect changes in various optical signals related to the electrolytes and states of LiB cells, providing real-time information for timely maintenance and enhancing battery sustainability [110–112] (Table 7).

**Table 7:** Key characteristics of optical-based sensors for performance optimization in smart LiBs

Sensor type	Sensitivity	Accuracy	Durability (Thermal/Mech.)	Cost	Response time	LiB cell type/Cathode chemistry	Integration complexity	Power consumption	Typical applications	References
Rayleigh Scattering-Based Distributed Optical Fiber Sensor (DFOS)	High (detects temperature variation across millimetres-scale resolution)	High (± 0.01 ℃ resolution, < 0.1 ℃ repeatability)	High (chemical stability)	Moderate (lab-grade instrumentation)	Fast (real-time, ~ 23.5 Hz sampling)	Pouch cells (A5-size), NMC	Moderate (requires bonding sensor to cell surface)	Low (passive fiber)	Real-time distributed surface temperature mapping, hotspot detection, thermal management design	[113]
Evanescent Wave Fiber Optic Sensor (RFO & TFO)	High (detects real-time intensity changes linked to SoC)	High (correlates closely with capacity and Fe oxidation state)	High (chemically and thermally stable)	Moderate	Fast (real-time in operando)	Swagelok cells, LFP-P2	Moderate (requires embedding in cathode)	Low	SoC monitoring, electrode/electrolyte interaction analysis	[114]
Embedded Distributed Fiber Optic Sensor (DFOS) Rayleigh scattering-based (ε-DFOS and T-DFOS)	High (strain resolution: ± 30 με; temp: ± 0.6 ℃; SoC Coeff. = 0.96 με/%	High (SoC tracking ± 1.4 με error; high spatial resolution ~ 2.6 mm)	High (stable over 100 + cycles; minimal cell performance impact)	Moderate	Fast (real-time operando monitoring)	Pouch cell, NMC	Moderate-High (requires fiber embedding within electrodes)	Low (passive fiber)	Operando strain and temperature tracking, SEI formation monitoring, SoC/SoH estimation, cell aging diagnostics	[115]
FBG	High (detects strain changes of ~ 1 με; stress: 3–62 MPa)	High (strain → stress conversion via ΔλB shifts; birefringence for directionality)	High (stable under both liquid and solid electrolyte cycling)	Moderate	Fast (real-time operando tracking)	Swagelok cells, LTO:LPS:C	Moderate-High (embedded in electrodes or interfaces)	Low	Real-time internal chemo-mechanical stress tracking in LIBs and ASSBs	[1]
FBG	High (9.46—9.72 pm/°C depending on FBG position)	Moderate (average variation of 0.97–1.33 °C across three cells)	High (stable at operating temperatures, minimal mechanical strain)	Moderate (Requires specialized fiber optics and guide tube setup)	Moderate (measured in 1 Hz intervals)	N/A	Moderate (requires integration of FBG sensors with a 3D-printed mount and PTFE guide tube)	Low (passive system, no active components in FBG sensors)	Thermal management, individual cell temperature monitoring in LiB packs, battery safety, and diagnostics	[116]
FBG	9.08 pm/℃ (Top), 9.17 pm/℃ (Middle), 9.24 pm/℃ (Bottom)	± 0.12 ℃	High (mechanically stable and robust in chemical environments)	Moderate	Fast (~ 28.2% quicker rise time than thermocouples)	Cylindrical cells, LFP	High (requires precise integration into the battery)	Low	Real-time surface temperature monitoring, failure detection, and optimized thermal management in batteries	[50]
FBG	High (precise temperature measurement)	N/A	High (thermally stable, minimal impact on electrochemistry)	Moderate	Moderate (real-time capable)	Cylindrical 18,650 cells, NCA	High (requires careful integration into cell core)	Low	In situ core temperature monitoring	[117]
FBG	High	N/A	High (resistant to mechanical and chemical stresses)	Moderate	Moderate (real-time capable)	Cylindrical 18,650 cells, NCA cathode	High (requires integration with optical setup)	Low	In situ core temperature monitoring for high-energy cells during charging cycles	[118]
FBG	High (11 pm/℃)	N/A	High (resistant to mechanical and chemical stresses)	Moderate	Moderate (real-time capable)	Cylindrical 18,650, NCA	High (requires integration into cell core)	Low	In situ temperature monitoring, distributed thermal measurement within the cell	[119]
FBG	10.24 ± 0.10 pm/℃ (IC)	± 0.1℃ (Internal sensors)	High (chemical and mechanical resistance)	Moderate	Moderate (real-time capable)	pouch cell, LFP	High (integration inside the cell)	Low	In situ temperature monitoring of internal battery areas during cycling	[120]
FBG-Based Strain Sensor	Moderate-High (detects < 80 με strain and ~ 8 ℃ temp change)	RMSE: 3.50% (2 inputs), 1.02% (3 inputs: strain, temp., strain rate)	High (no impact on battery structure; stable over cycles)	Moderate	Fast (real-time; 1 Hz sampling)	pouch cell (8 Ah), LFP	Moderate (surface bonding with epoxy)	Low	SoC estimation via axial strain and temperature; EMI-immune monitoring; deep-learning compatible	[121]
Tilted Fiber Bragg Grating (TFBG) Sensor	Ultra-high (detects nanoscale changes in ionic concentration and SEI growth)	High (resonance shifts tightly correlate with dendrite formation and ion kinetics)	High (stable over multiple cycles	Moderate–high	Fast (real-time, sub-second)	Symmetrical coin cells, LFP	Moderate (requires precise electrode proximity)	Low	Operando monitoring of Li dendrite formation, SEI performance, electrolyte mass transport kinetics	[122]
Polymer Optical Fiber (POF) FBG Sensor	Very High (Temp: − 85 to − 91 pm/℃; Strain: 1.5 pm/με; Pressure: 29.5 pm/MPa)	High (clear SoC-SoH correlation via strain rate and wavelength shift)	High (reusable; stable over multiple cycles)	Moderate	Fast (real-time, sub-second)	Coin cells: LCO/Graphite, LCO/SiOx/C, LFP/LTO	Moderate (external epoxy mounting)	Low	SOC/SOH estimation, SEI/CEI formation detection, real-time strain and temp. profiling	[123]
Infrared Thermography (IR)	High (differential temperature)	± 2% (calibrated range)	High (robust against environmental influences)	Moderate	Fast	NMC (20 Ah), LFP (14 Ah), LTO (5 Ah)	High (requires IR camera and setup)	Low	Visual temperature mapping of the cell surface, functional for thermal management analysis	[44]
Infrared (IR) Thermography	High	± 0.31 ℃ (calibrated)	High (surface material is robust)	Moderate	Moderate	Cylindrical (26,650), LFP	Moderate (requires calibration)	Low	Non-invasive core temperature prediction based on surface temperature	[49]
NDIR CO_2_ Sensor	High (detects > 30,000 ppm CO_2_ post-venting)	± 10% of reading	High (15-year lifespan, stable drift < 0.15%/year)	Low (~ $8–$20)	Fast (~ 11 s to detect 10,000 ppm CO_2_)	Prismatic NMC cell (Sanyo 4.9 Ah)	Low (placed at vent outlet)	Low	Early warning of TR through CO_2_ detection in EV battery packs	[76]
Infrared (IR) Thermography	High (thermal sensitivity: 20 mK at 30 ℃)	High (max ΔT with thermocouples: ≤ 0.88 ℃)	High (non-contact, validated up to 10C discharge)	Moderate	Slow (10 Hz, 1 image)	Cylindrical (A123 26,650, 2.5 Ah, 3.3 V), LFP	Low–Moderate (non-invasive external setup)	Low	Real-time surface temperature mapping, heat generation quantification, BTMS evaluation	[124]
Operando Infrared (IR) Spectroscopy	High (detects EC decomposition, SEI formation, and thermal effects)	High (detects electrolyte decomposition and EC ring-opening)	High (stable across multiple thermal and electrochemical tests)	Moderate	Fast (real-time spectral tracking)	half-cells, LCO	Moderate (optical setup required)	Low	Real-time monitoring of electrolyte breakdown, SEI evolution, and thermal degradation onset	[125]
Fluorescence Microscopy (with CG-5N Dye)	High (detects Mn^2+^ down to sub-μM with linear fluorescence response)	High (quantified Mn^2+^ concentration and diffusion profile)	High (stable dye binding; non-destructive)	Moderate	Fast (2 s intervals used)	Coin and pouch cells, LMO	Moderate (requires optical setup, dye handling)	Low	Visualization of ion diffusion/dissolution, SEI effects, surface coating performance	[126]
DMA Fluorescence Microscopy	High (distinguishes active Li from byproducts; responds to nanoscale surface chemistry)	High (semi-quantitative correlation with electrochemical data)	High (no morphology alteration; chemically selective)	Low to moderate	Fast (visual signal appears immediately after reaction)	pouch cells, LFP	Low (simple surface treatment and fluorescence imaging)	Low	Visual diagnosis of dendrite growth, SEI quality, and electrolyte compatibility in Li-metal batteries	[127]
In Situ Ultraviolet–Visible Spectroscopy	High (LOD: 2.5 ppm; LOQ: 8.4 ppm; linear R^2^ = 0.98)	High (validated with ICP-OES, 82–101% recovery rate)	High (non-destructive, stable across SoC states)	Low–Moderate	Low (several hours–days of monitoring)	Coin cells, LMO	Low (uses optical cuvette setup, no electrode integration)	Low	Real-time Mn^2+^ dissolution tracking; evaluating cathode–electrolyte interface stability	[128]
UV–Vis Spectroscopy with PAR Probe	High (linear detection of Mn^2+^ from ~ 1–16 μmol/L; R^2^ = 0.997)	High (calibration curve; unaffected by cycling conditions)	High (stable during 3.5–4.5 V cycling; PAR/PS system verified)	Low–Moderate	Moderate (sample every ~ 1 min)	Coin cells, LMO	Moderate (custom cell with optical path and window)	Low	In situ Mn^2+^ dissolution tracking; cathode degradation analysis; SEI stability studies	[129]
Visible Raman Spectroscopy	High (resonance enhancement observed; bands at 485 and 595 cm^−1^)	High (band intensity ratios correlate with lithium content)	High (non-destructive, surface-sensitive)	Moderate–High	Fast (real-time spectra collection during cycling)	Swagelok cells, LCO	Moderate (requires optical access and transparent window)	N/A	In situ monitoring of Li de-intercalation, spatial chemical mapping, surface degradation studies	[130]
In-operando Raman Spectroscopy	High (detects Li_2_C_2_ band at 1850 cm^−1^ indicating Li plating onset)	High (temporal and spatial resolution; 1 μm^2^ spot size)	High (non-destructive, repeated cycling)	Moderate–High	Moderate (20 min intervals; real-time trends)	Commercial pouch cell (SLPB78216216H), LMO	High (custom optical cell, laser alignment)	N/A	Detecting lithium plating onset, SEI characterization, monitoring overcharge and degradation dynamics	[131]
Raman Spectroscopy	High (detects low ppm levels of TR gases)	High (validated with GC–MS)	High (suitable for high-temp TR conditions)	Moderate to High	Fast (real-time detection post safety valve opening)	Cylindrical cells (18,650), LCO	Moderate (requires gas flow path & optical access)	N/A	Early detection of TR, explosion risk analysis, gas diagnostics	[132]
In-operando Raman Spectroscopy	High spatial resolution (1 μm^2^); detects lithium acetylide band at ~ 1850 cm^−1^	Enables tracking of Li plating onset and growth in real-time with spectral confirmation	N/A	N/A	Moderate (~ 20 min intervals for Raman spectra collection)	Pouch-type commercial cells, NMC811	High (requires optical access, specialized operando cell design)	N/A	Real-time study of lithium plating and SEI evolution; safety and degradation mechanism analysis	[133]
Raman Spectroscopy	High spatial resolution (1 μm^2^)	High (detects lithium acetylide band (1850 cm^−1^) linked to plating onset)	N/A	High	Moderate (Spectrum captured every 20 min)	2032 SS Coin cells (Half-cells), NMC	Moderate–needs optical access	N/A	Real-time Li plating detection and intercalation monitoring	[134]

##### Fiber-optic Sensors

**Fig. 8:**
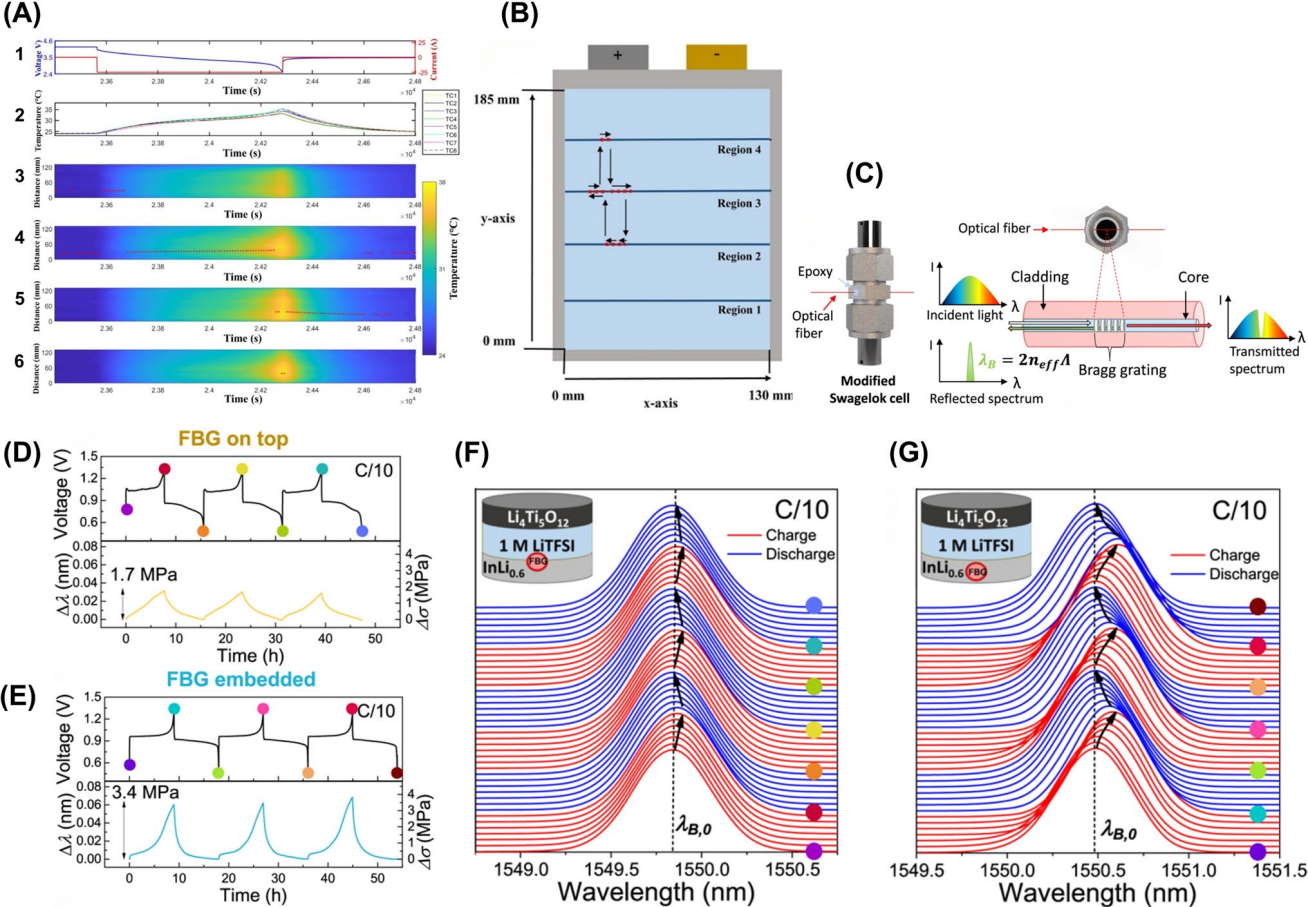
Schematic illustrating the performance of optical fiber sensors in LiB monitoring. **A** Results of 5C discharge at 25 °C ambient temperature, with cell surface temperature measured using DFOS and TC. The red dot indicates instantaneous max temperature by DFOS; (1) Evolution of current and voltage, (2) TC-measured temperature, (3), (4), (5), and (6) DFOS-measured temperatures and hotspots at regions 4, 3, 2, and 1 respectively, **B** Simplified graphical depiction of hotspot movement during 5C discharge at 25 °C ambient. **C** Integration of FBG into modified Swagelok cell and FBG sensor operational principle, **D** Time-resolved voltage (top) and Δλ, Δσ evolution (bottom) from FBG sensor in InLi_0.6_ | 1 M LiTFSI in DOL: DME | LTO cell with liquid electrolyte; FBG at anode/electrolyte interface, **E** Analogous plot for cell with FBG sensor embedded within InLi_x_ electrode. **F** Two-dimensional stack-view of reflected spectra by FBG sensor at the anode and electrolyte interface for cycles shown in **D, G** Analogous plot for cell with FBG sensor embedded within InLi_x_ electrode. Panels reproduced with permission from **A, B**, ref. [113], Elsevier; **C-G**, ref. [1], Springer Nature Publishing

Fiber-optic sensors enable real-time monitoring of LiB parameters such as temperature, strain, pressure, and ion concentration by analyzing spectral shifts in the transmitted, reflected, fluorescence, or absorption light [1, 61, 135]. These sensors provide critical advantages, including electrical passivity, resilience in harsh environments, compact size, and high bandwidth, enabling precise sensing in complex systems [136]. Their multiplexing capability allows dense, multi-point monitoring on a single fiber, minimizing wiring while enhancing spatial resolution. In large-scale LiB packs, this capability addresses the limitations of conventional module-level thermal monitoring, offering cell-level temperature data to improve safety and performance and prevent failures via advanced BMS [136]. For example, evanescent wave spectroscopy has shown consistent changes in the optical signal during Li-ion insertion and extraction, which enabled real-time SoH monitoring during LiBs operation [110]. Although commercial BMS utilizing optical fiber technology for sensing is still in the early stages of development, research is actively investigating their potential applications, particularly for temperature monitoring and internal state sensing within battery packs [137, 138]. However, there are currently no specific commercial BMS brands that extensively utilize optical fiber sensing and have gained widespread adoption. Recent reports have indicated the advent of innovative fiber-optic sensing technologies based on Rayleigh scattering [113]. The technology employed a distributed fiber-optic sensor (DFOS) and exhibited the potential to significantly enhance the measurement of the thermal behavior in NMC-LiB pouch cells. This technology allowed for comprehensive heat distribution monitoring across the cell surface, as well as the movement of the region with the highest temperature during operation (Fig. 8A, B) [113]. The findings derived from the DFOS indicated that the maximum in-plane temperature difference could exhibit a significant increase during 5 C discharge, reaching 307% higher than that obtained using conventional TC approaches [113].

Among fiber-optic sensors, FBG sensors are increasingly favored for LiBs monitoring due to their electromagnetic interference (EMI) immunity, compact size, and high sensitivity to strain and temperature variations [139, 140]. These attributes make them ideal for harsh environments, such as LiB packs, where EMI from high-current operations and thermal gradients can compromise conventional sensors, such as thermocouples or strain gauges. A key advantage of FBGs lies in their ability to simultaneously decouple mechanical strain and temperature through hybrid configurations [141]. This capability is vital for LiBs, where strain induced by charge–discharge cycles and thermal expansion/contraction must be distinguished to assess SoC and prevent mechanical degradation. In FBGs, decoupled sensing can be achieved through wavelength-shift analysis, where strain and temperature effects are computationally separated using sensitivity coefficients, enabling real-time monitoring of coupled thermo-mechanical behaviors [141]. Recent advancements in FBG-based strain sensing demonstrate their compatibility with LiB systems, where structural deformation correlates strongly with SoC and depth-of-discharge [18]. For example, a dual-FBG configuration comprising a functional grating (FBG I) and a thermally compensated grating (FBG II) was integrated into a commercial NCM pouch cell [18]. The system employed flexure hinges and symmetrical lever mechanisms to amplify strain sensitivity while minimizing transmission losses. The correlation between the FBG-strain sensor and SoC or depth-of-discharge indicated that strain rises with increasing SoC and decreases with increasing depth-of-discharge [18]. In another study, optical FBG sensors embedded within the coin and Swagelok cells, containing either a liquid or solid-state electrolyte, were capable of internal operando monitoring of Li-driven stress changes in InLi_x_ (indium-doped Li) and Li_x_Si (Li-silicon) electrodes during LiB cycling [1]. These data were then translated into stress data and correlated with the voltage profile (Fig. 8C-G) [1]. The findings showed that the reflected optical spectra captured during battery cycling revealed a progressive shift of the Bragg peak toward higher values during charging and toward lower values during discharging. This shift was attributed to local anisotropic lithium-driven stresses, suggesting the high mechanical reversibility of the system.

Furthermore, the integration of FBG sensors with advanced data acquisition systems and signal processing algorithms enabled the implementation of embedded monitoring systems. These can promptly warn operators or initiate automatic safety procedures upon the detection of potential failures in LiB cells. Rente et al. devised a real-time SoC estimation method for LiB employing an FBG-based sensor coupled with an ML algorithm [142]. Their approach utilized a dynamic time-warping (DTW) algorithm to optimize the fit using previously gathered data. The strain data extracted from the monitored optical signal served as input for the supervised DTW algorithm, enabling the prediction of the charging cycle. The study demonstrated strong agreement, achieving an accuracy rate of 2% and an SoC prediction resolution of 1%, suppressing traditional methods. Furthermore, the effectiveness of the method was confirmed through successful implementation in a ‘proof of concept’ scenario, particularly in a battery-powered train. Their results showed the potential of the real-time SoC estimator to enhanced safety measures within the rapidly expanding EVs industry [142]. In another study, Peng et al. examined the utilization of strain data from FBG sensors to estimate the SoC and SoH in the NCM pouch cell [143]. The strain data generated from the FBG sensors attached to the batteries was analyzed using a Kalman filtering (KF) model and an artificial neural network (ANN). The KF model applied real-time strain signal data to gauge the SoC based on a non-equivalent-circuit model, while the ANN model used strain information to estimate the SoC of different cycles [143]. The strain signals also played a crucial role in estimating the SoH of the battery, which indicates the battery’s capacity and overall health status over time. Their study showed that the FBG strain signal could serve as a standalone input to the models, accurately estimating the SoC without using traditional electrical measurement parameters [143]. This approach posed an advance in BMS, as it relies solely on the mechanical response of the battery. The strain data also provided insight into capacity degradation, which is crucial for predicting the lifespan and charge–discharge processes of LiB [143]. The study suggested that SoC and SoH estimations could be improved by integrating non-electrical parameters, enabling an alternative to conventional strain sensors that rely on voltage, current, and temperature data [143]. The development of ML models could further mitigate frequent sensor calibration to maintain their accuracy over time.

Similarly, the utilization of FBG sensors for real-time monitoring of internal pressure in LiB systems has been demonstrated to be an effective approach to early detecting gas-release events. With increasing pressure in a battery cell, the FBG sensors experience strain, inducing a shift in the Bragg wavelength (*λ*_*B*_). This shift can be accurately measured and correlated to the corresponding pressure changes through calibration within the LiB cell [109, 144]. In a study by Huang et al., a simultaneous monitoring method for temperature and pressure was developed using two FBG sensors [144]. The sensors, including the conventional single-mode fiber (SMF)-FBG and microstructure optical fiber (MOF)-FBG, were fused onto an 18-gauge needle in the same position. The needle was then carefully inserted into the LiB jelly roll through a pre-drilled hole on the negative electrode, rendering any strain generated by the jelly roll insignificant. After being sealed and filled with electrolytes, the battery experienced charge and discharge cycles while the temperature and pressure were continuously monitored. The FBG sensors exhibited varied sensitivities to pressure and temperature, enabling achieving crucial thermodynamic parameters [144]. The *λ*_*B*_ shift of the FBG sensor’s optical signal monitored the changes in voltage during the charging and discharging of two modified cells. Both cells exhibited closely overlapping voltage and *λ*_*B*_ shift profiles, indicating reliable sensor mounting and signal consistency. Internal temperature changes revealed four peaks during the initial charge, with only two persisting peaks in the following discharges. One of these peaks was associated with a structural change in the battery material, suggesting reversibility. While the peak with the highest amplitude during the first charge disappears in the following charges. The pressure profile reproduced this trend, indicating gas generation during charging. These findings suggested an irreversible phenomenon associated with SEI growth [144]. Their study suggested that potential thermal incidents could be effectively mitigated by configuring the heating and cooling system based on the observed signals.

Recent studies have shown that conventional fiber optics may not withstand the harsh conditions inside LiB cells, particularly under thermal stress. This is particularly crucial in the LiB cells where significant heat generation, about 500 ~ 800 °C, during operation may surpass the temperature range of the sensor, potentially leading to inaccurate measurements or damage to the sensor [145]. To address this issue, researchers have developed advanced fiber optics that maintain their integrity and capability over a wide temperature range, including temperatures suppressing normal operating conditions and reaching into the TR regime. These specially engineered fibers often integrate materials, such as sapphire femtosecond-laser-inscribed FBG and thermal-resistant polymers, significantly improving the thermal resistivity and stability of the sensors [145, 146]. Sapphire femtosecond-laser-inscribed FBG can withstand temperatures up to 1000 °C, far exceeding the necessities for safe LiB operation [145, 147]. This level of thermal resistance enables real-time temperature monitoring within the LiB, ensuring safety and aiding in BMS. In a study, Mei et al. tailored a hybrid FBG sensor by exposing an optical fiber to 800 nm light generated by a Ti: sapphire femtosecond laser and integration of an open-cavity Fabry–Perot interferometer (FPI). This exposure resulted in a periodic refractive index modulation along with the length of the fiber, leading to a light reflection at a specific *λ*_*B*_ resonance [145]. The sensor calibration results for the FBG *λ*_*B*_ shift exhibited a highly linear sensitivity of 99.9% to temperature, ranging from 25 to 600 °C, and insensitivity to a pressure below 2 MPa, ensuring fiber remains unbent at one end. Conversely, the FPI spectral dip *λ*_*FP*_ shift showed a highly linear sensitivity of about 99.9% to pressure changes, ranging from 0 to 2 MPa, while showing insensitivity to temperature changes within the range of 25–600 °C, less than 0.3 nm, equivalent to a minimal temperature sensitivity of 0.5 pm °C^−1^. The embedding of the FBG-FPI sensor, operando lab-on-fiber, into the cylindrical LFP cells, showed a minimal impact on the cell performance across various electrochemical charging conditions and during a lifetime test of 100 cycles at a 2C charging rate. Furthermore, the FBG-FPI sensor demonstrated a stable and reproducible correlation between the complex cell reactions and the optical signals, enabling temperature and pressure monitoring during TR conditions in cells [145].

Bonefacino et al. reported strain sensors by introducing polymer optical fiber-FBG (POF-FBG)-based sensors as a potential approach to track strain and temperature evolution in commercial LFP battery cells [123]. They used a silica-based FBG to monitor the strain and temperature changes in the cells during the charge and discharge processes. The first used fiber was a commercial germanium-doped silica single-mode G657.B optical fiber, while the second fiber was a POF made of ZEONEX®. The POF exhibited a two times higher sensitivity than silica single-mode optical fibers (SMF) for temperature measurements, with a negative coefficient of − 24.94 pm °C^−1^ compared to SMF of about 9.62 pm °C^−1^. Moreover, the POF also exhibited a higher strain sensitivity of 1.52 pm με^−1^ compared to SMF about 0.839 pm με^−1^ [123]. These findings showed the sensitivity of POF in the POF-FBG sensor for detecting thermal and strain data during LiB operation. Moreover, the study demonstrated a continuous increase in strain during the initial eight cycles, indicating irreversible cell expansion correlated with the Li-ion uptake/release amplitude and charge capacity retention. Additionally, the evolution of strain rate vs. SoC during each cycle indicated a gradual shift to higher rates of contraction/expansion, suggesting a transition from dominant expansion-inducing reactions to reversible cell reactions over parasitic ones, leading to cell stabilization [123].

Although fiber-optic sensors provide excellent resolution for LiBs monitoring, their adoption relies on advancement in durability, eco-friendly materials, and BMS compatibility. For example, POF-FBG sensors exhibit superior sensitivity but degrade faster than silica fibers due to polymer aging induced by cyclic thermal stresses. Similarly, FBG has limitations in detecting specific parasitic organic species related to chemical changes in electrolyte composition [148]. To overcome this limitation, alternative classes of optical sensors, including tilted-fiber Bragg grating sensors (TFBGs) [122, 149] and long-period fiber gratings (LPFGs) [150], have been developed. These sensors utilize evanescent waves to monitor changes in the refractive index of the surrounding electrolyte, which varies with salt concentration, enabling tracking of Li inventory and solvent concentration [148]. Furthermore, the manufacturing processes of ZEONEX®-based POFs and sapphire fibers require significant energy and pose recycling difficulties, contributing to end-of-life electronic waste. Chemical incompatibility also poses a critical concern, as prolonged exposure to electrolytes (e.g., LiPF₆) can corrode fiber coatings, leading to hazardous material leaching into the battery system. Moreover, the real-time processing of multi-parameter data (e.g., strain, temperature, pressure) can overwhelm BMS computational capabilities, necessitating extensive training datasets, sophisticated algorithms, and high-speed processing hardware to effectively correlate strain signals with SoC/SoH and accurately isolate individual parameters. Moreover, the ambient vibrations (e.g., in EVs) further distort strain measurements, necessitating noise-cancelation techniques absent in most existing sensor systems.

##### Fluorescence Spectroscopy

**Fig. 9:**
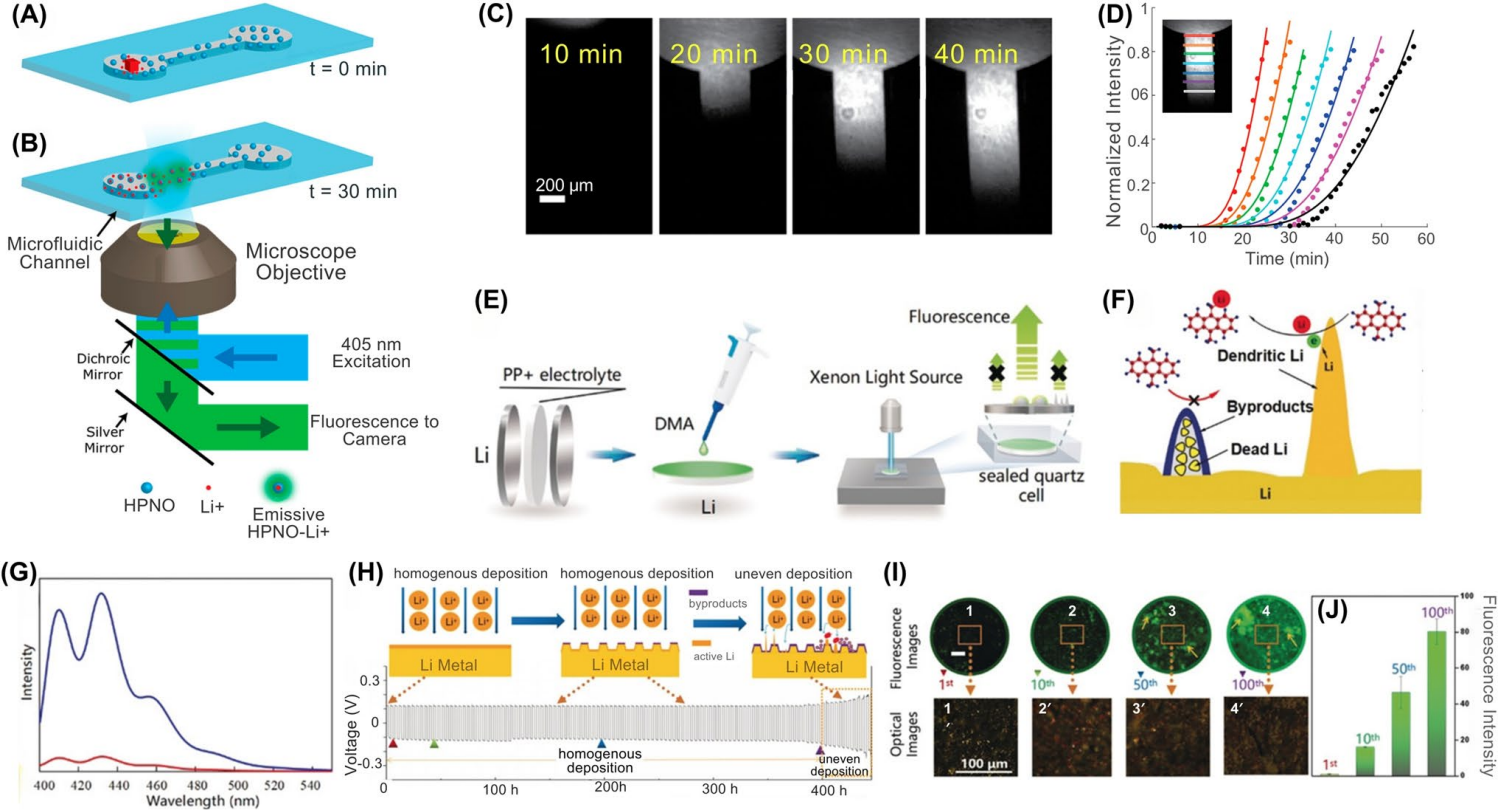
Schematic illustrating the integration of fluorescence spectroscopy for Li-ion characteristic monitoring in LiB during operation. **A** PDMS microfluidic channel with placed LiCl crystal (red cube) at one end, **B** Monitoring Li-ion motion within the channel using widefield fluorescence microscopy, **C** Widefield images of illuminated channels captured at different intervals, illustrating Li-ion diffusion, **D** Quantitative fluorescence intensity analysis at rectangular ROIs (inset), plotted over time. **E** DMA probing measurement illustration, depicting DMA reaction with components on Li surface, **F** Visualizing Li distribution on cycled Li metal surface through DMA probing test, **G** Emission spectra of 5 mg mL^−1^ DMA in dry TEGDME/DME (1:1) before (blue) and after (red) Li metal treatment. Samples diluted 1/100 in TEGDME/DME (1:1) for measurable intensity. DMA solution fluorescence intensity at 500 nm was reduced by a factor of 15 after reaction, **H** Li deposition in Li|Li cell with a voltage profile of symmetric Li|Li cell under 2.5 mA cm^−2^ current density and 2.5 mAh cm^−2^ area capacity, **I** Series of fluorescence images (1–4) of Li foils after 1, 10, 50, and 100 cycles. Arrows in (3) and (4) highlight byproduct-dominated areas. Images labeled (1′–4′) provide optical views of areas enclosed in orange rectangles in fluorescence images, **J** Mean of fluorescence intensity of cycled Li after 1, 10, 50, and 100 cycles. The excitation wavelength (λex) is 378 nm; the scale for fluorescence images is 100 μm. Panels reproduced with permission from **A-D**, ref. [152], ACS; **E-J**, ref. [127], Wiley Publishing

Fluorescence spectroscopy utilizes fluorescent dyes to monitor changes in Li-ion concentration and dendrite formation in the LiB electrolyte by analyzing wavelength shifts in emitted light [126, 151, 152]. During battery overcharging, the Li-ion concentration increases, leading to fluorescent spectroscopy detecting an increase in light emission [153]. This non-invasive, low-cost technique enables real-time tracking of lithiation/delithiation dynamics and electrolyte health under ambient conditions [152]. For example, in a study by Padilla et al., a fluorescent indicator of Li-ions, HPNO (2-(2-hydroxyphenyl) naphthoxazole, C_18_H_11_NO_2_), was synthesized (Fig. 9A, B) [152]. This indicator was employed for real-time monitoring of Li-ion movements via widefield fluorescence microscopy. The HPNO fluorophore could be excited using visible light, enabling quantitative determination of the Li-ion diffusion constant by applying Fick’s first law of diffusion for continuity equations [154, 155] within a microfluidic channel [152]. The study utilized PDMS (poly(dimethylsiloxane)), a polymer commonly used as a plasticized electrolyte in LiBs (Fig. 9C, D) [152]. The findings demonstrated real-time tracking of Li-ions with both temporal and spatial resolution, providing a novel, non-destructive approach for investigating lithiation and delithiation mechanisms in LiBs [152].

Cheng et al. introduced a novel fluorescent probing strategy that utilized DMA (9,10-dimethyl anthracene, C_16_H_14_) to visualize the distribution of active Li-metal on the anode surface of LiB cells (Fig. 9E) [127]. Their method could potentially distinguish between Li-dendrites and byproducts, both of which pose significant safety risks and lead to low coulombic efficiencies (Fig. 9F, G). Their study could potentially be employed for the selection of electrolytes and the predictive detection of uneven Li deposition on the anode (Fig. 9H-J) [127]. Their results showed that the electrolyte based on propylene carbonate (PC) exhibited significant polarization during plating/stripping, while the one based on vinylene carbonate (VC) showed minimal polarization and homogeneous Li deposition. DMA could detect dead Li coverage near the current collector in a pouch cell, indicating an uneven distribution of Li and its impact on electrolyte consumption and cycling performance [127].

Nevertheless, fluorescent dyes are prone to degradation under operational stress, which reduces the long-term reliability of fluorescence spectroscopy in LiBs monitoring [152, 156, 157]. For example, although HPNO can be excited by visible light, it can experience photobleaching during prolonged cycling, resulting in reduced emission intensity and diminished Li-ion sensitivity over time. Similarly, DMA’s reactivity with Li-metal byproducts (e.g., LiOH, Li_2_CO_3_) can alter its fluorescence properties, leading to signal drift in dendrite detection. Furthermore, synthetic dyes (e.g., HPNO and DMA) often involve toxic solvents (e.g., DMF (dimethylformamide)) and non-recyclable byproducts, increasing the carbon footprint of sensor production. Fluorescence sensors require external optical hardware (e.g., microscopes, light sources), which are incompatible with compact, electronics-focused BMS architectures. Similarly, real-time data processing demands high-speed cameras and ML algorithms (e.g., for dendrite detection), straining BMS computational resources. Calibration is another hurdle, as HPNO-based sensors require frequent recalibration to account for dye degradation. Moreover, while fluorescence microscopy provides µm-scale resolution [152, 158], its application is restricted to lab-scale setups (e.g., microfluidic channels) and cannot yet resolve sub-surface defects in commercial pouches or cylindrical LiBs cells.

##### Optical Absorption Spectroscopy

**Fig. 10:**
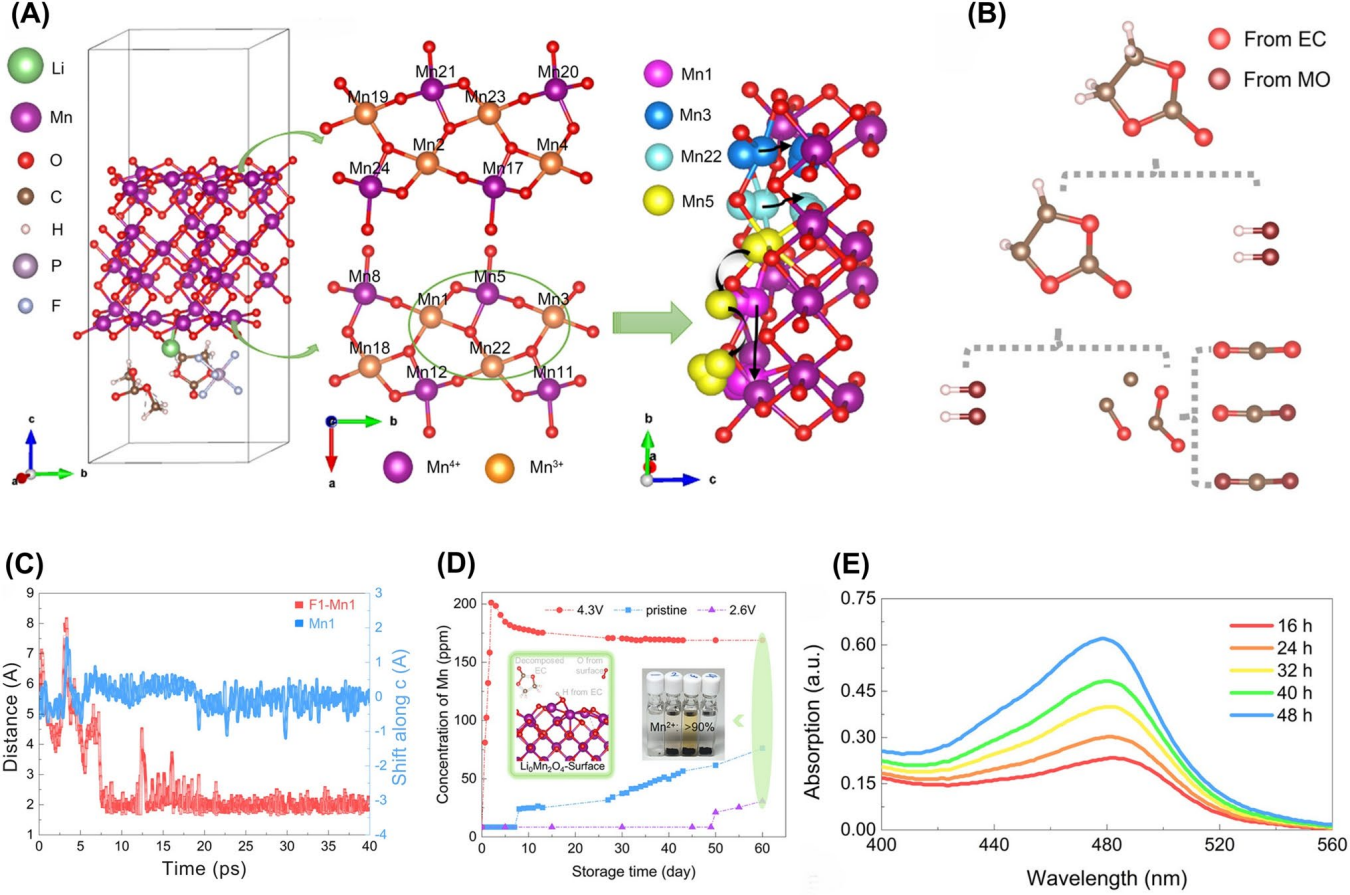
Schematic illustrating the concentration changes of dissolved Mn-ions in the liquid electrolyte from LMO at different SoC using a refined in situ UV–vis spectroscopy monitoring. **A** The model of the cathode-electrolyte interface features a MO’s (110) slab and electrolytes, including EC, DMC, and LiPF_6_. This model highlights the presence of Mn-ions in varying valence states at the interface layer, demonstrating the synergistic movement of Mn^4+^ (Mn5) and its surrounding Mn^3+^ (Mn1, Mn3, and Mn22). **B** The oxidative decomposition process of EC molecules is presented in a stepwise manner, **C** The interaction between F^–^ from LiPF_6_ and the surface Mn-ion (F^–^ exhibiting minimal impact on Mn dissolution). **D** A comparative analysis of the absorption peak intensity and the concentration of dissolved Mn over storage time for electrolyte/LMO-p, electrolyte/LMO-c, and electrolyte/LMO-d at 45 °C (The inset provides a visual representation of electrolyte cuvettes after 60 days of storage). **E** The in situ UV–vis spectra of electrolyte/LMO-c with varying storage times (16, 24, 32, 40, and 48 h) at 45 °C. Panels reproduced with permission from **A-E**, ref. [128], ACS Publishing

Optical absorption spectroscopy, also known as ultraviolet–visible (UV–vis) spectroscopy, is a non-destructive, sensitive method for detecting transition metals (TM) dissolution (e.g., Mn^2+^ from LiMn_2_O_4_ cathodes) and monitoring electrode degradation in LiBs [159–162]. The chemical reactions in LiBs can be monitored by measuring the changes in the UV–vis spectra over time, enabling the prediction of the remaining lifetime of the LiBs. It has been reported that the dissolution of TM cations from the cathode material into a LiBs electrolyte, such as Mn-ion from LMO, leads to capacity degradation in LiBs [129, 163–165]. Zhou et al. employed UV–vis spectroscopy in conjunction with the ab initio molecular dynamics (AIMD) simulations to monitor the changes in concentration of dissolved Mn-ion in electrolytes derived from LMO at varying SoC (Fig. 10A-C) [128]. AIMD simulations revealed a strong correlation between the Mn-ion dissolution process and the evolution of the surface structure, solvent decomposition, and Li salt. Their study showed that the maximum Mn-ions dissolution concentration occurred at the LMO charged state of 4.3 V, indicating that the valence of dissolved Mn-ions depends on the charge–discharge states (Fig. 10D) [128]. Similarly, their study suggested different perspectives on Mn-ion dissolution mechanisms, including the disproportionation reaction mechanism and the phase transition mechanism, indicating that the valence of dissolved Mn-ions could vary depending on the LiBs operating conditions. Through the analysis of UV–vis spectra, this technique effectively quantified dissolved Mn-ion concentrations for real-time monitoring, allowing for accurate determination of the actual Mn-ion dissolution content based on the intensity of the UV–vis peak at 483 nm (Fig. 10E). The recovery rate was observed to be nearly 1.0, with detection and quantification limits of 2.5 and 8.4 ppm, respectively [128].

While UV–vis spectroscopy provides critical insights into TM dissolution, its practical application requires robust miniaturization, eco-friendly materials, and advanced signal processing for BMS integration. For example, UV–vis systems require stable light sources and detectors, which risks lens fouling from electrolyte decomposition byproducts in a real-world deployment. Similarly, synthesizing calibration standards (e.g., Mn-ion solutions) involves toxic solvents (e.g., nitric acid), generating hazardous waste. Moreover, real-time data processing demands high-speed photodiode arrays and ML algorithms to deconvolve overlapping absorption peaks (e.g., Mn^2+^ vs. Co^3+^), straining BMS computational resources. UV–vis technique is also limited with turbid or light-scattering electrolytes (e.g., gel polymer electrolytes), leading to drop signal-to-noise ratios. Furthermore, UV–vis cannot detect non-UV-active species (e.g., Li^+^, PF_6_^−^) or distinguish between TM ions with similar absorption profiles (e.g., Ni^2+^ vs. Co^2+^) without complex chemometric models.

##### Raman Spectroscopy

Raman spectroscopy enables spatially resolved, in situ monitoring of any structural and molecular changes in LiBs components during charge and discharge cycles at a spatial resolution down to 1 μm^2^ [166]. This technology provides valuable insights into various aspects, such as electrolyte decomposition and phase transitions on cathode materials [130, 131, 167–169]. Raman spectroscopy offers the capability to promptly detect the presence of organic solvent vapor (OSV) upon activation of the safety valve, making it a promising tool for early warnings of TR and estimation of the risk of TR gas explosions [132]. Moreover, Raman spectroscopy could be employed for in-situ and confocal conditions [130, 170]. It has been reported that chemical heterogeneity across the LCO cathode surface could determine the presence of resonance enhancement for LCO materials when excited with a green laser during lithium de-intercalation [130]. This capability facilitates monitoring subtle changes in the LCO material’s structure and composition during battery operation, revealing the potential of Raman spectroscopy for spatially-resolved and in situ monitoring of LiBs. Similarly, in a study by Fang et al., in situ Raman mapping of LiB electrodes could allow for real-time tracking of SoC inhomogeneity on the single-particle level in LiBs [134]. This monitoring was developed based on industry-standard coin cells and a commercially available Raman spectrometer equipped with an electron-multiplying charge-coupled device (EMCCD) detector. The spectral evolution during charging in the in situ cells for LCO revealed bands located at 595 cm^−1^ for A_1g_ symmetry, arising from M–O stretching vibrations, and 485 cm^−1^ for E_g_ symmetry, arising from O–M–O bending vibrations, in the fully lithiated state. As the charging process progresses and positive electrode delithiation starts around 3.8 V, the Raman bands experience a downshift in frequency. Specifically, the A_1g_ bands shift from 592 to 530 cm^−1^, and the E_g_ bands shift from 488 to 459 cm^−1^. Their study suggested that the frequency and intensity of Raman peaks could serve as reliable indicators of local SoC with a spatial resolution of 1 mm [134]. Their study also tackled the inherent challenges of this approach, such as low sensitivity, potential light-induced alteration of battery materials, and the creation of an optical cell with uniform electric field distribution.

Furthermore, potential changes in the electronic structure of Li-intercalated graphite (Li_x_C_6_, LIG) during battery operation could be examined using X-ray Raman scattering (XRS) spectroscopy. This could facilitate the development of an in situ, as well as the development of a confocal-like method specifically designed to extract the XRS spectrum from the graphite electrode alone [170]. This method could provide valuable information about the chemical composition and structure of electrode material, potentially contributing to the development of safe LiB operation. It has been reported that carbonaceous additives (e.g., conductive carbon black) generate significant fluorescence that can obscure Raman signals unless mitigated by Kerr-gated systems [171]. Neale et al. developed a highly sensitive diagnostic tool, operando electrochemical Kerr-gated Raman spectroscopy, to accurately monitor the Li inventory in the graphitic carbon electrode of LiB to measure cell aging [171]. The application of the Kerr gate has been observed to suppress fluorescence emission signals, facilitating the measurement of Raman graphitic bands of highly lithiated graphite within the range of 0.5 ≤ x ≤ 1 for LIG. Initial observations indicated a broad graphitic band centered at 1590 cm^–1^ for Li_0.5_C_6_. However, upon further lithiation to LiC_6_, the band exhibited a linear shift to approximately 1564 cm^–1^. This shift provided a sensitive diagnostic tool for examining high SoC within graphitic carbon-based negative electrodes. This finding could hold significant implications for the development of more efficient and reliable LiB [171].

**Fig. 11:**
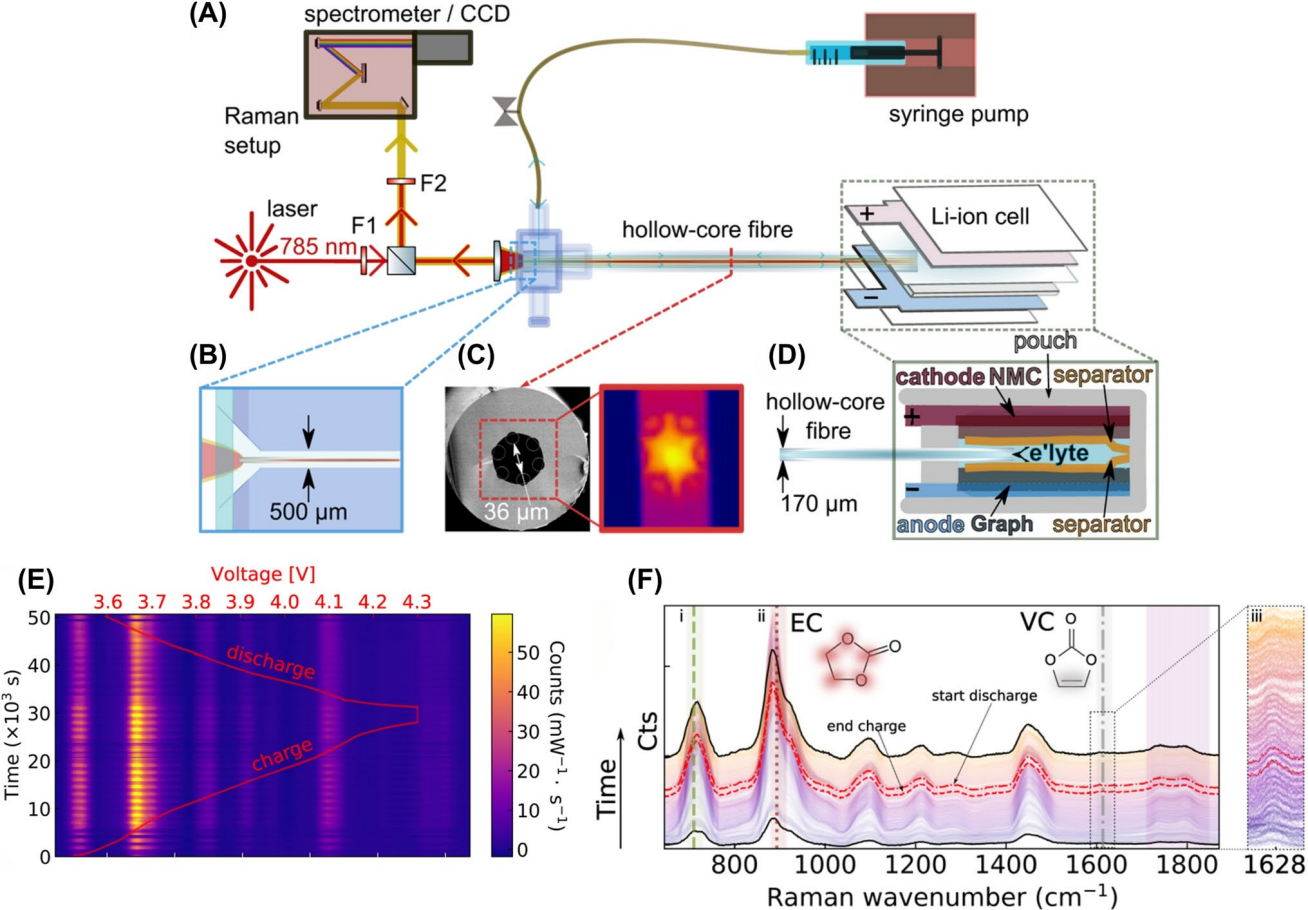
Schematic illustrating the integration of operando Raman spectroscopy for electrolyte monitoring in LiB. **A** The continuous-wave laser light (785 nm) filtered and directed into the core of a single-ring HC-fiber, **B** single-ring HC-fiber connected to a syringe pump for on-demand sampling or infusion, **C** SEM image of the HC fiber, which has an outer diameter of 174 µm and a core diameter of 36 µm, as measured between the inner capillaries. The accompanying image displays the Raman signal as detected by the charge-coupled device (CCD) camera of the spectrometer. **D** Arrangement of the electrodes, separator, and the fiber probe within the LiB pouch cell. **E** Operando Raman spectroscopy during the formation cycle of an NMC811- graphite LiB pouch cell using an LP57 + 2 wt% VC electrolyte. The cell was charged galvanostatically to 4.3 V, maintained the potentiostate at 4.3 V, and then discharged. **F** Raman spectrum, underlining specific Raman modes of LiB electrolytes: (i) PF_6_ − anion, symmetric stretch (740 cm^−1^, green dashed line), (ii) EC, skeletal breathing mode (893 cm^−1^, dotted red line), and (iii) vinylene carbonate (1,2-epoxy-3-propenyl carbonate, VC), –HC = CH– (1628 cm^−1^, gray dash-dotted line). Panels reproduced with permission from **A-F**, ref. [133], Nature Portfolio Publishing

Despite the potential of Raman spectroscopy for spatially resolved and in situ analysis of LiB, it is mainly a surface-sensitive technique that can examine only the outermost layers of the electrode material to 1 μm^2^. This can pose certain limitations when attempting to study thick electrodes or gather information about the bulk properties of the material during Li de-intercalation [166, 172]. In addition, the laser employed in Raman spectroscopy can cause localized heating of the sample, which may damage the electrode material or electrolyte [173, 174]. This heating effect can potentially affect the Raman spectra, making obtaining reliable and reproducible results challenging. Therefore, for a complementary measurement of LiBs, it is essential to integrate Raman spectroscopy with additional diagnostic methods. For example, a study by Miele et al. introduced an operando Raman spectroscopy sensor facilitated by hollow-core (HC) fibers-optic. This method enabled the real-time monitoring of the chemical alterations occurring in liquid electrolytes during the operation of LiBs (Fig. 11A-D) [133]. The integrated sensors effectively detected changes in the electrolyte of the battery, which was composed of a commercially significant high-energy Ni-rich layered oxide cathode of NMC811 and a graphite anode. The study on the spectroscopy measurements revealed variations in the ratio of carbonate solvents and electrolyte additives, which were found to be directly dependent on the cell voltage to monitor the solvation dynamics of Li-ion, referred to as continuity equation [175, 176] (Fig. 11E, F) [133]. Operando Raman measurements were conducted using the HC fiber during cycle 7. The EC breathing mode exhibited stability throughout the cycle, while significant fluctuations were observed during formation-cycle measurements. Additionally, a new peak developed at the position of the vinylene C = C stretch mode, indicating the formation of vinylene species due to electrolyte oxidation. The findings confirmed that the significant EC fluctuations and increases in vinylene species were associated with electrochemical processes [133]. This innovative method contributed to understanding the degradation mechanisms prevalent in different LiB systems.

##### Infrared Spectroscopy

Infrared (IR) spectroscopy is a non-intrusive technology that utilizes the infrared region of the electromagnetic spectrum (approximately 780 nm to 1 mm) to measure the absorbance and reflectance of light by molecular bonds, particularly OH (hydroxyl group), CH (methyl group), and NH (amino group) bonds, which can provide valuable information into the chemical and molecular characteristics of the internal components of the LiBs during charging and discharging cycles [124, 177, 178]. IR spectroscopy has the capability to penetrate deeper into battery materials than visible light, allowing it to provide valuable information on the internal structure of batteries [44]. Giammichele et al. investigated the thermal and electrical performance of a commercial LFP cylindrical cell for thermal management in LiB for electric mobility applications [124]. They used IR thermography to quantitatively measure the heat generation in battery cells, which was then compared to the results obtained from TC probe measurements to assess its reliability. In addition to this, an electrical characterization of the LiB was performed, measuring various parameters such as the cell potential, open circuit potential, and the entropic heat coefficient, referred to energy equation [179], to the SoC. The thermal images revealed that during discharge, the battery temperature increased, and at lower C-rates, the battery’s temperature remained relatively moderate by the end of discharge, slightly above ambient temperature. The IR thermography results compared to TC measurement demonstrated similar behavior. The findings indicated that the reversible term significantly influenced total thermal power, and the measurement of heat generation using IR thermography was reliable [124].

In-situ and operando IR spectroscopy has also been used in various LiB to monitor the performance and investigate the thermal stability of electrolytes [125, 177, 180, 181]. Saqib et al. developed an innovative operando IR spectroscopy to monitor the real-time degradation of the LiPF_6_/EC/DEC electrolyte in the LCO/graphite cell [125]. The study provided valuable insights into the mechanism of EC thermal degradation. The findings indicated a uniform mechanism across all tested cells, suggesting a consistent decomposition reaction unaffected by electrode material or potential. The primary mechanism identified for electrolyte thermal degradation was the ring-opening of EC. Additionally, the study found that operating LCO half-cells at voltages exceeding 4.2 V led to a permanent loss in LiB capacity [125]. However, no visible degradation of the electrolyte was observed, indicating that degradation of the LCO electrode occurs primarily at high voltage. IR thermometry was also used to measure electrolyte temperature during heated tests. Similarly, despite the high voltage of 4.5 V, the IR spectrum remained stable, suggesting no significant oxidation events in the electrolyte solvent. However, operating cells at temperatures exceeding 70 °C resulted in SEI and electrolyte degradation, highlighting the importance of temperature control in LiB operation. Furthermore, a decrease in thermal stability was observed with an increase in salt concentration, indicating that the degradation process may be catalyzed by the degradation of LiPF6 [125]. These insights into the mechanism of electrolyte thermal stability and degradation suggested IR thermometry as a valuable tool for designing smart LiB with higher accuracy and safety. Vizintin et al. introduced an operando-attenuated reflectance infrared (ATR-IR) spectroscopy approach for in-operando monitoring of changes in IR intensity of the carbonyl bond during redox processes inside the organic cathode, PAQS (poly-(anthraquinonyl sulfide)), which provided insights into the electrochemical mechanism of the cathode material [174]. The ATR-IR spectroscopy was further suggested for potential monitoring of the electrode degradation processes and electrolyte stability during the electrochemical cycling [174]. Nevertheless, the polymer-based optical fibers used in ATR-IR probes may swell or delaminate under cyclic mechanical stress from electrode expansion, compromising measurement accuracy.

The interpretation of IR spectra is intricate due to factors such as diverse chemical species in LiBs, overlapping spectral peaks, and potential interference from background materials [109, 182, 183]. To address these challenges, careful design of data-processing models rooted in density functional theory (DFT), referred to as the Kohn–Sham energy equation [184], is essential. The real-time spectral analysis (e.g., deconvolving overlapping peaks) demands high-speed processors and ML algorithms, exceeding the computational capacity of legacy BMS. Furthermore, IR spectroscopy is limited to a specific spectral range, potentially missing important molecular vibrations or reactions outside this range. IR spectrometers rely on toxic materials (e.g., mercury in MCT photovoltaic detectors), and their disposal results in hazardous electronic waste. Similarly, the reliability of IR spectroscopy data depends on frequent calibration during thermal stress.

#### Electrochemical-based Sensors for Performance Optimization

Electrochemical sensors, also known as contactless sensors, are utilized to monitor electrochemical processes occurring in LiB [2]. These sensors gauge the battery voltage, current, and impedance, enabling real-time tracking of internal changes. They provide accurate estimates of the SoC and SoH of the LiBs and can also detect early signs of degradation or inefficiencies, facilitating timely maintenance actions to prolong the battery’s lifespan and optimize performance (Table 8) [13, 63, 185, 186].

**Table 8:** Key characteristics of electrochemical-based sensors for performance optimization in smart LiBs

Sensor type	Sensitivity	Accuracy	Durability (Thermal/Mech.)	Cost	Response time	LiB cell type/Cathode chemistry	Integration complexity	Power consumption	Typical applications	References
Amperometric Hydrogen Sensor	High (sensitive to trace hydrogen detection in the ppm range. The sensitivity improves with temperature variations between 243 to 323 K)	High (linear fit with good reproducibility, detection limit 3.1 ppm)	Moderate (operates in a range of 243 to 323 K, stable over long durations)	Low (efficient design using titanium foam)	Moderate (*T*_*90*_ ~ 92 s, but dependent on environmental conditions)	Half-cell general LiB systems, N/A	High (requires sandwich structure and electrolyte integration)	Low (amperometric detection without active power consumption)	Hydrogen safety monitoring, early detection of TR	[28]
Conductometric IC-MOF Sensor	High (good response to DMC vapor)	High (up to 0.02 mL precision)	High (stable after prolonged use)	N/A	Fast (2 s)	Pouch cells, NMC	Moderate (sensor integration)	Low (passive sensing)	Real-time electrolyte leakage monitoring	[187]
Conductometric Co/Pd-doped SnO_2_ Sensor	High (Detects DMC vapor at ppb level)	High (165% response to 500 ppb DMC)	High (stable under 100–300 ℃ temperature)	Low (affordable material costs)	Moderate (response time ~ 66 s, recovery ~ 240 s)	Pouch cells, N/A	Moderate (sensor fabrication and integration with MEMS process)	Low (power consumption due to resistive sensing)	LiB electrolyte leakage detection, early failure detection, battery health monitoring	[188]
Impedance Sensors (Battery Internal Temperature Sensor-based BMS; BITS-BMS)	High (monitors impedance, phase shift across multiple frequencies)	High (able to detect impedance variations across multiple frequencies, ± 0.5% for cell matching)	High (designed to operate continuously, even under thermal variations, monitoring up to 16 cells)	Moderate (affordable and efficient design with low power demand)	Fast (~ 22 s per cell for data collection)	Pouch cells, NMC	Moderate (requires integration with BMS for data collection and temperature regulation)	Low (6 V, 0.75 A DC, with low power requirements)	LiBs safety, real-time monitoring of internal temperature, cell matching, detecting over-discharge and over-charge, TR prevention	[107]
Impedance-based BMS (Battery Internal Sensor)	High (detects degradation from impedance spectroscopy at 17.80 Hz and 2.16 Hz within the frequency range of 0.02 Hz to 20 kHz)	High (Predicts RUL with high accuracy, R^2^ > 0.87)	Moderate (operates in temperatures of 25, 35, and 45 ℃)	Moderate (based on electrochemical impedance data and ML model)	N/A	Coin cells, LCO	Low (requires integration with existing BMS, real-time impedance data collection across a wide frequency range)	N/A	Battery health monitoring, capacity estimation, and RUL prediction for LiBs	[106]
Impedance Spectroscopy (215 Hz)	High (sensitive to temperature variation)	± 0.6 ℃ (compared to the thermocouple)	High (suitable for battery cycling)	Low	Moderate (measures impedance at set frequency)	Cylindrical (26,650), LFP	Moderate (requires impedance measurement setup)	Low	Temperature estimation of battery internal conditions during charging/discharging cycles	[48]
Potentiometric Entropy Measurement-based BMS	High (detects structural changes through entropy change during cycling)	High (accurate entropy profiling with phases transition at ~ 4.08 and ~ 4.17 V)	High (operates in 20–60 ℃ with robust structure)	Moderate (moderate cost due to entropy profiling and electrochemical tools)	N/A	Thin-film microscale batteries (TFBs) cells, LCO	Low (needs integration with cycling/charging systems and other diagnostic tools)	Low (low power required for non-destructive entropy measurement)	Battery degradation monitoring, SoH tracking, solid-state battery diagnostics	[189, 190]

##### Potentiometric Sensors

Potentiometric sensors are non-destructive and measure internal changes within LiBs, including alterations in electrode structure and the behavior of Li-ions in the electrolyte. This monitoring is essential for evaluating the SoC or SoH by measuring the potential difference between the two electrodes in the LiBs. Such measurements help prevent overcharging and over-discharging, thus ensuring optimal performance and longevity in smart LiBs [29, 191]. Studies have shown that the LiBs operation produces irreversible and reversible heat [189, 192]. The reversible heat is associated with the entropy coefficient (*ΔS*) that can reflect the ordering of Li-ions within the host lattice during the lithiation and delithiation processes [190]. In a study by Zhang et al., an enhanced potentiometric measurement was introduced to investigate *ΔS* during cycling as an in situ diagnostic tool [189]. This method was particularly effective in monitoring the structural changes of the LCO cathode in solid-state electrolyte (SSE) batteries, specifically for the degradation phenomena of thin-film microscale batteries (TFB) during galvanostatic cycling [189]. Their study employed LiPON (Li-phosphorus-oxynitride) electrolyte, with an electrochemical stability window of up to 5.5 V, to examine *ΔS* during overcharging-cycling and high-temperature degradation. The use of LiPON mitigated the potential impact of liquid electrolyte reactions with the cathode, such as electrolyte decomposition [189]. The study found that changes in the material structure of the cell monitored through *ΔS* profiles could be associated with the phase transitions occurring at specific voltage ranges. These transitions included the charge–discharge cycles at around open circuit potential (OCP) ~ 3.9 V and at about x = 0.55 in LCO for OCP between 4.08 and 4.17 V, corresponding to the order–disorder transition, as well as a monoclinic-hexagonal transition at the end of charging [189]. The study suggested that the continuous *ΔS* measurement could facilitate real-time *dV/dT* (a derivative of voltage/time) characterization and enable SoH battery monitoring, providing insights into diagnosing degradation mechanisms in SSE batteries [189].

However, potentiometric sensors pose limitations, such as a restricted dynamic range determined by the material used, which causes challenges in accurately measuring potential differences across a wide range. For example, the restricted voltage window of materials, such as LiPON to 5.5 V, may hinder accurate ΔS measurement during extreme overcharging, limiting fault detection in LiB cells. These sensors demand careful calibration to obtain accurate measurements, a process that can be challenging in practical applications. Moreover, electrodes are susceptible to fouling by contaminants during operation, leading to reduced sensitivity and accuracy. For example, while LiPON electrolyte mitigated liquid electrolyte interference, fouling from cathode degradation (e.g., Co dissolution) necessitates periodic recalibration. Moreover, the sensors’ sensitivity to other ions in the battery electrolyte may introduce interference, potentially impacting measurement accuracy. The real-time ΔS measurement demands high-speed processors for *dV/dT* analysis, exceeding the capacity of microcontroller-based BMS.

##### Amperometric Sensors

**Fig. 12:**
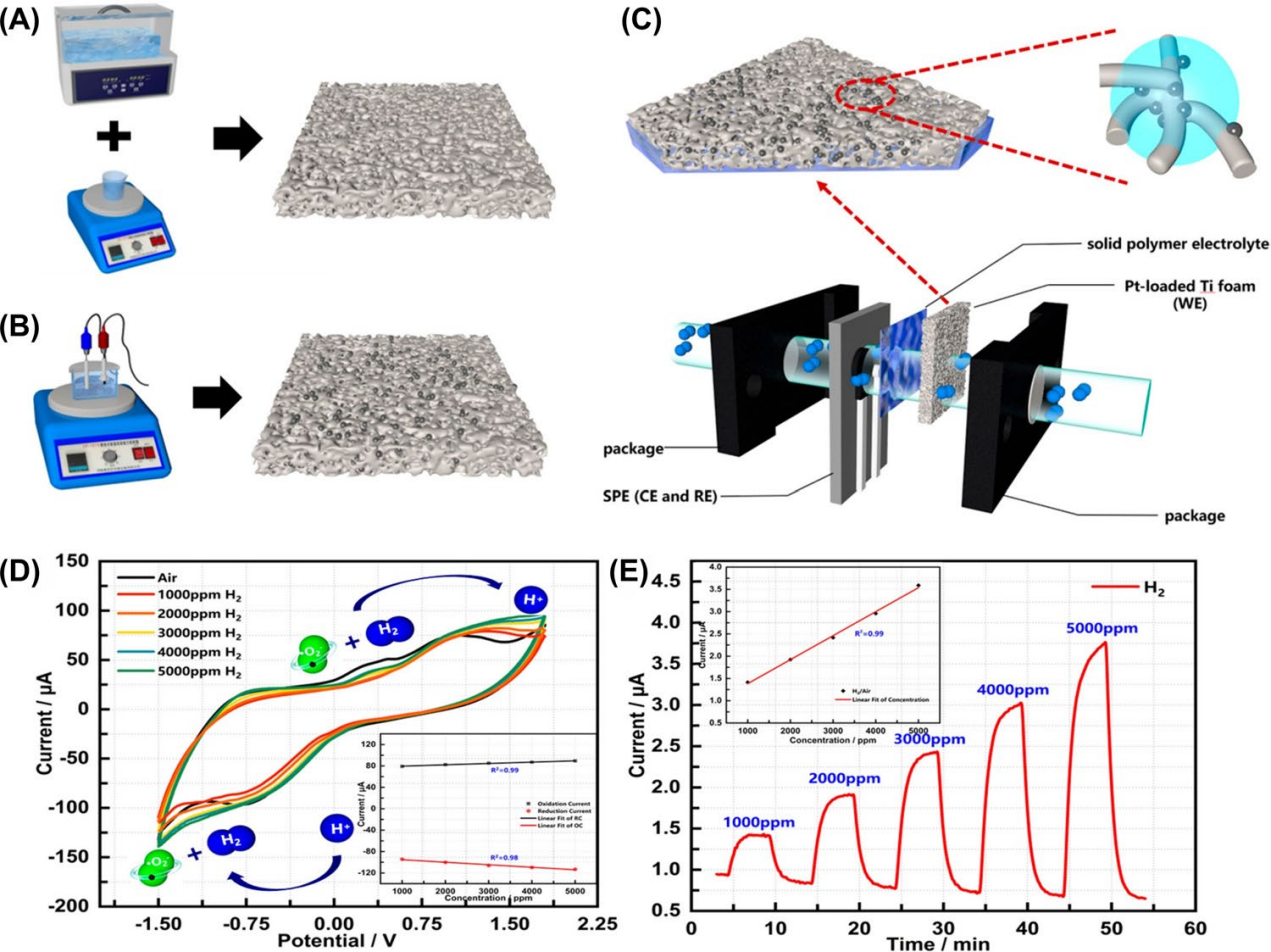
Schematic illustrating the fabrication and performance of amperometric H_2_ sensor for LiB monitoring. **A** Ti foam fabrication process for amperometric H_2_ sensor. **B** Electroplating step of H_2_ sensor. **C** Detailed H_2_ sensor structure. **D** Cyclic voltammetry (CV) performance of different H_2_ concentrations in aerobic conditions, followed by **E** Chronoamperometry at varying H_2_ concentrations. Insets in each graph display linear fits of chronoamperometry current to H_2_ concentration, highlighting direct proportionality between current and H_2_ concentration. Panels reproduced with permission from **A-E**, ref. [28]**,** ACS Publishing

Amperometric sensors, known as electrochemical VOC sensors, enable real-time, non-invasive detection of critical gases, such as H_2_, CO_2_, and other VOC gases, by measuring the current flow between two electrodes within LiBs [28, 76, 186]. For example, an amperometric H_2_ sensor with a distinctive ‘sandwich’ structure has shown promise for the safe and real-time monitoring of LiBs [28]. Gao et al. developed an amperometric H_2_ sensor employing an SPE (solid polymer electrolyte) and a Ti (titanium) foam electrode designed for safety detection in LiBs (Fig. 12A-C) [28]. The sensor facilitated a direct gas diffusion to the triphasic interface, enabling a current proportional to H_2_ gas concentration during LiBs damage stages (Fig. 12D, E). In addition, the SPE functioned as a selective barrier, ensuring exclusive detection of H_2_ gas. The sensor exhibited a linear response to H_2_ concentrations (0–5000 ppm) under diffusion-controlled conditions, demonstrating high sensitivity and rapid response time (Fig. 12E) [28]. Their study on amperometric H_2_ sensors revealed good sensitivity, long-term stability, low detection limit, and real-time and non-invasive monitoring, making it ideal for H_2_ detection in LiBs [28].

However, these sensors are susceptible to damage under high temperatures or severe conditions, such as environments with low humidity, depending on the physical properties of the electrode materials [76]. For example, prolonged exposure to high temperatures accelerates SPE dehydration, reducing ionic conductivity and sensor accuracy. Similarly, SPE often incorporates fluorinated polymers (e.g., PVDF) and platinum-group catalysts, which are energy-intensive in producing and releasing persistent pollutants if incinerated. Conventional BMS lack analog front-ends for low-current signals (nA–µA range), necessitating additional amplifiers and filters.

##### Conductometric Sensors

**Fig. 13:**
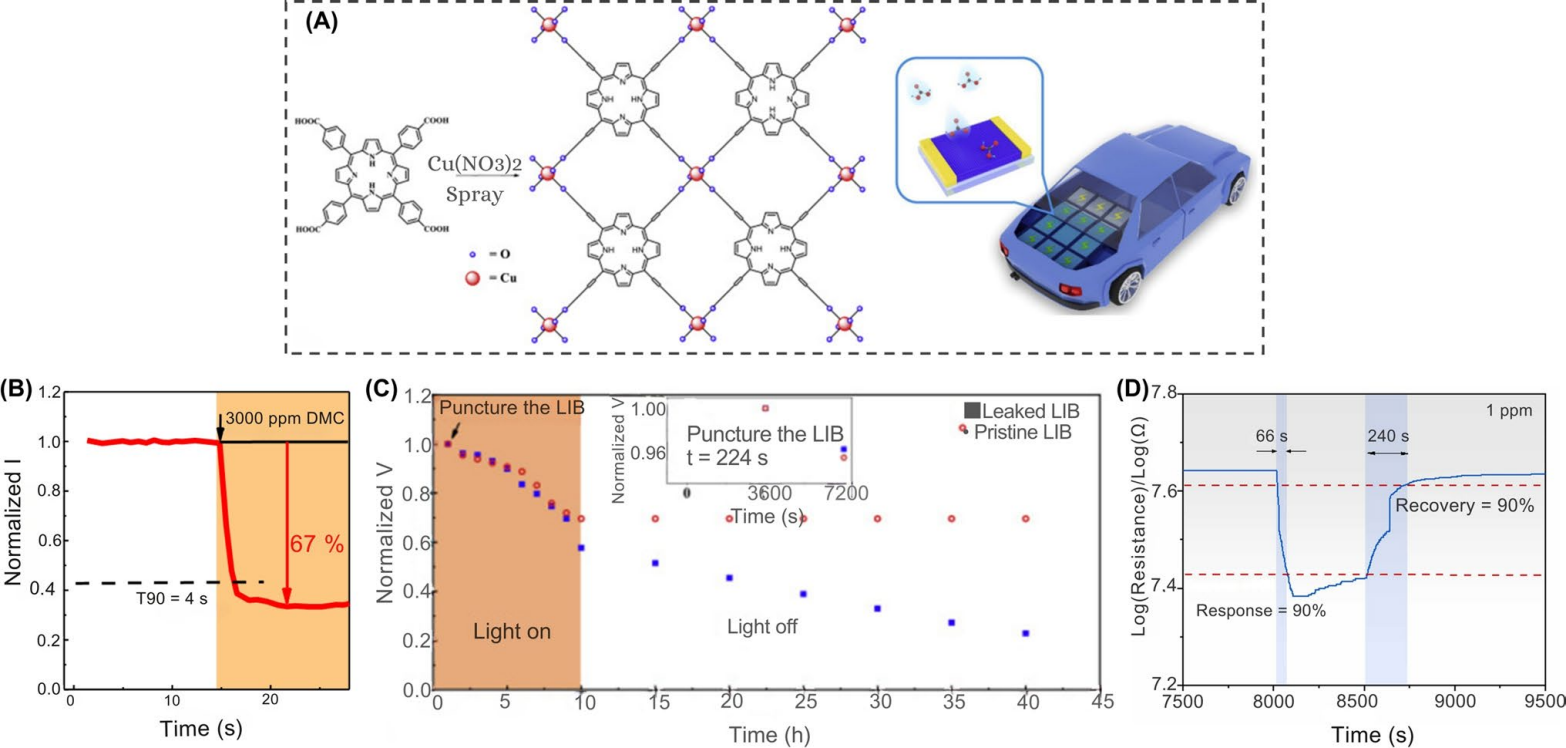
Schematic illustrating the synthetic structure of IC-MOF and the performance of conductometric sensors (both IC-MOF and Co/Pd-doped SnO_2_) for electrolyte leakage detection in LiB. IC-MOF thin films: **A** Synthetic structure of IC-MOF thin films by spraying porphyrin organic ligand solution onto the immiscible aqueous salt solution, and the structure of resultant IC-MOF thin films sensor. **B** Normalized current response of the IC-MOF sensor to 3000 ppm DMC gas. **C** Comparative analysis of normalized voltage between a leaked and a pristine LiB. Co/Pd-doped SnO_2_ sensor: **D** Response and recovery time to 10 ppm DMC at 150 °C. Panels reproduced with permission from **A-C**, ref. [187], Cell Press; **D**, ref. [188], Elsevier Publishing

Conductometric sensors, also known as chemosensors, are instrumental in LiBs monitoring by measuring the electrical conductivity of the battery electrolyte. These sensors provide valuable insights into the electrolyte degradation and states of the battery [187, 188, 193]. In a study by Lu et al., the efficacy of conductometric sensors, particularly those made from ionically conductive metal–organic framework (IC-MOF) thin films, was evaluated for detecting electrolyte leakage in LiBs (Fig. 13A) [187]. The study compared sensing signals based on output current, capacitance, and equivalent resistance. Similarly, their designed sensor operated using AC bias to eliminate the bias stress and improve stable sensing baselines effectively. The sensor was tested with an alternating voltage (1 to − 1 V, then back to 1 V within 2 s) and showed dosimetric responses to DMC (dimethyl carbonate) vapor. This was indicated by changes in the normalized output current (*I/I*_*0*_), with a stable baseline detected under airflow. The exposure to 3,000 ppm DMC/air vapor resulted in a sharp decrease in current, with a response time of about 4 s (Fig. 13B). The sensor also effectively detected 5 ppm DMC vapor, showing an 11% current decrease, and the response was proportional to the DMC concentration, which was attributed to the direct interaction between the analytes and metal ions in the IC-MOF thin films [187]. Furthermore, the study showed rapid and real-time detection of DMC and electrolyte leakage within seconds from LiBs, which provided an early warning time of up to 10 h, indicating a significant advancement in LiBs monitoring (Fig. 13C). The study also demonstrated the high stability of the sensor, with negligible change observed over 6 months in ambient conditions, suggesting its potential for long-term applications [187]. However, while the sensors were evaluated in ambient conditions, they were not tested in extreme temperatures, mechanical stress, or high humidity. Therefore, the potential for performance degradation under heat or physical strain (e.g., in EVs) remains a concern. Similarly, IC-MOFs are prone to corrosion from reactive electrolytes (e.g., LiPF_6_), leading to structural collapse and reduced ionic conductivity over time. While AC bias could improve stability and sensitivity, it complicates power supply design, particularly in large-scale applications (e.g., EVs with hundreds of cells), which requires further optimization for cumulative power consumption and AC circuitry integration

Wan et al. reported a sensor for detecting DMC leakage in LiB electrolytes based on Co/Pd-doped SnO_2_ nanomaterial [188]. Their study synthesized a Co/Pd-doped SnO_2_ sensor with a small, uniform grain size using a sol–gel method, enabling easier control of material properties during the micro-electro-mechanical systems (MEMS) process. The sensor exhibited high sensitivity to DMC, detecting it with a response value of around 1.65–500 ppb at an operating temperature of 150 °C. Moreover, at 1 ppm DMC, the sensor demonstrated response and recovery times of about 66 and 240 s, respectively (Fig. 13D) [188]. The study facilitated early detection of electrolyte leakage and real-time health monitoring of LiBs, preventing potential safety issues and TR events during charge and discharge cycles.

While the Co/Pd-doped SnO₂ sensor exhibited promising performance, these MOS sensors are prone to baseline drift under fluctuating humidity and temperature. Similarly, prolonged exposure to high humidity or reactive gases could degrade performance due to material oxidation or dopant leaching, leading to reduced sensitivity over time. The use of Pd and Co raises concerns about material toxicity and disposal. While Pd is a noble metal with low reactivity, its mining and refining processes are energy-intensive. While the sensor is compatible with MEMS processes for miniaturization, integrating it into sealed battery packs requires robust encapsulation to prevent electrolyte corrosion.

##### Impedance Sensors

Impedance sensors, through EIS, are used to measure the impedance changes of a battery across a range of frequencies. This technology allows for the non-destructive measurements of LiBs, often being integrated into BMS for real-time monitoring. These sensors provide insights into the complex internal reactions and characteristics of batteries, including charge transfer (R_CT_), ion diffusion, and interfacial phenomena, known as SEI [107, 194–196]. Impedance measurements performed at different SoCs and throughout the LiB lifecycle can yield valuable information on internal characteristic changes of the battery during operation, such as short circuits, degradation mechanisms, Li plating, SoH, and electrolyte oxidation. In a study by Zhang et al., EIS measurement was used to obtain impedance data at different time intervals from an LCO battery cell [106]. These data were then integrated into an ML model based on the Gaussian process. The inputs for the model were both the real and imaginary components of the over 20,000 EIS spectra obtained at 60 discrete frequencies, ranging from 0.02 Hz to 20 kHz. The capacity corresponding to each EIS spectra was used as the output for training the model. The model trained could estimate the capacity and predict various stages of LiB degradation and the RUL of the batteries using the EIS spectrum as the key indicator of the SoH in BMS [106]. By integrating EIS measurement and ML models into LiBs, batteries showed the capability to adapt their electrochemical cycling based on real-time data and predictive analytics. This integration has led to enhanced efficiency, performance, and lifespan of LiBs, contributing to the development of smart LiBs.

However, impedance-based models for battery health monitoring require careful data collection and model training to prevent overfitting and enhance interpretability. Since impedance data includes multiple frequency-dependent components, advanced signal processing techniques are needed to extract meaningful information about SoH or degradation mechanisms [107]. Similarly, accurately labeling degradation patterns in impedance spectroscopy data is complicated and requires expert knowledge and manual effort, as inconsistent labeling can introduce errors and reduce the reliability of the ML model. Creating a comprehensive dataset of impedance spectroscopy measurements for various degradation patterns is also laborious and costly. Although impedance measurements have a wide range of capabilities, they are seldom used in BMS. One major limitation is their failure to simultaneously monitor multiple cells in large battery packs, which requires multiplexing circuits, increasing system complexity and cost. Additionally, impedance measurements are bulky, heavy, and power-consuming, making integration into compact BMS challenging [107]. Furthermore, high-resolution EIS generates vast datasets (e.g., 20,000 spectra per measurement), demanding significant computational resources for real-time analysis. It has been reported that module packs, compared to single-cell LiBs, can experience cell mismatch. When one or more cells are mismatched, it poses a significant risk to both the safety and efficiency of the entire LiBs. This can occur due to battery over-discharge, over-charge, internal and external short circuits, or extended periods of inactivity period (calendar aging) [107]. Similarly, conventional BMS using single-frequency impedance (e.g., 1 kHz) lacks sensitivity to detect early-stage mismatches [107].

Carkhuff et al. introduced a small, low-power, multifrequency (1–1,000 Hz) impedance-based BMS, termed battery internal temperature tensor-based BMS (BITS-BMS), for module packs LiBs, enabling the detection of safety-related issues in the anode, cathode, and electrolyte [107]. This system monitored mismatches and abnormalities in electrical and thermal behavior under conditions, such as cycle life aging, calendar life aging, and over-discharge and overcharge. The BITS-BMS could monitor up to 16 cells in module packs (i.e., 80 V and 50 Ah) and optimize thermal safety and efficiency by tracking internal temperature, voltage, and series resistance [107]. In contrast to conventional impedance measurements, which may have limited sensitivity to specific degradation mechanisms, such as electrode surface film formation [106, 197], the BITS-BMS suggested a more accurate and detailed assessment of battery health and performance, overcoming the limitations of conventional impedance measurements. Although BITS-BMS provided unparalleled insights into electrochemical processes, their limitations include high development costs due to sophisticated hardware and computational data, dependency on accurate cell characterization, and challenges in miniaturizing multifrequency circuits for mass production.

## Potential Advancements in LiB Sensor Technology

Advancements in LiB sensor technology are geared toward enhancing the accuracy and reliability of monitoring LiB across various paradigms, including performance, safety, and efficiency. This section highlights key advancements in LiB sensor technology.

### Miniaturization-based Sensors

Sensor miniaturization marks a significant breakthrough in LiB sensor technology, enabling the integration of highly sensitive and accurate sensors within the limited space constraints of LiB devices. Thanks to advanced micro- and nano-fabrication techniques, these sensors can now be scaled down into micro-sized arrays while maintaining their high-performance capabilities [198]. This miniaturization allows for the integration of multiple sensor functionalities into a single battery cell, providing a comprehensive understanding of their performance for smart LiB management [198, 199]. Furthermore, the compact size of these sensors minimizes interference with the battery’s design and functionality. However, miniaturizing sensors for LiBs poses challenges in maintaining sensitivity and accuracy. Research has explored various approaches to tackle these issues [200]. For example, Du et al. developed a portable miniaturized sensor based on functionalized double-walled carbon nanotubes (f-DWCNTs) for the real-time detection of electrolyte leakage in LiBs [200]. The key sensitivity challenge in sensor miniaturization was effectively addressed by covalently functionalizing the outer walls of DWCNTs with hydroxyl groups. This surface modification significantly enhanced the interaction between the nanotubes and DMC, a redox-neutral solvent commonly found in LiB electrolytes, which enabled the sensor to detect trace leakage volumes as low as 0.1 μL. Unlike pristine DWCNTs, which showed weak interactions with DMC, the functionalized version exhibited a markedly improved sensing response with minimal interference from non-electrolyte vapors. The sensor operated reliably at room temperature and demonstrated rapid response and recovery dynamics (t₉₀ values as low as 3.60 s), along with strong long-term stability, retaining its performance even after 85 days of ambient storage. When tested near a leaking commercial LiBs, the sensor recorded an immediate drop in output current, successfully detecting leakage undetectable by conventional voltage monitoring methods. This study not only highlighted the role of targeted chemical functionalization in overcoming miniaturization-related sensitivity loss but also demonstrated a promising method for safety improvement in smart LiBs diagnostics [200]. In a very recent study, ionic gel chemical sensors significantly advanced the miniaturization of LiBs diagnostic technologies by overcoming core limitations that typically compromise sensitivity and long-term reliability in compact sensor formats [201]. Traditional miniaturized sensors often suffer from baseline drift, low sensitivity to redox-inactive electrolyte solvents, and poor environmental resilience. To tackle these challenges, Li et al. developed a capacitive sensor using ionic liquids (ILs) polymerized into highly stable ionic gels, where ions, not electrons, act as charge carriers, enabling superior detection of trace electrolyte leakage [201]. These sensors achieved remarkable sensitivity, detecting DMC volumes as low as 2.3 nL and real LiB electrolyte leakage down to 5.3 nL, with fast response times (~ 3 s) and strong linearity. Most notably, the sensors demonstrated outstanding mechanical resilience, with full self-repairing from scratches within 5 min when heated at 100 °C and stability after 1,500 h under harsh damp-heat conditions (85 °C, 85% relative humidity). Their performance remained consistent even after months of ambient storage. Batch fabrication of a 5 × 5 sensor array further confirmed the design’s scalability and uniformity, essential traits for real-world LiB integration. These advancements exemplify how ionic gel platforms not only preserve but enhance sensitivity and stability at the miniature scale, setting a new benchmark for next-generation smart LiBs monitoring systems [201].

Miniaturization of sensors for LiBs monitoring introduces inherent trade-offs between size, functionality, and performance, particularly affecting signal accuracy, sensitivity, and integration flexibility. To address these challenges, an ASIC-based miniaturized system was developed for online multi-measurand monitoring of LiBs [202]. The researchers integrated the SENSIPLUS chip, a System-on-a-chip that could perform multiple sensing tasks such as EIS, cell voltage monitoring, and temperature measurements, all within a compact 20 × 8 mm^2^ footprint. Integrating such high-resolution analog front-ends and configurable sensor interfaces within the System-on-a-chip ensured the system could maintain a high sensitivity and measurement resolution level, achieving impedance measurements with a precision of 120 µΩ and voltage and temperature readings with negligible offset and noise [202]. Trade-offs in signal loss, noise interference, and cross-talk, typically exacerbated in miniaturized designs, were mitigated through advanced internal signal routing, galvanic isolation, and modular sensor configurations that supported flexible deployment across multi-cell battery packs. Furthermore, trade-offs between miniaturization and durability were addressed by ensuring the robustness of the system, even in demanding environments [202]. The miniaturized design was validated through experiments where the system showed high stability and scalability, which is essential for practical applications in larger battery systems, such as those found in EVs. The ASIC-based miniaturized sensor system demonstrated that sensor miniaturization, when done with integrated approaches and multi-sensor systems, can effectively balance performance, durability, and scalability in practical LiBs monitoring applications [202].

Similarly, miniaturization has been effectively implemented by deploying compact deep learning models on a resource-constrained internet of things (IoT) device for real-time LiBs monitoring [203]. By applying tiny machine learning (TinyML) techniques, the researchers optimized ANN and CNN architectures to estimate the battery’s SoC using minimal hardware resources. To address the typical trade-offs of miniaturization, such as loss of accuracy, limited memory, and processing power, the models underwent post-training quantization (PTQ), particularly using a 16-bit integer format (int16 × 16), which significantly reduced the model’s memory footprint while maintaining high predictive accuracy [203]. The optimized ANN model, occupying less than 3% of the flash memory on the CY8CPROTO-062S3-4343W microcontroller, achieved a mean absolute error (MAE) of just 2.81%, outperforming more complex CNN models in both inference speed and reliability. This approach demonstrated that with appropriate quantization and model simplification, sensor intelligence can be embedded into miniaturized systems without compromising real-world performance, paving the way for efficient, on-device diagnostics in smart BMS [203].

In recent years, researchers have delved into the realm of printing technologies as a means to fabricate miniaturized sensors for LiB applications. This approach has gained significant attention in LiB monitoring due to its inherent low cost, flexibility, and scalability advantages. By utilizing additive-based methods such as screen printing [202], inkjet printing [82], and roll-to-roll printing [204], these sensors could be capably produced on various substrates, including paper and plastic, enhancing their versatility and adaptability [204, 205]. Advanced fabrication techniques, such as the 3D-direct ink writing (3D-DIW) printing method, have opened up new possibilities in constructing heterostructures for various applications. In a recent study, researchers successfully fabricated Al_2_O_3_/CuO (Aluminum oxide/Copper(II) oxide) and CuO: Fe_2_O_3_ (Copper(II) oxide: Iron(III) oxide) heterostructures using the 3D-DIW printing method, followed by atomic layer deposition (ALD) and thermal annealing processes [82]. These heterostructures hold potential for the detection of electrolyte vapors, specifically 1,3-dioxolan (DOL) and 1,2-dimethoxyethane (DME), which are commonly used in LiB [82]. Paljk et al. introduced a miniaturized electrochemical sensor printed directly onto the separator of a LiB to enable in situ detection of dissolved manganese ions, which are known degradation products of Mn-based cathodes such as LMO [204]. The sensor incorporated a manganese ion-imprinted polymer (Mn(II)-IIP) sensing layer positioned between two printed glassy carbon electrodes. This sensor monitored manganese coordination via EIS in the mid-frequency range. Critically, the sensor was electrochemically stable, caused no significant alteration to battery geometry, and exhibited negligible impact on battery performance when integrated into pouch cells. The use of printing technology, compatible with roll-to-roll and sheet-to-sheet processes in their study, offered a scalable manufacturing pathway for commercialization. Their study demonstrated a universal approach for real-time monitoring of cathode degradation, contributing to enhanced battery SoH tracking and safety diagnostics [204].

**Fig. 14:**
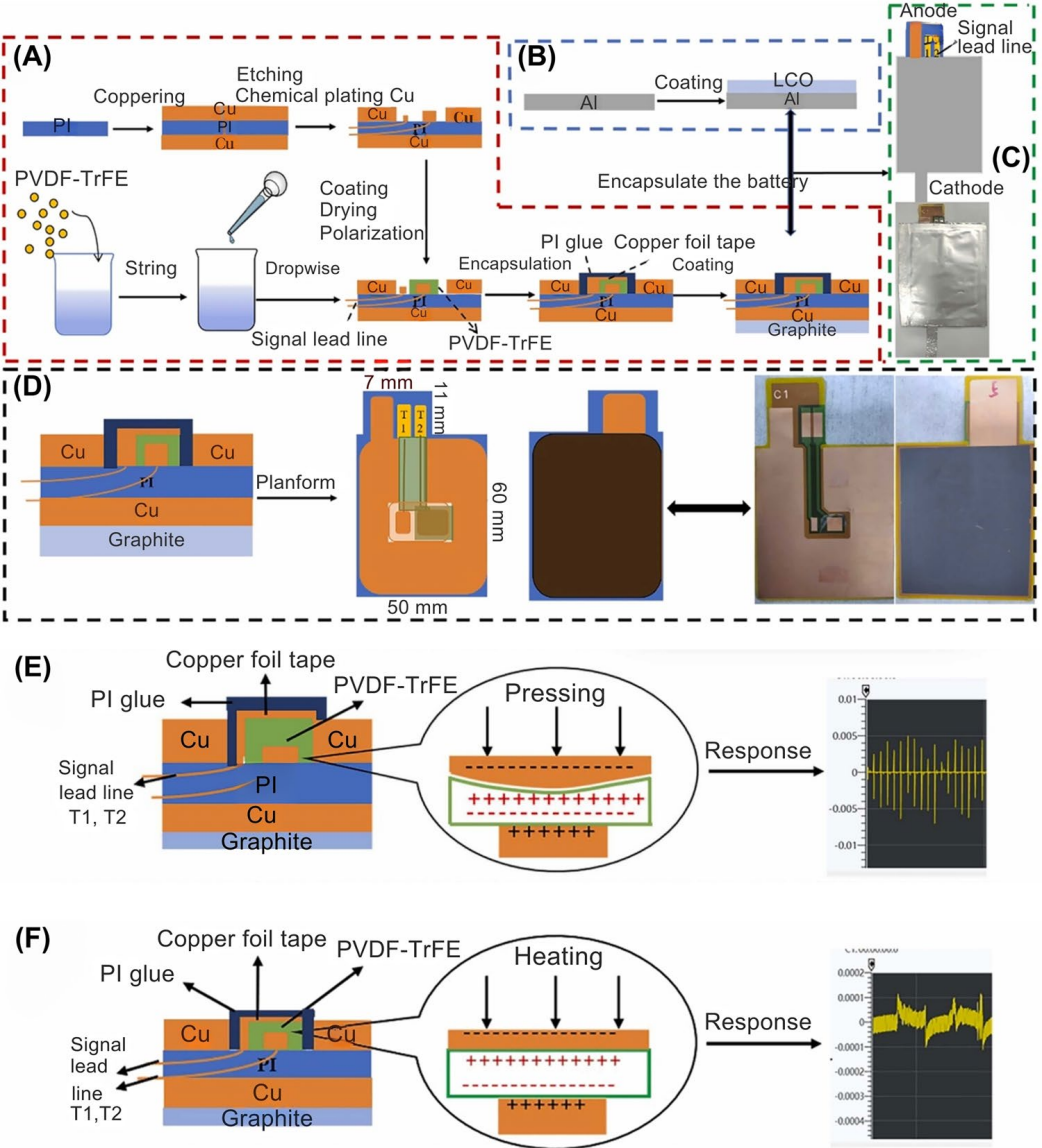
Schematic illustrating the manufacturing sequence of LiBPTMS and its integration into a LiB. **A** LiBPTMS and anode fabrication process. **B** Cathode fabrication process. **C** LiB integrated with the LiBPTMS. **D** Cross-section, top-view, and a sample image of the LiBPTMS based on the PVDF-TrFE film coated on one side of the FPC. This provides a detailed view of the LiBPTMS, Panels **E** and **F** The construction and operational principle of the LiBPTMS, specifically focusing on **E** pressure and **F** thermal damage detection. Panels reproduced with permission from **A-F**, ref. [14], Elsevier

The ability to detect and monitor electrolyte vapors is crucial for ensuring battery safety and optimal performance. Moreover, printed sensors have emerged as a promising technology for simultaneously detecting multiple parameters, such as temperature and pressure [14]. The integration of a LiB pressure/temperature monitoring micro-thin-film sensor (LiBPTMS), constructed from a piezoelectric/pyroelectric PVDF-TrFE (poly(vinylidene fluoride-trifluoroethylene)) material, into a LiB system, could facilitate real-time monitoring of pressure and temperature parameters. Strikingly, this integration had no detrimental impact on the battery operation. The sensor, which is printed, demonstrated the potential for advancements in LiB performance optimization (Fig. 14A-F) [14]. The successful implementation of this sensor suggested the potential to revolutionize BMS, ensuring early warning of LiB failure and significantly improved battery safety performance. Similarly, Manfredini et al. developed a highly miniaturized cell management unit (CMU) based on the SENSIPLUS system-on-chip, a multi-mode sensor interface capable of online multi-measurand monitoring of LiBs [202]. This 20 × 8 mm^2^ PCB-integrated system performed real-time measurements of key battery parameters, including temperature, cell voltage, and internal impedance, via EIS with a resolution of 120 μΩ. The CMU also supported connection to external sensors for detecting moisture and electrode temperatures, making it suitable for comprehensive battery health diagnostics. Designed for scalability, the CMUs communicated over a simplified I^2^C or proprietary SENSIBUS protocol with galvanic isolation, enabling multi-cell series monitoring without complex wiring. The compact and modular design was compatible with commercial Li-polymer cells and showcased minimal impact on battery operation, marking a significant step toward distributed BMS architectures and industrial deployment of embedded miniaturized sensor systems for monitoring multi-cell LiB packs [202].

**Fig. 15:**
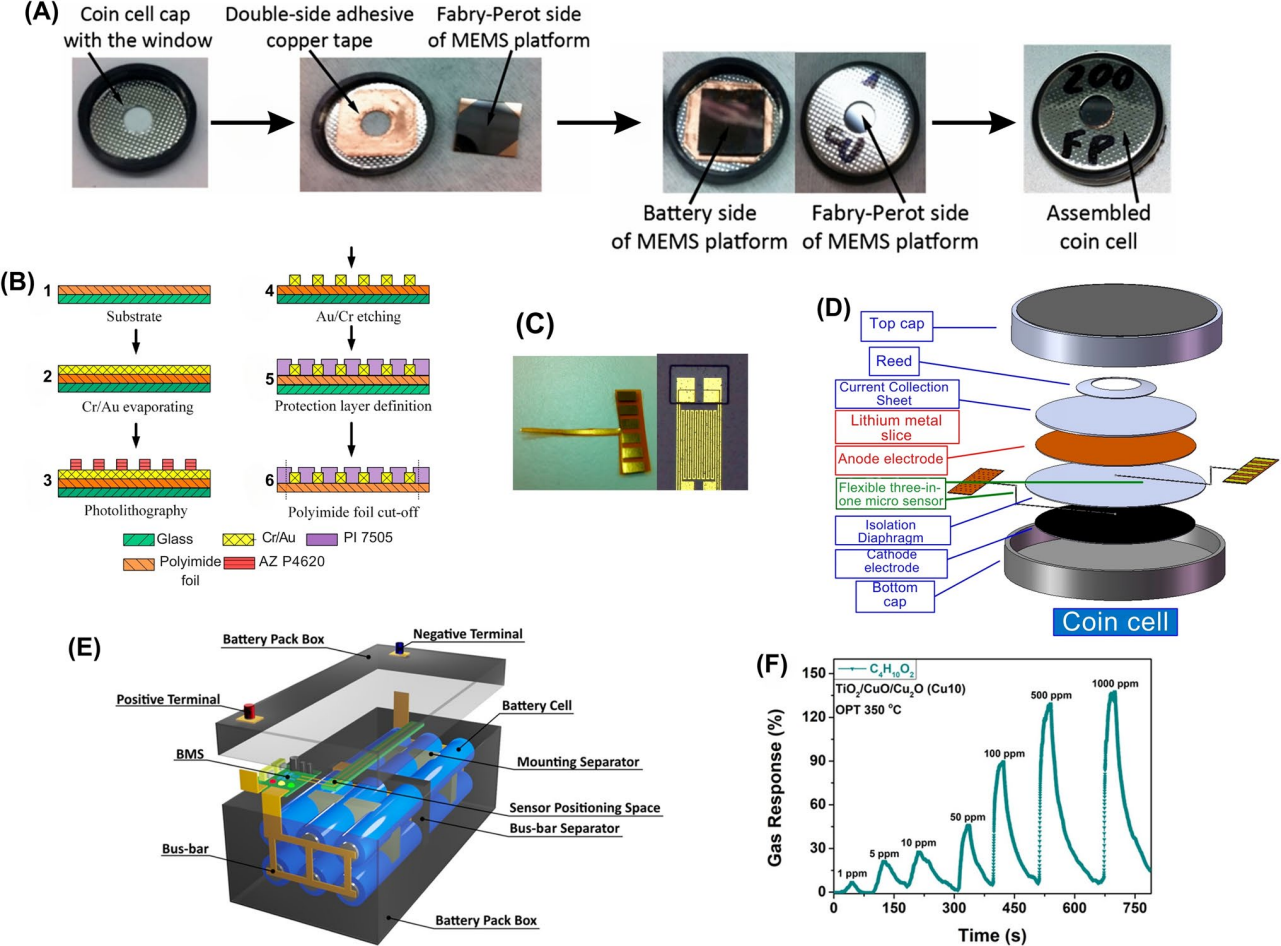
MEMS sensor technology fabrication for LiB monitoring. **A** The packaging process is initiated by machining a circular window in one half of a coin cell. Subsequently, double-sided adhesive conductive tape is utilized to mount the device, thereby making the Pyrex surface of the device visible through the window. The addition of electrolytes, separators, and lithium is carried out inside a glove box to prevent contamination. The package is then sealed to secure the components. **B** Production process of a flexible three-in-one microsensor. This involves the integration of three different sensing elements into a single, flexible device. The final product is shown in **C** accompanied by an optical micrograph that provided a detailed view of the sensor structure. **D** Schematic diagram of the flexible three-in-one microsensors package assembly embedded in a LiB coin cell. This diagram provides a visual representation of how the sensors are integrated into the coin cell, highlighting the compact and efficient design of the device. **E** Schematic concept of the battery pack. **F** Dynamic response of TiO_2_/CuO/Cu_2_O samples with thicknesses of 10 nm (denoted as Cu10) at an operating temperature of 350 °C to 1, 5, 10, 50, 100, 500, and 1000 ppm of C_4_H_10_O_2_ vapors. Panels reproduced with permission from **A**, ref. [208], IOP; **B-D**, ref. [198], MDPI; **E, F**, ref. [211], ACS Publishing

Another approach to miniaturization is the use of MEMS technology. MEMS sensors are fabricated using processes similar to those employed in the semiconductor industry, which can be scaled down to dimensions as small as a few microns [206, 207]. MEMS-based sensor technology has gained significant interest in LiB sensing because of its potential for miniaturization, high sensitivity, low power consumption, and low cost. This technology has found diverse applications in LiB systems, including temperature sensing, strain sensing, and gas sensing [41]. A study demonstrated the effective utilization of an optical MEMS sensing method in characterizing the reversible mechanical changes in LiB electrodes induced by electrochemical processes (Fig. 15A) [208]. Similarly, the development of miniaturized flexible micro-temperature sensors using MEMS technology and their integration into LiB allowed for real-time temperature monitoring without adversely impacting the battery structure (Fig. 15B-D) [41, 198]. In another study, Lee et al. developed a miniaturized integrated microsensor using MEMS technology for real-time, in situ monitoring of LiBs [209]. This integrated microsensor simultaneously measured internal temperature, voltage, and current by embedding microscale RTD temperature sensors, voltage probes, and current sensors directly within the battery structure. Designed on a flexible polyimide substrate, the sensors featured rapid response (< 1 ms), high accuracy (temperature error < 0.5 °C), and minimal impact on battery performance (only ~ 1.68% capacity deviation). These sensors were embedded in full coin cells and subjected to various C-rate charge/discharge cycles, enabling microscopic-level observation of thermal and electrical behaviors. Their work demonstrated compatibility with batch manufacturing, a practical and scalable approach toward miniaturized sensor integration for enhanced safety and performance diagnostics in commercial LiB systems [209]. Furthermore, Tan et al. developed a miniaturized MEMS-assisted fiber-optic fabry–perot pressure sensor for operando gas pressure monitoring inside commercial 18,650 LiBs [210]. This sensor, combining MEMS and optical technologies, was embedded directly into LFP and NCM523 cells to track internal gas pressure in real-time without altering battery electrochemistry. The sensor featured high-pressure sensitivity (72.557 nm kPa^−1^) and ultra-low temperature cross-sensitivity (0.0413 kPa °C^−1^), enabling precise detection of pressure fluctuations due to gas generation and electrode lattice volume changes during charge/discharge cycles. Their method demonstrated stable, reproducible performance and minimal impact on battery capacity. By eliminating temperature effects, the system linked pressure variations directly to electrochemical and structural changes in the electrode materials. Their study provided a scalable, high-precision solution for in situ battery diagnostics with direct relevance to industrial battery gas pressure monitoring and performance evaluation [210]. This technological advancement has significant implications for the future of LiB sensing, as it enables miniaturization and integration with other electronic components, potentially improving the performance and safety of LiB in various applications.

### Nano-Based Sensors

Modifying materials at the atomic and molecular scales could significantly enhance the performance of sensors for LiBs monitoring. The evolution of sensors by the emergence of nanostructured materials, including carbon-based nanomaterials (e.g., graphene, CNTs), metals oxide-based (e.g., MnO, Nb_2_O_5_) nanomaterials, and polymer-based nanomaterials (e.g., silicones) has significantly driven the development of sensors with superior sensitivity, selectivity, and stability [200, 211]. These materials exhibited unique properties, making them highly versatile for sensing applications in LiBs. The inherent characteristics of these nanomaterials are conducive to the creation of a diverse spectrum of sensors encompassing optical, electrochemical, and physical properties. For example, a study investigated a sensitive chemical sensor utilizing IC-MOFs developed for a portable sensing system by connecting the IC-MOFs sensor to a Bluetooth transmission printed circuit board for real-time monitoring of LiB electrolyte leakage [187]. The sensor detected trace amounts of DMC, allowing for the measuring of electrolyte leakage as small as 20 nL at room temperature [187]. Furthermore, advanced nanosensors, using the principles of nanotechnology, have been reported to detect gas vapors at ppm levels [212–214]. Lupan et al. investigated the gas-sensing capabilities of semiconducting metal oxides for detecting vapors emitted by various components commonly found in LiBs, such as solvents (1,2-dimethoxyethane), salts, or their degassing products (Fig. 15E) [211]. Their study revealed that the semiconducting metal oxide sensors were highly effective in detecting the vapors produced by battery solvents and degassing products up to a concentration of 1000 ppm and 136%, respectively (Fig. 15F) [211]. These sensors exhibited high accuracy, which could serve as high-performance battery safety sensors to prevent potentially explosive vapors from malfunctioning LiBs.

Similarly, integrating graphdiyne-coated carbon nanofibers with a polymeric-TETAT (Triethyl 1,3,5-triazine-2,4,6-tricarboxylate, C_12_H_18_N_3_O_6_) doped with copper resulted in the development of a selective, memory-based sensing film [215]. This sensor could detect carbonates and hydrofluoride compounds at concentrations as low as 10^−2^ ppb, providing good signal resolution and real-time monitoring capabilities, particularly effective for identifying issues related to electrolysis and electrolyte leakage in EV battery systems [215]. In another study, Zhu et al. designed and synthesized a highly sensitive gas sensor based on amorphous bimetallic oxide CuSnO_3_ (CSO) loaded onto cubic In_2_O_3_ (CSO/In_2_O_3_) for detecting DME in LiBs [212]. By synergistic catalytic effects of Cu and Sn atoms in the amorphous CSO structure and forming a heterojunction with In_2_O_3_, the sensor performed a superior response of 6.2 to 20 ppm DME, with a low detection limit of 0.1 ppm. The practical application of the sensor in their study showed early warning of TR in LiBs used in EVs. By detecting trace amounts of DME, the sensor served as a compact, cost-effective, and easily integrable solution for real-time safety monitoring in BMS. The sensor demonstrated practical applications due to the sensor’s stability and repeatability [212]. Zhang et al. developed a highly sensitive sensor based on organic field-effect transistors (OFETs) for the early detection of LiBs electrolyte leakage [216]. The key innovation of their study was the use of a biurea receptor layer on the OFETs, which significantly enhanced the sensitivity and selectivity of the sensor. The sensors demonstrated an impressive detection limit of 1.4 ppm for DEC, a common electrolyte solvent, and could detect trace amounts of electrolyte leakage (as little as 200 nL) within seconds. The sensor’s response was robust, with a 3% current change observed even at low leakage volumes, providing good signal resolution and real-time monitoring capabilities for identifying issues related to electrolysis and electrolyte leakage in LiBs [216]. Lupan et al. prepared a highly sensitive dual-mode nano-based sensor using europium-doped zinc oxide (ZnO:Eu) nanowires for H_2_ gas detection in LiB systems used in EVs [213]. These nanosensors, fabricated through electrochemical deposition and integrated as single nanowires, demonstrated a remarkably high gas response up to 7860 at 150 °C for 100 ppm H_2_ due to surface functionalization with Eu_2_O_3_ nanoparticles. The device also functioned effectively under room temperature and UV irradiation, allowing selective H_2_ detection in UV-rich environments, such as those involving TR or combustion. DFT simulations further confirmed enhanced H_2_ adsorption on Eu-modified ZnO surfaces, correlating with improved sensor performance. Notably, the sensors maintained performance under varying humidity and after long-term storage, highlighting their robustness, scalability, and integration in EVs for real-time H_2_ leak detection and safety diagnostics [213]. Zhang et al. manufactured a room-temperature MEMS H_2_ sensor using Pt-modified Nb-doped TiO_2_ nanosheets as the sensing material [214]. The sensor obtained high sensitivity to H_2_ gas even under hypoxic (low-oxygen) conditions, mimicking the environment inside LiB packs. The sensor demonstrated a strong response (12.3) to 1000 ppm H_2_ at room temperature, with rapid response and recovery times (31 and 270 s, respectively), low power consumption (0.1 mW), and a compact footprint (0.05 cm^3^). These enhancements were attributed to increased oxygen vacancies from Nb doping and the catalytic activity of Pt, which facilitated H_2_ molecule dissociation. The practical application of the study emphasized the early detection of H_2_ gas emissions in LiB systems**,** particularly for TR monitoring in EVs. The sensor could be operated effectively in oxygen-free environments, making it ideal for integration into LiB packs for real-time safety diagnostics [214]. This capability to detect minute changes at the nanoscale can potentially lead to improved LiB performance and safety, ultimately contributing to the development of more efficient EVs.

Integrating nano-based sensors into LiBs offers transformative capabilities for real-time monitoring of ion diffusion, mechanical strain, and thermal anomalies, enhancing battery performance and safety. However, implementing nanomaterials introduces potential safety and regulatory challenges that require rigorous risk assessments to ensure sustainable deployment in practical applications. Nanomaterials possess a high surface area-to-volume ratio, which can lead to heightened chemical reactivity. This increased reactivity may result in unintended side reactions within the battery environment, potentially compromising the stability and longevity of both the sensor and the battery. For example, unwanted interactions between nanomaterials and battery electrolytes may accelerate degradation processes, increasing the risk of TR. In addition, nanostructured materials may undergo structural changes over time, such as agglomeration or fragmentation, which can degrade sensor performance. This degradation affects the accuracy of battery monitoring and may also introduce contaminants into the battery system, further impacting safety and efficiency. Certain sensors that use metallic components (e.g., Cu, Sn, In, Au) may accelerate the decomposition of electrolytes or spark ignition in extreme thermal reaction scenarios if not properly isolated. Therefore, robust encapsulation and thermal insulation are essential in their design. For example, CuSnO_3_/In_2_O_3_ composites may accelerate the decomposition of LiPF_6_, a common electrolyte salt in LiBs. Moreover, many nanomaterials-based sensors (e.g., metal oxides, CNTs, graphdiyne, and MOFs) pose toxicological risks during volume manufacturing, handling, or disposal. For example, nanoparticles can penetrate biological membranes and cause cytotoxic effects. Ensuring occupational safety and compliance with regulations such as the Registration, Evaluation, Authorisation, and Restriction of Chemicals (REACH) or environmental protection agency (EPA) guidelines is crucial during sensor fabrication and end-of-life disposal. While sensors such as IC-MOFs and nanowire-based MEMS showed excellent lab-scale results, scalability, reproducibility, and yield remain concerns for mass production. Electrochemical deposition and photopolymerization can be promising, but large-scale deployment requires rigorous process validation and cost analysis [187, 214].

Furthermore, sensors must operate safely under harsh LiB electrochemical conditions. While high sensitivity may increase the risk of false positives or drift, particularly in complex battery environments with fluctuating humidity and VOCs. Thus, long-term baseline stability is critical for reliable performance. Devices such as ionic gel sensors [201] or IC-MOF-based sensors [187] addressed these challenges by exhibiting damage tolerance and long-term operational stability. Recently, Li et al. developed a new class of ionic gel-based chemical sensors designed for real-time monitoring of LiB electrolyte leakage, effectively mitigating issues of baseline drift and sensor degradation in complex and fluctuating environments [201]. Unlike conventional sensors that rely on electron transport and often suffer from charge-trapping effects and false positives, these sensors used ions as charge carriers, which provided improved stability and sensitivity. The ionic gels were synthesized via one-step photopolymerization of ionic liquids and incorporated into a thin film with exceptional consistency, thermal stability, and healing capability after physical damage. Notably, the devices could maintain performance even after 1,500 h under high humidity and temperature, and their long-term operational stability was demonstrated without significant signal drift. They exhibited rapid, reversible responses to trace amounts of electrolyte components, including DMC and DME, and successfully detected different stages of LIB TR, positioning them as robust, scalable, and damage-tolerant solutions for reliable LiB safety diagnostics​ [201]. When integrating such nano-based sensors into BMS, electromagnetic interference (EMI), signal integrity, and low-power design must be considered. Some MEMS-based or OFET sensors operate at ultra-low power (e.g., 0.1 mW), but others may require more power or complex calibration routines [214]. Regulatory compliance with automotive or aerospace electronic standards, such as ISO 26262 for functional safety, is necessary. Currently, there is a lack of standardized testing protocols and certification pathways for nanosensor-enabled battery safety systems. Collaboration with certification bodies (e.g., UL, IEC, ISO) is necessary to validate sensor performance under standardized abuse tests (e.g., overcharge, crush, puncture, and fire scenarios). Regulatory frameworks must evolve to support the safe integration of nanotechnology in sensors for battery systems.

### Machine Learning Model-Based Sensors

Machine learning (ML) models enhance BMS by analyzing and integrating sensor data from LiBs to create predictive models that improve accuracy and efficiency [217]. This integration facilitates predicting and preventing potential failures, optimizing battery performance, and extending battery lifespan, which is critical for applications ranging from EVs to renewable energy storage systems. A crucial phase in developing these predictive models involves data preprocessing and feature selection, where raw sensor data is prepared for analysis [218–220]. Techniques such as principal component analysis (PCA) and linear discriminant analysis (LDA) are commonly employed to mitigate noise, handle outliers, and extract relevant features from datasets, enhancing the performance of ML algorithms in BMS applications [218, 220]. In a study, Ma et al. introduced a multi-fault diagnostic framework for series-connected LiB packs that used PCA and kernel PCA to analyze key parameters (e.g., ohmic resistance, terminal voltage, and open-circuit voltage) [221]. Their model could detect deviations indicative of faults by comparing individual cell performance against a median cell benchmark. Their method estimated fault waveforms and established fault indexes by considering joint parameter variations, which enhanced fault diagnosis reliability. This approach not only improved real-time abnormality detection but also offered a comprehensive assessment of battery pack health [221].

ML algorithms excel at uncovering intricate patterns and nonlinear relationships within sensor data, suppressing the limitations of traditional analytical methods [222]. These capabilities are particularly valuable for predictive analytics, where historical datasets are leveraged to build models that accurately forecast system behavior and component failures [222]. For example, deep neural networks (DNN) have demonstrated exceptional performance in predicting the RUL of LiBs due to their ability to autonomously learn degradation patterns and generalize across diverse operating conditions [222]. Cai et al. developed a hybrid model for early RUL prediction of LiBs by integrating data decomposition, transformers, and DNNs [223]. Their approach utilized complete ensemble empirical mode decomposition with adaptive noise (CEEMDAN) to handle capacity regeneration effects and extract meaningful degradation patterns. Transformer networks were employed to predict local fluctuations in capacity, while a DNN was used to model the global degradation trend. The model was validated using two publicly available battery datasets, achieving high prediction accuracy with only 25%-30% of lifetime data. Their findings showed that their hybrid model outperformed existing methods in RUL estimation, demonstrating its potential for real-time practical battery health monitoring​ [223]. Similarly, a study by Han et al. introduced a denoising transformer-based neural network (DTNN) model specifically designed for RUL prediction of LiBs. The DTNN demonstrated superior accuracy and reliability compared to traditional ML models and other deep learning architectures, achieving a mean absolute percentage error (MAPE) of 0.632% and an absolute RUL error of 3.2 cycles. This performance underscored its potential to provide significant benefits for BMS through accurate RUL predictions, promising practical applications [224].

A critical application of ML lies in the estimation of SoC and SoH, which are vital for preventing overcharging, over-discharging, and thermal risks. For example, ANN trained on voltage, current, and temperature data have achieved less than 2% SoC estimation error in dynamic EV driving cycles, outperforming traditional coulomb counting methods [225–227]. In a study, Vieira et al. developed an ANN model to estimate the SoC of LiBs using a dataset from NASA’s research center, proposing a novel training approach based on the maximum correntropy criterion (MCC) instead of the traditional mean squared error (MSE) function [228]. Their approach aimed to improve SoC estimation accuracy by incorporating higher-order statistical moments, making the model more robust to non-Gaussian error distributions and outliers. To optimize MCC performance, they employed adaptive strategies and genetic algorithms to fine-tune the width of the Gaussian kernel used in the error evaluation process. Their results demonstrated that the MCC-based ANN model outperformed MSE-based models in SoC estimation, particularly in handling noisy datasets and reducing estimation errors, making it a promising approach for real-time battery monitoring in electric vehicles [228]. Similarly, Wang et al. proposed a physics-informed neural network (PINN) for accurate and stable SoH estimation of LiBs, addressing the challenges posed by diverse battery chemistries and operating conditions [229]. Their approach integrates empirical degradation models and state-space equations with neural networks to capture battery degradation dynamics effectively. A novel feature extraction method was introduced, focusing on statistical features from a short charging period to enhance generalization across different battery types and charge–discharge protocols. To validate their model, they compiled a dataset of 55 NCM batteries and combined it with three additional datasets, totaling 387 batteries with 310,705 samples. The proposed PINN achieved a MAPE of 0.87%, demonstrating superior performance in a regular, small sample, and transfer learning experiments compared to alternative neural network models [229]. Giazitzis et al. developed a tiny ML (TinyML) application for real-time battery SoC estimation deployed on a low-power IoT device, specifically the Infineon CY8CPROTO-062S3-4343W [203]. They trained and optimized compact ANN and CNN models using a large dataset of over 7.5 million samples from LG 2.5 Ah 18,650 NMC LiBs. The models were quantized using Infineon’s ModusToolbox ML software to enable on-device inference with minimal computational and memory requirements. The ANN model achieved high accuracy (MAE as low as 2.81%) with significantly lower memory usage than CNN, making it ideal for resource-constrained edge devices. Their work showed a practical and scalable implementation of miniaturized smart sensors using TinyML for embedded SoC estimation in LiB management systems [203].

Moreover, support vector machines (SVM) have been deployed to detect micro-shorts in LiB cells by analyzing deviations in voltage hysteresis, enabling early fault detection with 95% accuracy. These models utilize high-resolution raw sensor data (e.g., voltage curves, impedance spectra) to infer battery states, often incorporating feature engineering techniques such as time-domain filtering or frequency-domain transforms to enhance signal clarity [230, 231]. For example, Yao et al. proposed an intelligent fault diagnosis method for LiBs based on grid search-optimized SVM [230]. Their approach involved using discrete filtering to denoise voltage data, followed by the introduction of an MCM to minimize the influence of current fluctuations on fault indicators. The model was trained using optimized SVM, where the kernel function and penalty factor were fine-tuned via a grid search to enhance classification accuracy. The results demonstrated that the proposed method achieved over 95% detection accuracy while significantly reducing computational time, making it a viable solution for real-time battery system fault detection in electric vehicles [230]. By integrating real-time sensor inputs with adaptive learning frameworks, ML-driven systems improve safety and extend battery lifespan through optimized charging protocols. Xiao et al. developed model-based virtual thermal sensors (VTS) for automotive-grade LiB monitoring [39]. The model input parameters, including the density of the plastic case, the thermal conductivity of the case, the heat capacity of the case, the heat capacity of the battery core, and the experimentally measured heat transfer coefficient (HTC), were obtained from the battery dimensions, material properties, and data-measured HTC. In their study, to enhance the accuracy of the input parameters, the estimated thermal states were fitted to real measurements using both the prediction error minimization method and the system identification toolbox in MATLAB software [39]. Their findings demonstrated that the VTS could accurately estimate internal temperatures, suggesting a cost-effective and reliable alternative to direct internal temperature measurements of LiBs in EVs applications [39].

Furthermore, the integration of ML models into prognostics and health management (PHM) systems has significantly facilitated the development of LiBs monitoring [232, 233]. These systems also leverage real-time sensor data, such as voltage, current, temperature, and impedance, to accurately estimate SoH and predict RUL, enabling proactive maintenance and fault detection [232]. For example, random forest (RF) algorithms have been deployed in commercial EVs BMS to analyze voltage hysteresis patterns, identifying early-stage capacity fade caused by Li plating [234–237]. Li et al. developed a numerical simulation-based ML model to predict LiBs capacity fade, integrating electrochemical modeling with digital-twin datasets [234]. They employed neural networks for regression-based capacity prediction and used RF algorithms for feature importance analysis. The RF model identified upper cut-off voltage (UCOV) as the most critical factor influencing battery degradation, followed by temperature and charge/discharge rates. Their study, combining ML with high-fidelity numerical simulations, achieved a prediction error of less than 2%, significantly accelerating battery degradation analysis while reducing experimental costs by 99% [234]. Similarly, Zhang et al. developed an interpretable battery lifetime prediction framework using early degradation data, employing quantile regression forests (QRF) to provide both point and range predictions with quantified uncertainty [238]. The QRF model demonstrated superior performance in predicting cycle life compared to traditional ML models, as it does not assume a specific distribution of cycle life data. In addition, the study employed two model-agnostic interpretation techniques to rank feature importance and analyze their quantitative effects on LiBs degradation predictions. Their approach also included a capacity knee identification algorithm based on unsupervised time-series segmentation, effectively identifying capacity knee-onset points in experimental datasets. Their study revealed the advantages of QRF for decision-making under uncertainty, particularly for selecting high-cycle-life fast-charging protocols [238].

Similarly, studies have demonstrated the effectiveness of long short-term memory (LSTM) networks in predicting the RUL of LiBs under dynamic load conditions, achieving a mean absolute percentage error (MAPE) of less than 5% [233, 239]. Park et al. developed a novel LSTM-based approach for predicting the RUL of LiBs, leveraging multi-channel charging profiles that include voltage, current, and temperature data [233]. Their model utilizes a many-to-one LSTM structure, which significantly reduces the number of parameters while improving generalization and prediction accuracy. Using the NASA LiBs dataset, the proposed multi-channel LSTM (MC-LSTM) model achieved a MAPE between 0.47% and 1.88%, suppressing conventional LSTM models by up to 63.7%. Their study demonstrated that integrating multiple sensor inputs enhances RUL estimation, capturing complex battery degradation patterns, including capacity regeneration effects, which are often overlooked in traditional single-channel models [233].

The ability to predict LiBs degradation using early-cycle data marks a significant advancement in battery analytics. This approach utilizes ML to predict long-term performance and cycle life from limited initial cycling data [235, 240, 241]. Saxena et al. developed a convolutional neural network (CNN) model to predict the entire battery capacity fade curve using only the initial 100 charging cycles of LiBs [240]. Their study automated feature extraction from discharge voltage-capacity curves and incorporated a bilinear equation to describe capacity fade trends, including the fade rate and knee point. The model was trained and validated using a dataset of 178 graphite/LiFePO_4_ batteries, achieving MAPE of 3.7% for capacity fade predictions, 19% for rollover cycle identification, and 17% for end-of-life estimation. By leveraging CNN-based modeling, their study demonstrated the potential for early prediction of LiBs degradation, enabling improved battery selection, quality control, and lifespan optimization [240]. Similarly, it has been reported that computational and ML models can reveal hidden relationships between battery performance and operating conditions, allowing predictions of LiB cycle life based solely on early cycle data before any capacity degradation occurs [235]. Tian et al. developed a data-driven computational and ML model to predict LiB degradation by forecasting voltage-capacity curves using a sequence-to-sequence (Seq2Seq) neural network [237]. Their study enabled long-term prediction of voltage-capacity characteristics based on data from a single cycle, avoiding the need for extensive historical datasets. The model demonstrated accurate multi-cycle-ahead forecasting, effectively capturing degradation trends across different battery chemistries while reducing reliance on traditional feature engineering. In addition, they integrated an RF model for battery health estimation, further validating the robustness of their method. This system enhanced predictive BMS by allowing early intervention and optimized maintenance planning [237]. In another study, Buchanan et al. developed a hybrid ML framework integrating CNNs and Gaussian process regression (GPR) for probabilistic SoH estimation of LiBs [242]. Their model utilized CNNs for feature extraction from partial charge cycles and applied GPR to provide probabilistic predictions with confidence intervals. The CNN-GPR approach demonstrated robust performance across dynamic charge–discharge protocols, achieving an MAE of less than 1% while adapting to inconsistent inputs. This probabilistic estimation method is particularly beneficial for applications in EV fleets and microgrid energy storage, where partial charge cycles are common. Their study suggested the potential of transformer-based architectures to further enhance battery health predictions by capturing long-term dependencies in SoH estimation [242].

Another significant advancement in ML model-based sensors has facilitated data integration from multiple sensors, which has developed sensor fusion technology to enhance fault detection and battery health estimation, suppressing traditional single-sensor approaches for smart LiBs monitoring [243]. Techniques, such as decision level or feature fusions, have been effectively employed to enhance the detection capability and accuracy in sensors designed for more robust and accurate monitoring of LiB cells [244, 245]. In a recent study, Yifan et al. developed a fault diagnosis and early warning method for LiBs based on a multi-feature fusion model [244]. Their approach integrated data from multiple sensor modalities, including voltage, temperature, and internal resistance, using a combination of threshold-based, statistical, and model-based feature extraction techniques. Their study employed an RF algorithm to fuse these features and enhance fault detection accuracy, particularly in identifying early-stage internal short circuits and capacity degradation. By analyzing a large real-world dataset from 400 EVs, the proposed method demonstrated improved generalization and reduced false alarm rates compared to traditional single-sensor approaches. The study highlighted the potential of multi-feature fusion techniques in enabling more accurate and interpretable ML-based LiB monitoring for EV applications [244]. Xie et al. developed a multi-parameter fusion early warning method for TR of LiBs, integrating voltage, temperature, and gas sensor data using a cloud model and Dempster–Shafer (DS) evidence theory [246]. Their approach accounted for the fuzziness and uncertainty inherent in individual sensor measurements, enhancing the reliability of TR risk assessment. By fusing multiple sensor outputs, the method could improve the accuracy of early warnings, addressing the limitations of single-parameter threshold models, which often result in high false alarm rates. Their study demonstrated that the proposed fusion-based warning system effectively categorizes risk levels and enables proactive intervention, making it a promising tool for real-time LiB safety monitoring [246]. Similarly, Zhang et al. developed a multi-modality, multi-classifier fusion decision algorithm for LiB leakage fault diagnosis by integrating decision-level fusion techniques to enhance detection accuracy based on real-vehicle data [245]. Their approach combined EIS, voltage signal analysis, and incremental capacity bar graphs with cloud-based threshold alarms, forming a comprehensive feature matrix for ML-based fault diagnosis. By leveraging multiple classifiers, including RF, extreme gradient boosting (XGBoost), and SVM, the model achieved an early warning capability of up to 26 days in advance on real-vehicle datasets. The fusion of multiple sensor outputs improved fault quantification and hazard scoring, significantly suppressing traditional threshold-based systems in detecting electrolyte leakage and preventing TR [245].

However, implementing ML models in real-world BMS faces several interconnected challenges. Firstly, the reliability of sensor data is compromised over time due to the harsh electrochemical environments within LiBs, leading to noisy data that can degrade ML model predictions [231]. To mitigate this, employing redundant sensor arrays and self-calibration algorithms, such as Kalman filters, can enhance data integrity. Another critical issue is the computational complexity of advanced ML models, such as CNN or LSTM networks, which often exceed the processing capabilities of embedded BMS hardware, leading to latency in real-time decision-making [247]. Furthermore, ML models trained on specific battery chemistries, such as NMC, may fail to generalize to others, such as LFP, due to differing degradation patterns, requiring transfer learning techniques to adapt models across chemistries with limited datasets. Similarly, as batteries age, shifts in data distributions caused by capacity fade or internal resistance changes lead to model drift, diminishing prediction accuracy unless online learning frameworks continuously update models with fresh data. Integration challenges also arise from proprietary BMS firmware lacking standardized application programming interfaces (APIs), complicating the deployment of ML solutions [248, 249]. However, middleware platforms, such as cloud-based BMS and the IoT Greengrass, can address the challenges of conventional BMS, suggesting seamless communication between ML models and existing systems to enhance battery efficiency, safety, and reliability [248, 249]. Furthermore, the durability of electrochemical sensors in harsh battery environments affects data quality, making it difficult for ML models to distinguish between true battery anomalies and sensor malfunctions. The performance of these models is inherently linked to the volume and quality of training data, where insufficient or unrepresentative datasets can limit their ability to capture diverse operating conditions [231]. Similarly, effective feature selection poses a challenge, as ML models need to prioritize relevant parameters such as voltage hysteresis, temperature gradients, and impedance spectra to accurately predict states such as SoH or RUL [250]. Finally, the intrinsic limitations of ML algorithms, such as ANN susceptibility to overfitting, SVM sensitivity to feature scaling, and decision tree complexity, require careful tuning and regularization to balance accuracy and computational efficiency [251–253]. Addressing these challenges is essential for deploying robust, adaptive ML-driven BMS that can enhance battery safety, longevity, and performance in real-world applications.

### Wireless Sensor Networks-Based Sensors

The development of wireless sensor networks (WSNs) for LiB monitoring offers unique opportunities for comprehensive, distributed surveillance of battery performance and safety parameters. The WSNs comprise multiple wireless sensor nodes, usually with reduced size, that collect data from different sensors installed in the battery system [254, 255]. Each node possesses a processor, memory, sensors, a wireless communication module, and a power source [255]. The wireless nature of the sensor nodes eliminates limitations associated with wired systems, such as complexity, weight, and physical constraints, while also reducing installation and maintenance costs. Similarly, it allows for flexible placement of the sensor nodes, making it easier to monitor large battery packs or LiB-based systems in remote locations [256, 257]. As EVs become more autonomous, the integration of machine-to-machine (M2M) communication is also becoming essential. M2M communication enables devices, such as sensors, to exchange information autonomously without human intervention, facilitating direct communication between the vehicle’s BMS and charging or battery swapping stations [258]. This capability ensures that the necessary actions, such as initiating charging or swapping, can occur automatically based on the real-time battery data received from the sensors. M2M systems enable a seamless connection across different EV components and external infrastructure, such as charging networks, thus enhancing the operational efficiency and safety of the entire EV ecosystem [258]. In a study by Florea and Taralunga, a WSN for EV battery management was proposed, with a focus on improving the charging and battery swapping systems [258]. Their study introduced the idea of using blockchain and IoT to create a decentralized system for monitoring the SoC of EV batteries in real-time. This application used two blockchain platforms, Ethereum and IOTA Tangle, to manage and verify transactions related to battery data and requests for charging or swapping services. These blockchain systems enabled M2M communication, where vehicles and charging stations exchanged information directly without requiring human intervention. Utilizing M2M allows EVs to autonomously detect when battery swapping or charging is required and facilitates efficient energy management across a decentralized network of stations. Their study not only improved the convenience of EV operation but also enhanced the reliability and security of BMS in real-time scenarios [258].

One of the primary challenges in establishing WSNs for LiB applications is the development of energy-efficient protocols and data reliability for effective deployment in commercial and industrial applications. Sensor nodes, typically powered by limited battery resources, consume substantial energy during data transmission, leading to network lifetime constraints [255]. Strategies such as duty cycling, sleep scheduling, and data aggregation have been proposed to mitigate energy consumption [259–261]. However, achieving a balance between energy savings and network performance remains a complex task. Data reliability faces additional challenges due to signal interference, environmental conditions, and hardware limitations, which can cause data loss or inaccuracies [262]. Ensuring consistent and accurate data transmission necessitates robust error detection and correction mechanisms, as well as fault-tolerant network protocols. Furthermore, these wireless communications confront inherent limitations in transmission power, speed, channel capacity, and susceptibility to electromagnetic interferences, which hinder the effectiveness of WSNs [263]. Advancements in energy-efficient communication protocols, such as clustering and routing algorithms, are essential for extending the network lifespan without compromising data integrity [264]. In a study, Lajara et al. proposed a method to predict the SoH of batteries used in WSNs. The study aimed to create a simple, computationally efficient model, multilayer perceptron (MLP), capable of estimating the battery degradation over time-based on various parameters such as voltage, current, temperature, and the number of charge/discharge cycles [254]. The proposed model was tested on Telosb motes, and the results showed that MLP models outperformed simpler methods, including least squares regression and linear regression, providing higher accuracy in tracking the SoH. The experimental validation revealed that the MLP model achieved an absolute error of 0.001 and a relative error of 0.7% across a range of conditions, demonstrating its robustness in predicting battery health, even under varying environmental conditions, such as temperature fluctuations (from − 16 to 45 °C). These findings revealed that the model can accurately estimate the battery SoH with low computational cost in low-power sensor nodes in WSNs applications [254]. The models’ ability to handle these diverse factors ensures their practical application in real-world WSNs, where energy efficiency and battery longevity are critical. Furthermore, these models showed robustness in predicting SoC/SoH for batteries with varying capacities, suggesting their versatility in different use cases, such as remote sensing and environmental monitoring in energy-constrained scenarios.

Similarly, Ali et al. developed an adaptive method for estimating the SoC of batteries in WSNs using GPR [265], particularly targeting low-power devices such as sensor nodes commonly used in commercial and industrial applications. Their study addressed the challenge of accurate SoC estimation for devices operating under variable environmental conditions, including temperature fluctuations and diverse battery chemistries. The data was collected in a controlled laboratory environment, testing batteries under various temperatures (from 5 to 45 °C) and across different types (Li-ion, nickel-metal hydride, and Li-polymer). Their findings showed that the GPR model outperformed other methods, such as polynomial regression and SVM, with the GPR model achieving an MAE of 2.53% for nickel-metal hydride batteries, 2.54% for lithium-polymer, and 2% for LiBs at 25 °C. The corresponding root mean square error (RMSE) values were 0.295**,** 0.292, and 0.35, respectively. This adaptive GPR model demonstrated strong robustness and accuracy for real-time online SoC estimation on low-power embedded platforms, such as ARM Cortex M4-based microcontrollers, validating its practical use for WSN applications [265]. The adaptability of their model to different battery chemistries and temperature ranges, combined with its computational efficiency, suggests its integration into BMS and other industrial applications requiring battery monitoring.

## Current Trends and Future Prospects of Sensor Technologies for LiBs

### Market Trends and Industry Developments

The global battery monitoring systems market is projected to grow robustly, driven by escalating demand for LiBs in EVs, renewable energy storage, and consumer electronics. This growth is underpinned by advancements in sensor technologies that enhance safety, efficiency, and predictive capabilities. According to future market predictions, the global battery monitoring systems market is poised for substantial growth, estimated to reach USD 5.5 billion by 2030, with a compound annual growth rate (CAGR) of 18.7% during 2022–2030 [266]. This projected growth can be attributed to factors such as the surge in several EVs, increasing demand for consumer electronics, and the increasing need for battery safety and optimization, particularly in the renewable energy sector [267–269]. A key trend is the development of multifunctional sensors capable of simultaneous real-time monitoring of temperature, pressure, and strain, providing accurate data to prevent battery failures caused by overcharging, overheating, and mechanical stress. For example, optical FBG sensors, due to their small dimensions (Ø < 200 µm), immunity to electromagnetic interference, and corrosion resistance, have shown exceptional effectiveness in detecting chemo-mechanical stresses during LiBs operation, enabling accurate tracking of internal strain and temperature gradients through minimally invasive measurements [1, 148]. Similarly, integrating IoT and WSNs into BMS allows cloud-based analytics, remote diagnostics, and firmware updates, facilitating proactive maintenance and reducing potential failures in LiB systems [226, 252, 270–273]. Khawaja et al. explored the application of artificial intelligence in enhancing BMS for LiBs [226]. They focused on estimating two critical battery parameters, SoC and SoH, by employing six ML algorithms, including ANN, RF, gradient boost, light gradient boosting machine (Light-GBM), extreme gradient boosting (XGB), and SVM. Their study found that among these algorithms, the RF regressor achieved the highest accuracy, with an R^2^ score of 0.9999 and minimal errors in MAE (0.0035), median absolute error (0.0013), and RMSE of 0.0097. These results demonstrated the potential of artificial intelligence, particularly ML techniques, to significantly improve the accuracy of SoC and SoH predictions, which is crucial for optimizing the performance and safety of LiBs in EVs [226]. In addition, strategic collaborations between industry leaders (e.g., Tesla, Panasonic, LG Chem, and Samsung SDI) and academic institutions are accelerating the adoption of self-powered sensors that harvest energy from battery operations. These sensors eliminate external power needs while preserving energy density. Innovations, including IoT, ML algorithms, and piezoelectric nanogenerators embedded in battery cells, exemplify this trend, enabling continuous health monitoring without compromising performance [274]. The prospects of LiB sensor technology appear promising, with reduced energy losses, enhanced efficiency, decreased weight and cost, and ensured more reliable operation under extreme conditions.

### Emerging Research Directions and Opportunities

Recent advances in nanomaterials, such as graphene, CNTs, and MOFs, are revolutionizing LiB sensor design by offering high sensitivity and low power consumption, surpassing conventional rigid metal- and semiconductor-based LiB sensors [187, 275, 276]. Graphene-based sensors possess high conductivity and mechanical flexibility, making them ideal for ultra-sensitive strain detection in electrode materials. Their capability to detect gases and biomolecules, combined with techniques such as functionalization and hybridization, significantly enhances their performance [65, 275, 277]. For example, Bree et al. explored the use of surface-mounted thin-film graphene sensors to monitor the SoC and volume expansion in LiBs [275]. They developed a sensor based on a percolative graphene film that detected small changes in the electrical resistance of the film caused by the expansion and contraction of electrode materials during charge and discharge cycles. In addition, due to degradation processes, the sensor could detect early signs of irreversible cell expansion, such as gas generation or Li plating, providing valuable early warnings of potential failures. Their study demonstrated that the highly sensitive graphene sensors can detect small volumetric changes that correlate with SoC, facilitating timely interventions to prevent catastrophic cell failure [275]. Chen et al. developed a novel in situ pressure measurement technique for large-format LiBs using flexible thin-film pressure sensors embedded within the battery’s jelly roll structure [65]. Their study focused on monitoring the mechanical pressure evolution during the battery’s operation, as the pressure change in the jelly roll reflected the internal mechanical behavior critical to battery performance and safety. The sensitive layer consisted of thermoplastic polyurethane (TPU), graphite, and CNTs, all of which worked as flexible materials in the sensor fabrication. Their study demonstrated that these pressure sensors could detect both reversible and irreversible pressure variations associated with the lithiation and delithiation of the electrodes, providing valuable data for understanding the battery’s mechanical performance and potentially detecting issues such as electrode deformation or short circuits [65].

Furthermore, advancements in LiBs monitoring have demonstrated the efficacy of MOF-based chemiresistive sensors to detect electrolyte decomposition byproducts (e.g., DMF) at ppb-level concentrations [187]. Lu et al. developed ultra-sensitive capacitive chemical sensors of IC-MOFs thin films for detecting electrolyte leakage in LiBs [187]. These sensors could signal a leak while the voltage of the leaking cell remained almost the same level as that of a pristine cell. Their study demonstrated excellent sensing responses, effectively detecting trace amounts of DMC vapors (50 ppb) and electrolyte leakage (20 nL) from LiBs with a *t*_*90*_ of less than 2 s of exposure [187]. Recent reports indicate that at higher temperatures, electrons in the valence band of semiconductors gain energy and transition to the conduction band. This movement enhances the number of charge carriers, resulting in a decrease in electrical resistance [278]. In contrast, the electrical resistance of carbon-based materials, such as CNTs, varies with temperature, allowing for the development of highly sensitive and accurate temperature sensors [278]. In a study, Zhang et al. developed a flexible integrated temperature–pressure sensor using CNTs to monitor the TR of LiBs [279]. They utilized the unique resistance–temperature properties of CNTs and nickel (Ni) to create a dual-parameter sensor, CNT/Ni/PVP/GF fiber, capable of decoupling temperature and pressure measurements simultaneously at the same point on the battery surface. The sensor demonstrated temperature sensitivity across a range of 20–100 °C and pressure sensitivity up to 200 kPa. Their design included a circular interdigital electrode to enhance pressure sensitivity and minimize temperature interference. The sensor’s ability to decouple temperature and pressure responses was validated through TR tests, showing its potential for real-time detection of unsafe conditions in LiBs, such as overheating or swelling, which were precursors to TR [279]. Similarly, Sun et al. developed a strain-resistant, flexible thermistor sensor array using hybrid CNTs and MXene materials, specifically for LiBs and human temperature monitoring [280]. The CNT/MXene hybrid materials exhibited excellent thermosensitivity, with a temperature coefficient of resistance (TCR) of − 0.52% °C^−1^, and the sensor demonstrated high precision and fast response times. The sensor array showed remarkable mechanical flexibility and durability, maintaining its performance after over 2,000 bending cycles. The device could monitor temperature changes across a wide range, from − 20 to 220 °C, making it ideal for real-time monitoring of LiBs. It was successfully applied to commercial LiBs, providing accurate temperature readings during charge and discharge cycles, enabling overheating detection, and optimizing the battery’s performance and safety [280].

Self-repairing sensors signify a paradigm shift in durability for harsh battery environments. These sensors integrate smart materials inspired by self-repairing approaches using sacrificial weak bonds. These materials, based on biomolecules or polymers, can self-repair through dynamic supramolecular self-assembly, involving mechanisms such as non-covalent hydrogen (H) bonding, ionic bonding, and host–guest interactions [281–284]. Betermier et al. investigated using cyclodextrins in combination with protein nanopores as an innovative method for discriminating polysulfide species at the single-molecule level to advance self-repairing functionalities in LiBs [281]. The study demonstrated the ability of cyclodextrins to reversibly form inclusion complexes with polysulfides of varying lengths (Na_2_S_2_, Na_2_S_3_, Na_2_S_4_, and Na_2_S_5_), providing a precise molecular recognition mechanism. They achieved single-sulfur atom resolution discrimination, as indicated by the distinct ionic current blockades when interacting with α-hemolysin nanopores embedded in lipid membranes. Notably, the β-cyclodextrin displayed a superior affinity for longer-chain polysulfides, evidenced by an association constant (K_β5_) of 181 ± 4 M^−1^ for Na_2_S_5_, clearly surpassing the affinity toward shorter polysulfides. This precise discrimination method could be adapted for real-time battery electrolyte monitoring, facilitating self-repair by selectively capturing unwanted polysulfide species [281]. Similarly, developing polymers with dynamic covalent bonds (e.g., Diels–Alder adducts, Schiff-base imines, disulfides, etc.) form the backbone of modern self-repairing sensors. Without external repair, these sensors autonomously repair damage, such as cracks or breaks caused by mechanical stress or thermal cycling [284]. These bonds can break and reform under stimuli (i.e., heat, pressure, or other triggers), allowing a cracked sensor network to repair itself and recover electrical pathways. This technology promises smart LiBs with embedded sensors that remain reliable despite harsh EV conditions [285]. For example, Dodo et al*.* reported a flexible dynamic polymer nanocomposite (DPNs) sensor using an interpenetrating network with Diels–Alder crosslinks and multiwalled CNTs as conductive nanofillers [284]. The reversible Diels–Alder bonds allowed repeated healing triggered by moderate heat at about 90 °C for 24 h, while the CNT network provided high piezoresistive sensitivity. The sensor achieved a gauge factor of 27 ± 3 at 60% strain, indicating high strain sensitivity [284]. Notably, after the composite was cut and then thermally healed, its electrical continuity was restored, allowing a connected light-emitting diode (LED) to lit up again, effectively regaining its functionality. Their study demonstrated near-complete performance recovery post-healing process. The study highlighted that such dynamic networks can endure multiple cut/heal cycles with minimal loss in mechanical strength or conductivity, indicating approximately ∼ 90% stress relaxation over time [284]. The Battery 2030 + roadmap emphasizes integrating high-sensitivity sensors at the cell level and even coupling them with battery self-repairing mechanisms. By providing durable, long-lived sensing, these materials transform smart LiBs into systems that can detect and respond to real-time internal changes, enhancing their lifespan, safety, and performance. Integrating two research domains, the battery interface genome (BIG) and the materials acceleration platform (MAP), into the BIG–MAP framework will transform our approach to understanding and discovering new battery materials and interfaces [285].

Moreover, emerging sensors are addressing sustainability challenges by enabling efficient battery repurposing. X-ray transmission paired with ML algorithms has been used to classify degraded cells for second-life storage applications, while hyperspectral imaging (HSI) sensors automate the detection of valuable metals (e.g., Co, Au) during recycling [286–288]. In a study, Ueda et al. developed an in-line sorting system that integrated X-ray transmission scanning with deep learning to detect batteries within electronic waste [286]. The system employed a three-stage deep learning process: first, it estimated the type of e-waste item from X-ray images; second, it detected batteries using networks pre-trained for the identified item types; and third, it identified any overlooked batteries through a follow-up network trained on diverse scenarios. This approach achieved high accuracy rates, with 96.7% for trained e-waste categories and 77.0% for untrained categories, surpassing the performance of single-network systems, which obtained 90.2% and 71.6%, respectively [286]. Similarly, Richter et al. investigated the spectral characterization of components in end-of-life LiBs, focusing on optical sensors for recycling [287]. They utilized five reflectance sensors across the visible to long-wave infrared spectrum to identify the best spectral range for detecting key battery components, including aluminum (Al), copper (Cu), and plastic. Their findings included a spectral library, revealing that the visible to near-infrared range (400–1000 nm) is optimal for differentiating materials in the recycling process. They also examined hyperspectral imaging (HSI) sensors, highlighting their importance for monitoring mechanical sorting in battery recycling, which aids in developing automated sorting systems for efficient battery recycling [287].

### Future Prospects

The rapid evolution of LiBs demands advanced sensor technologies to enhance safety, efficiency, and lifespan. A recent study by Han et al. introduced an integrated sensor utilizing low-temperature co-fired ceramic (LTCC) technology for real-time internal pressure and temperature monitoring within LiBs [289]. This sensor combined a multilayer ceramic circuit board with embedded MEMS pressure and digital pulse temperature sensors, achieving precise measurements with a pressure resolution of 1 kPa and temperature resolution of 0.1 °C, even under harsh electrolyte exposure. The sensor’s effectiveness was demonstrated by embedding it into pouch-type and cylindrical prototype batteries. It successfully captured critical events such as periodic internal pressure variations linked to Li-ion intercalation processes and distinct temperature and pressure changes associated with battery degradation and swelling. Furthermore, the sensor maintained high stability after 60 days of immersion in the corrosive electrolyte, underscoring its potential for long-term battery health monitoring [289]. Building upon this advancement, future research direction can focus on a roadmap outlining specific priorities as follows:

(1) Development of multi-modal, minimally invasive sensing systems, integrating FBG and LTCC sensors. FBG sensors offer advantages such as low invasiveness, resistance to electromagnetic interference, and the ability to simultaneously monitor multiple parameters, such as internal temperature, strain, gas emissions (e.g., CO_2_, H_2_), and electrochemical states (e.g., SEI growth, Li plating), within LiBs. The key priorities of this system should include;

Optimize wireless, miniaturized, self-powered sensors with self-calibration to mitigate signal drift at high cycles and self-repairing functionality (e.g., conductive hydrogels).

Ensure compatibility with diverse LiB chemistries (e.g., NMC, LFP, solid-state).

Developing hybrid physics-based and ML models (e.g., LSTM networks, GPR) can link sensor data to aging mechanisms and accurately predict battery SoH.

(2) Innovation in sustainable sensor materials and scalable manufacturing;

Replace non-recyclable polymers (e.g., PDMS) with biodegradable composites (e.g., nanocellulose, stable at > 150 °C, resistant to electrolyte corrosion) to reduce environmental footprints and utilize scalable manufacturing processes (e.g., roll-to-roll printing) to facilitate the mass production of sensors.

Leveraging the battery interface genome-materials acceleration platform (BIG-MAP) can accelerate sensor material discovery and validation. BIG-MAP integrates autonomous robotics, computational tools for SEI prediction, and shared data infrastructure to streamline the development process [148].

(3) Collaborative efforts to unify data standardization;

Collaborating with initiatives such as BATTERY 2030 + to establish standardized data protocols can ensure interoperability with next-generation BMS [290].

Establishing benchmarks for multi-sensor fusion (e.g., integration of optical + electrochemical signals) and validating performance in commercial formats (i.e., pouch, cylindrical) can contribute to the development of safer, smarter LiBs.

## Potential Challenges and Opportunities of Current LiB Sensor Technology

### Cost-Effectiveness and Scalability of Sensor Technology

The present cost of sensor technology for LiB poses significant challenges and limitations. The manufacturing costs of these sensors, particularly for custom sensors, are high due to intricate production processes. Furthermore, integrating sensors into LiBs can escalate the cost of the final battery product. The maintenance and calibration of sensors entail periodic expenses that cumulatively burden the LiB life cycle economics. The process of scaling up sensor manufacturing can present industrial hurdles necessitating substantive capital investments, potentially deterring the proliferation of sensor integration in battery systems and, hence, stifling advancement. The integration of sensors into the LiBs can also be complex, necessitating multidisciplinary expertise in both sensor technology and battery engineering. These technical and fiscal complexities could marginalize enterprises lacking in resource diversity or technical breadth [186, 291, 292]. Despite the existing challenges, ongoing research and development initiatives focus on establishing cost-efficient and scalable sensor technologies for LiB, particularly in the form of 3D printing and flexible sensors. The implementation of AI and ML algorithms to automate sensor calibration and maintenance, thus reducing the need for manual intervention, also holds promise in decreasing maintenance costs [76, 291–295].

### Sensor Durability and Compatibility with LiB Chemistry and BMS Structure

The compatibility between sensors and evolving LiB chemistries, such as solid-state and lithium-air systems, presents a multifaceted challenge that demands rigorous interdisciplinary research. Sensors integrated into these advanced battery systems must operate reliably under harsh chemical, thermal, and mechanical conditions while maintaining chemical stability, sensitivity to dynamic parameter shifts, and minimal interference with electrochemical processes [295, 296]. For example, in solid-state Li-metal batteries, sensors must withstand the reactivity of Li metal and the evolving SEI, necessitating materials with exceptional corrosion resistance [1, 122]. Recent studies showed the successful embedding of FBG sensors within solid-state coin cells to monitor mechanical stresses during cycling, demonstrating progress in addressing chemical compatibility [297]. Similarly, mechanical integrity under repeated volume changes during charge–discharge cycles remains critical, as sensors must endure physical deformations without compromising accuracy. Embedded strain sensors have enabled real-time monitoring of internal stresses, offering insights into failure mechanisms [298, 299]. For example, Albero Blanquer et al. embedded FBG strain sensors into liquid and solid-state electrolyte LiB cells to measure real-time chemo-mechanical stress during battery cycling. The authors successfully correlated the shifts in optical signals (*Δλ*) to mechanical stress (*Δσ*), revealing critical insights into the internal stress evolution during the charging and discharging cycles of the batteries [1]. For example, in the case of InLix-based electrodes, they observed a nearly linear stress variation during the Li insertion and extraction cycles, with a reversible stress pattern indicating high mechanical stability. Their study revealed the potential of FBG sensors in providing localized, internal stress measurements that were previously unattainable with external force sensors [1].

Electrochemical interference further complicates sensor integration, as foreign materials or sensing mechanisms risk disrupting ion transport or side reactions [300]. Innovations such as piezoelectrochemical transducers show promise by converting mechanical deformations into measurable electrical signals without perturbing electrochemical performance [301]. Concurrently, stringent size and weight constraints imposed by compact BMS drive advancements in micro- and nano-fabrication. Thin-film sensors and nanotechnology-enabled designs exemplify progress in miniaturization, balancing sensitivity with minimal spatial footprint [209, 302]. Solid-state sensors, leveraging robust materials including ceramics or composites, are particularly suited for high-stability applications, while nanomaterials enhance sensitivity to minute parameter changes, such as early gas emissions or microscale strain variations [282, 302]. Multi-physical sensing systems, integrating thermal, acoustic, and gas detection capabilities, further augment holistic monitoring, enabling BMS to predict failures and optimize performance [301].

The synergy between sensors and BMS is pivotal, as ML algorithms process real-time data to enable predictive maintenance and hazard mitigation. For example, temperature and pressure sensors coupled with adaptive algorithms can preempt TR by triggering safety protocols [109]. Future advancements hinge on material innovation, such as developing chemically inert yet responsive sensing materials and refining fabrication techniques to enhance scalability and integration [303]. Hybrid strategies combining advanced sensors with modular BMS architectures could further improve system responsiveness and data fidelity [282, 301]. Addressing these challenges requires a concerted focus on interdisciplinary collaboration, bridging materials science, electrochemistry, and data analytics to ensure sensors evolve in tandem with next-generation battery chemistries [303]. Such efforts will be critical to achieving durable, high-fidelity monitoring systems that enhance the safety, efficiency, and longevity of smart LiB applications in automotive and beyond.

### Balancing Sensor Integration and LiB Energy Density Trade-offs

Moreover, integrating sensors into LiBs poses a challenge due to the limited space within the battery system, which is crucial for maintaining or enhancing energy density, particularly in applications such as EVs and portable electronics, where LiBs are used for high-energy-demand operations [199, 304]. These applications extremely rely on maximizing the energy density of LiB to maximize operational time and minimize weight. Therefore, any reduction in energy density resulting from sensor integration should be carefully evaluated to ensure that the sensor capability surpasses the potential drawbacks of reduced energy capacity. Various approaches can be employed to mitigate the trade-offs associated with energy density. One approach involves addressing the miniaturization and integration challenges associated with sensors, enabling sensor integration without significantly compromising the overall energy density. Moreover, by optimizing sensor design and placement, the impact on energy density can be minimized, ensuring the enhancement of overall battery performance. Additionally, using low-power and miniaturized sensors, such as MEMS-based sensors, can minimize energy consumption while maintaining accurate monitoring capabilities. Similarly, the development of ML algorithms can enable efficient utilization of sensor data, reducing the need for excessive sensor integration and potentially improving energy density.

### Environmental Impact of LiB Sensor Manufacturing and Recycling

The considerable surge in LiB sensors, driven by technology and industry advancements, can pose significant sustainability challenges throughout the sensor life cycle. One major contributor to these challenges is the extraction of raw materials, such as metals and rare earth elements. This extraction leads to habitat destruction, soil erosion, and water pollution. Furthermore, manufacturing LiB-powered sensors are energy-intensive, resulting in notable carbon emissions. The parametric life cycle assessment (LCA) by Bunyui Manjong et al. revealed reference emissions of 107 kg CO_2_ kWh^−1^ for LFP and 94 kg CO_2_ kWh^−1^ for NMC811 cells under global average raw material and energy conditions [305]. However, carbon emissions varied widely: LFP cells produced in Norway with optimal parameters (high ore grades, 98% material recovery, low-carbon electricity) achieved 27 kg CO_2_ kWh^−1^, while this value in China reached 127 kg CO_2_ kWh^−1^ [305]. For NMC811, emissions spanned 27–155 kg CO_2_ kWh^−1^, mainly driven by nickel sulfate (NiSO_4_) production. Their results indicated that key material contributors included aluminum for LFP and nickel for NMC811, underscoring the necessity of decarbonizing raw material extraction and refining. Their study emphasized that achieving low-carbon LiBs requires holistic strategies addressing ore grades, material efficiency, technology upgrades, and renewable energy adoption across global supply chains [305].

Semiconductor fabrication for sensor chips and integrated circuits is resource-intensive, requiring substantial electricity and water while emitting potent GHGs and even releasing hazardous fumes or waste (e.g., solvents, flux). Similarly, improper disposal of LiB-enabled sensors presents environmental and safety hazards. When LiB cells or sensors are improperly discarded in general waste or recycling systems, physical damage (e.g., crushing or puncturing) can trigger fires or leak toxic electrolytes and heavy metals. In landfills or informal recycling processes, these components risk leaching plastics, lithium compounds, and other pollutants into soil and groundwater, undermining circular economy goals. Challenges such as low collection rates, non-removable batteries, and the persistence of hazardous materials further complicate efforts to establish sustainable end-of-life pathways for sensor technologies [306, 307].

Transitioning to a circular economy for LiB sensors will necessitate design and system changes that facilitate reuse and recycling. One significant opportunity lies in adopting greener alternatives that utilize biodegradable or organic components. For example, battery electrodes and sensor components can incorporate bio-based polymers (e.g., cellulose or other biomass-derived binders) that provide necessary conductivity while being biodegradable [308]. Even the LiB packaging itself can be made from bio-plastics derived from renewable resources, which naturally break down over time, reducing plastic waste from discarded sensors [308]. Further opportunity is to eco-design for disassembly, which involves designing devices that can easily remove and replace batteries, sensors, and key components. This requirement encourages manufacturers to move away from permanently sealed-in batteries, allowing consumers or recyclers to extract battery components before disposal.

Regulatory bodies and industry leaders have recognized the need to improve the sustainability of batteries and sensors. Various initiatives and new regulations are accelerating the adoption of sustainable practices throughout the life cycle of LiB-powered sensors. For example, the European Union has introduced comprehensive regulations to govern the entire battery life cycle, which affects LiB sensor devices. Notably, this regulation requires that by 2027, all portable batteries in appliances must be user-removable and replaceable [309]. This is a direct push against sealed, disposable sensor gadgets, prompting the design of sensors for end-of-life recovery and the incorporation of recycled materials into battery production. Leading tech companies have launched initiatives to reduce the environmental footprint of their devices, including those with LiBs. For example, Apple Inc. has committed to using recycled materials at unprecedented levels. By 2025, Apple vows to use 100% recycled cobalt in the batteries of its products [310]. Apple also reports using recycled tin solder and gold plating on circuit boards [310]. These efforts reduce the demand for newly mined metals and cut down the life-cycle impacts of their sensors and gadgets. Battery recycling companies, including Redwood Materials, are now able to recover over 95% of critical metals from LiBs cells and sensors [311]. Recovered lithium, nickel, cobalt, copper, etc., are refined and supplied back to battery manufacturers, effectively creating a circular supply chain for battery materials [311].

## Conclusion

The critical role of LiB in the growing EVs and renewable energy sector necessitates a relentless pursuit of safety and efficiency. This review article has elucidated the pivotal function of advanced sensor technologies in BMS and their significance in enhancing the performance, longevity, and intrinsic safety of smart LiB. In the integration of sensors within LiB systems, a more resilient energy storage solution can be achieved through meticulous surveillance of critical parameters. As we approach a technologically transformative era, the emergence of innovative sensor technologies, driven by miniaturization, cutting-edge nanomaterials, and the application of ML algorithms and wireless sensing paradigms, marks a new epoch. These advancements not only promise to mitigate energy inefficiencies but also streamline battery operation, even under challenging environmental conditions. The convergence of accuracy, responsiveness, and predictive maintenance in LiB secures their position as a backbone for a sustainable and electrified future.

However, this technological approach is not without formidable trials. The compatibility of sensor technologies with ever-evolving battery chemistries, the concern for sensor durability, the economic considerations of cost and scalability, balancing sensor integration with LiB energy density trade-offs, and environmental impact represent significant barriers on the path to universal LiB sensor implementation. Overcoming these challenges requires collaborative efforts from researchers, engineers, and industry stakeholders to transform the landscape of LiB sensor technology. In conclusion, the path of LiB sensor advancements presents, at its core, an incorporation of potential, challenge, and opportunity. As our reliance on EVs and renewable energy continues to grow, it becomes imperative for us to fully harness the capabilities of sensor technology. Through collaboration and unwavering curiosity, we can optimize the performance and safety of LiBs, reinforcing the foundational pillars of an environmentally conscious and energy-secure society.

**Table:** List of nomenclature and acronyms

Name	Nomenclature	Acronym	Chemical formula
Lithium-ion	–	Li-ion	Li^+^
Lithium cobalt oxide	Lithium(I) cobalt(III) oxide	LCO	LiCoO_2_
Lithium titanate	Lithium oxido(oxo)titanium	LiTi	Li_4_Ti_5_O_12_
Copper/Nickel	–	Cu/Ni	Cu^+^/Ni^+^
Lithium nickel manganese cobalt oxide	Lithium Nickel (8 parts)-Cobalt (1 part)-Manganese (1 part) oxide	NCM811 or NMC	LiNi_0.8_Co_0.1_Mn_0.1_O_2_
Lithium nickel cobalt aluminum oxide	Lithium nickel cobalt aluminum oxide	NCA	LiNiCoAlO_2_
Trinitrotoluene	2-Methyl-1,3,5-trinitrobenzene	TNT	C_7_H_5_N_3_O_6_
Ethylene	Ethene	ET	C_2_H_4_
Polyvinylidene fluoride	Poly(1,1-difluoroethylene)	PVDF	C_2_H_2_F_2_
Sulfur dioxide	Sulfur dioxide	SO_2_	SO_2_
Lithium iron phosphate	Lithium iron (II) phosphate	LFP	LiFePO_4_
2-(2-hydroxyphenyl) naphthoxazole	2-(2-hydroxyphenyl) naphtho[2,1-b] oxazole	HPNO	C_18_H_11_NO_2_
Poly(dimethylsiloxane)	Poly(dimethylsiloxane)	PDMS	C_2_H_6_OSi
9,10-dimethylanthracene	9,10-dimethylanthracene	DMA	C_16_H_14_
Lithium manganese oxide	Lithium manganese (IV) oxide	LMO	LiMn_2_O_4_
Lithium intercalated graphite	–	LIG	Li_x_C_6_
Hydroxyl group	–	–OH	–OH
Amino group	–	NH	–NH_2_
Methyl group	–	CH	H_3_C
Lithium hexafluorophosphate/ ethylene carbonate/ diethyl carbonate	Lithium hexafluoridophosphate ion/1,3-dioxolan-2-one/Ethyl carbonate	LiPF_6_/EC/DEC	LiPF_6_/C_3_H_4_O_3_/C_5_H_10_O_3_
Poly(anthraquinonyl sulfide)	Poly(anthraquinonyl sulfide)	PAQS	–
Dimethyl carbonate	Dimethyl carbonate	DMC	C_3_H_6_O_3_
Aluminum oxide	Aluminum (III) oxide	Al_2_O_3_	Al_2_O_3_
Copper dioxide	Copper (II) oxide	CuO	CuO
1,3-dioxolan	1,3-dioxolan	DOL	C_4_H_8_O_2_
1,2-dimethoxyethane	Ethylene glycol dimethyl ether	DME	C_4_H_10_O_2_
Ethyl methyl carbonate	Ethyl methoxycarbonylformate	EMC	CH_3_OCO_2_C_2_H_5_
Poly- (vinylidene fluoride-trifluoroethylene)	Poly(1,1-difluoroethylene-1,2,2-trifluoroethylene)	PVDF-TrFE	(C_2_H_2_F_2_)-(C_2_HF_3_)
Triethyl 1,3,5-triazine-2,4,6-tricarboxylate	Ethyl 2,4,6-tris(ethylamino)-1,3,5-triazine-2,4,6-tricarboxylate	TETAT	C_12_H_18_N_3_O_6_
Vinylene carbonate	1,2-epoxy-3-propenyl carbonate	VC	C_3_H_2_O_3_
Poly-(anthraquinonyl sulfide)	Poly-(anthraquinonyl sulfide)	PAQS	(C_14_H_6_O_2_S)*n*
Fluorinated dimethoxybutane	1,1-difluoro-2,3-dimethoxybutane	FDMB	–
Lithium bis(fluorosulfonyl)imide	Lithium bis(fluorosulfonyl)imide	LiFSI	LiN(SO_2_F)_2_
Lithium phosphorus oxynitride	Lithium phosphorus oxynitride	–	Li_x_PO_y_N_z_
Lithium nickel oxide	Lithium nickel oxide	LNO	LiNiO_2_

## Data Availability

This review article does not generate new data. All data and information discussed are derived from previously published studies, which are appropriately cited in the manuscript.

## Abbreviations

LiB: Lithium-based batteries
mAh: Milliampere-hours
EV: Electric vehicles
BMS: Battery management systems
ML: Machine learning
FBG: Fiber bragg grating
TR: Thermal runaway
CT: Computed tomography
TC: Thermocouple
RTD: Resistance temperature detectors
TFRTD: Thin-film resistance temperature detector
TFTC: Thin-film thermocouples
*V*: Volt
C-rate: Charge discharge current rates
SoH: State-of-health
SoC: State-of-charge
POF-FBG: Polymer optical fiber-fiber bragg grating
NDIR: Nondispersive infrared
AE: Acoustic emission
UT: Ultrasonic testing
AMR: Anisotropic magnetoresistive
ME: Magnetoelectric
NEM: Noise equivalent magnetic
pT: Picotesla
MNPT: Magnetic nanoparticle thermometer
DC: Direct current
OCV: Open circuit potential
SEI: Solid electrolyte interphase
VOC: Volatile organic component
IC-MOF: Ionically conductive-metal-organic framework
R_CT_: Charge transfer
EIS: Electrochemical impedance spectroscopy
TCM: Terahertz chemical microscopy
AC: Alternating current
DFOS: Distributed fiber optic sensor
DRS: Diffuse reflectance spectroscopy
DFT: Density functional theory
DTW: Dynamic time-warping
AIMD: Ab-initio molecular dynamics
OSV: Organic solvent vapor
EMCCD: Electron-multiplying charge-coupled device
XRS: X-ray Raman scattering
HC: Hollow-core
IR: Infrared
ATR-IR: Attenuated reflectance infrared
CT: Computed tomography
3D-DIW: 3D-direct ink writing
ALD: Atomic layer deposition
MEMS: Micro–electro–mechanical system
BIG-MAP: Battery interface genome-materials acceleration platform
M2M: Machine-to-machine
RMSE: Root mean squared error
MAE: Mean absolute error
LTCC: Low-temperature co-fired ceramic
LSTM: Long short-term memory
RUL: Remaining useful life
GRR: Gaussian process regression
ANN: Artificial neural networks
SVM: Support vector machines
VTS: Virtual thermal sensors
PHM: Prognostics and health management
CNN: Convolutional neural networks
WSN: Wireless sensor networks
GPR: Gaussian process regression
CAGR: Compound annual growth rate
IoT: Internet of things
UV-vis: Ultraviolet-visible
TM: Transition metals
CNT: Carbon nanotubes
*λ*_*B*_: Bragg resonance
BESS: Battery energy storage system
CV: Cyclic voltammetry
*λ*ex: Excitation wavelength
CCD: Charge coupled device
RUL: Remaining useful life
pm με: Picometers per microstrain
µm^2^: Square micrometers
ppb: Parts per billion
SMF: Single-mode fiber
QRF: Quantile regression forests
MOF: Microstructure optical fiber
PCA: Principal component analysis
LDA: Linear discriminant analysis
DNN: Deep neural networks
SPE: Solid polymer electrolyte
SSE: Solid-state electrolyte
TFB: Thin-film microscale batteries
ΔS: Entropy coefficient
BITS-BMS: Battery internal temperature tensor-based BMS
FPI: Fabry–Perot interferometer
BITS-BMS: Battery internal temperature sensor-based BMS
